# RACK1 maintains mouse hematopoietic stem cells by directly binding to and stabilizing LDB1

**DOI:** 10.1038/s41419-026-09177-z

**Published:** 2026-08-09

**Authors:** Lijiao Deng, Junjie Du, Zhe Xu, Lu Guo, Qingyang Wang, Anbiao Wu, Guang Yang, Yu-chen Lu, Tingting Guo, Chunxiao Niu, Junxia Cao, Yu Lan, Bingshui Xiu, Jiannan Feng, Jiyan Zhang

**Affiliations:** 1 Beijing Institute of Basic Medical Sciences, Beijing, China; 2 National Engineering Research Center for the Emergency Drugs, Beijing Institute of Pharmacology and Toxicology, Beijing, China; 3 Chinese PLA Medical School, Chinese PLA General Hospital, Beijing, China; 4 State Key Laboratory of Experimental Hematology, Haihe Laboratory of Cell Ecosystem, Institute of Hematology, Senior Department of Hematology, The Fifth Medical Center of PLA General Hospital, Beijing, China; 5 Department of Basic Medical Sciences, Anhui Medical University, Hefei, China; 6 Key Laboratory for Regenerative Medicine of Ministry of Education, Institute of Hematology, School of Medicine, Jinan University, Guangzhou, China; 7 Chinese Institute for Brain Research, Beijing, China

**Keywords:** Cell death, Haematological diseases, Haematopoiesis

## Abstract

A core complex with transcription factors (TFs) TAL1/TCF3/GATA2 and adaptors LMO2/LDB1 lies at the top of the hematopoietic transcriptional hierarchy. The mechanism(s) underlying the expression of these components remain elusive. Adaptor RACK1 interacts with multiple TFs and modulates their activation and/or stability. However, a role of RACK1 in the transcriptional control of hematopoietic stem cell (HSC) fates hasn’t been disclosed. Here, we report that RACK1 is expressed across various hematopoietic cell types. Adulthood *Rack1* deletion in type I interferon- (IFN-I)-responsive cells leads to rapid and profound hematopoietic failure and HSC loss. HSC exhaustion upon adulthood *Rack1* deletion results from cell-intrinsic defects with massive apoptosis. Single-cell RNA sequencing indicates that adulthood *Rack1* deletion in IFN-I-responsive cells leads to aberrant lineage-geneset-scores of transcriptional HSCs and the emergence of stressed HSCs. Furthermore, prenatal deletion of *Rack1* in hematopoietic cells results in reduced and defective HSCs in the fetal liver. Mechanistically, RACK1 prevents HSC loss through maintaining the protein level of LDB1. The direct interaction between RACK1 and LDB1 suppressing its ubiquitination and subsequent degradation, thereby stabilizes LDB1. Therefore, RACK1 maintains adult and fetal mouse HSCs through, at least partially, directly binding to and stabilizing LDB1.

## Introduction

With high capability of self-renewal and differentiation, hematopoietic stem cells (HSCs) maintain the generation of all blood cells. Numerous transcriptional [1, 2], posttranscriptional [3–5], epigenetic [6–8], and niche [9] regulators have been implicated in HSC maintenance. Among them, a core complex with transcription factors (TFs) TAL1/TCF3/GATA2 and adaptors LMO2/LDB1 lies at the top of the hematopoietic transcriptional hierarchy. TAL1 maintains HSC quiescence and long-term activity through a positive feedback loop involving cytokine receptor c-Kit [10]. It is also essential for myelopoiesis, erythropoiesis, and megakaryopoiesis [11–13]. In lymphoid progenitors, TAL1 is expressed at low levels and its forced expression might lead to leukemia [14]. TCF3 restricts the proliferation of adult HSCs and contributes to their self-renewal activity [15, 16]. Within HSCs, TCF3 primes the expression of lymphoid-associated genes and simultaneously prevents the expression of HSC-associated and nonlymphoid genes [15, 16]. GATA2 is required for HSC survival and limits HSC proliferation [17, 18]. This TF also promotes early stages of lineage differentiation [19]. LMO2 contributes to an HSC-specific transcription program, including c-Kit expression [20]. This adaptor also promotes erythropoiesis [21] and plays an essential role in early T cell development [20]. LDB1 sustains a transcriptional program indispensable for the maintenance of HSCs, including the transcription of genes encoding TAL1, LMO2, and GATA2 [22]. Consequently, *Ldb1* deletion in the mouse results in more severe hematopoietic phenotypes than those observed with other factors in the core complex [22, 23]. The expression of these components can be regulated at multiple levels. Besides transcriptional control [22], the ubiquitination and subsequent degradation of several components have also been reported [24–26]. Despite the above findings, the regulation of the expression of these components remains elusive.

Adaptor RACK1 interacts with multiple TFs and modulates their activation and/or stability. For example, RACK1 binds to STAT1 and STAT3 and promotes their phosphorylation and nuclear translocation in response to type I interferons (IFN-Is) and growth factors, respectively [27, 28]. The interaction between RACK1 and Smad3 hinders Smad3 DNA-binding and thereby modulates TGF-β1 signaling [29, 30]. On the other hand, RACK1 recruits HIF-1α and facilitates its ubiquitination and degradation [31, 32], but its recruitment of Nanog and Pax5 shows opposite effects [33, 34]. Despite these findings, a role of RACK1 in the transcriptional control of HSC fates has not been disclosed.

In this study, we report that RACK1 is expressed across various hematopoietic cell types. It maintains adult and fetal mouse HSCs, at least partially, by directly binding to and stabilizing LDB1. Intriguingly, RACK1 deficiency in hematopoietic cells as well as microenvironmental alterations caused by severe HSC stress and RACK1-deficiency in IFN-I-responsive stromal cells lead to the emergence of stressed HSCs.

## Materials and methods

### Mice

*Rack1*^F/F^ mice on a C57BL/6 background (CD45.2) were described previously [35]. They were crossed with *Mx1-Cre* (CD45.2, Cat# 003556, The Jackson Laboratory, Bar Harbor, ME, USA) [36] and *Vav-iCre* (CD45.2, Cat# C001019, Cyagen Biosciences Inc., Santa Clara, CA, USA) [37] transgenic mice to generate *Mx1-Cre*^+/−^*:Rack1*^F/+^ and *Vav-iCre*^+/−^*:Rack1*^F/+^ mice, respectively. These offspring were then crossed with *Rack1*^F/F^ mice to generate *Mx1-Cre*^−/−^*:Rack1*^F/F^ (referred to as conditional wild type, cWT) and *Mx1-Cre*^+/−^*:Rack1*^F/F^ (referred to as conditional knockout, cKO) mice and *Vav-iCre*^−/−^*:Rack1*^F/F^, *Vav-iCre*^−/−^*:Rack1*^F/+^, *Vav-iCre*^+/−^*:Rack1*^F/+^, and *Vav-iCre*^+/−^*:Rack1*^F/F^ mice, respectively. For transplantation, CD45.2 cKO mice were crossed with B6-CD45.1 mice (Beijing Vital River Laboratory Animal, Inc., Beijing, China, http://www.vitalriver.com.cn). The offspring were subjected to a second crossing to generate CD45.1CD45.2 cWT and CD45.1CD45.2 cKO mice. All mice were maintained under specific pathogen-free conditions 4∼5 animals per cage. Unless otherwise stated, both sexes of 8∼12-week-old mice were used because phenotypes did not show gender dependency. No randomization or blinding was used. Because preliminary experiments indicated dramatic hematopoietic phenotypes of RACK1 deficiency, the sample size was *n* ≥ 5 for each group without transplantation and *n* ≥ 4 for mice after transplantation, respectively. *Mx1-Cre* was induced by single intraperitoneal injection of 10 mg/kg poly(I:C) (Cat# tlrl-pic-5, Invivogen, San Diego, CA, USA).

### Quantitative RT-PCR

The total RNA was extracted by Trizol (Invitrogen, Cambridge, MA, USA) and reverse transcribed with ABScript III RT Master Mix (Abclonal Biotech, Cambridge, MA, USA). Then the synthetic cDNA was examined for the expression of transcription levels of different genes with 2× Universal SYBR Green Fast qPCR Mix (Abclonal Biotech). The Ct value was used to assess the gene expression by the 2^−ΔΔCt^ method. Fold amplification was normalized by *GAPDH*. The following primers were used: human *RACK1*, 5′-CTCTGGGATCTCACAACGG-3′ (forward) and 5′- GCTCTCATCCTGGACAGTG-3′ (reverse); human *LDB1*, 5′-TATCCGCCTACATACCTGGAG-3′ (forward) and 5′-GAAGCCGTTTGTTAAGCTCAAAT-3′ (reverse); and human *GAPDH*, 5′-TGTTGCCATCAATGACCCCTT-3′ (forward) and 5′-CTCCACGACGTACTCAGCG-3′ (reverse).

### Histology

Tissues were removed from mice and fixed in 10% buffered formalin for at least 24 h, dehydrated, and infiltrated with paraffin. Decalcification of bones was carried out in 0.3 M EDTA for 5∼7 days prior to dehydration and embedding. 5 µm paraffin sections were then prepared and stained with hematoxylin and eosin for brightfield microscopy.

### Immunohistochemistry (IHC)

Formalin-fixed and paraffin-embedded tissues were subjected to fluorescent multiplex IHC. pH 9.0 EDTA heat antigen retrieval was employed. An Opal 7-Color Manual IHC kit (Cat# NEL811001KT) was ordered from PerkinElmer (Akron, OH, USA). Antibody against RACK1 (Cat# 610178) was purchased from BD Bioscience (Eugene, OR, USA). Antibody against c-Kit (Clone: 2B8) was from BioLegend (San Diego, CA, USA).

### Magnetic cell separation (MACS)

Bone marrow (BM) cells were stained with PE-conjugated antibody against c-Kit in PBS containing 2% fetal bovine serum (FBS) for 30 min on ice in the dark. Samples were washed once in staining buffer. Then, the cells were incubated with anti-PE microbeads (Cat# 130-048-801, Miltenyi Biotec, Bergisch Gladbach, Germany), and all c-Kit^+^ cells were enriched using an MS column (Cat# 130-042-201, Miltenyi Biotec). The purity of c-Kit^+^ cells was > 90% as confirmed by flow cytometry. Human umbilical cord blood samples were collected from healthy donors with informed consent at public hospitals in Beijing, China. Mononuclear cells were isolated via density gradient centrifugation using Ficoll-Paque Plus (Cat# 17-1440-02, GE HealthCare, Chicago, IL, USA). Further enrichment for CD34^+^ cells was performed using a human CD34 MicroBead Kit (Cat# 130-046-702, Miltenyi Biotec) and an MS column according to the manufacturer’s protocol. The purity of CD34^+^ cells was > 80% as confirmed by flow cytometry.

### Immunoblotting (IB) analysis

Cells were lysed in ice-cold RIPA buffer supplemented with protease/phosphatase inhibitors. Equal amounts of protein were separated with polyacrylamide gels, electrotransferred onto polyvinylidene difluoride membranes (Cat# IPVH00010, Merck Millipore, Burlington, VT, USA), and blotted with appropriate primary antibodies at 4 °C overnight, followed by incubation with corresponding Horseradish peroxidase-conjugated secondary antibodies at room temperature for 1 h. Immunoreactive bands were visualized by an ECL Chemiluminescence Kit (Cat# SQ202, EpiZyme, Shanghai, China). Antibody against RACK1 (Cat# 610178) was purchased from BD Bioscience. Antibodies against β-actin (Cat# sc-8432) and HA (Cat# sc-805) were ordered from Santa Cruz Biotechnology (Santa Cruz, CA, USA). Antibodies against Myc tag (Cat# M192-3) and GFP (Cat# 598) were from MBL International (Woburn, MA, USA). Antibodies against LDB1 (Cat# A17580) and LMO2 (Cat# A1903) were from Abclonal Biotech. Antibodies against GATA2 (Cat# 11103-1-AP), TAL1 (Cat# 55317-1-AP), TCF3 (Cat# 14519-1-AP), GST (Cat# 66001-2-Ig), and Ubiquitin (Cat# 10201-2-AP) were from Proteintech (Chicago, IL, USA).

### Flow cytometry and fluorescence-activated cell sorting (FACS) sorting

The blood was collected with 0.025% heparin sodium via retroorbital bleeding. BM cells were flushed out of the BM cavity of both femurs using a 5 mL syringe with ice-cold PBS, followed by filtration through a 70-μm strainer. Splenocytes and fetal liver cells were directly mechanically extracted by mashing on 70-μm strainers. Red blood cells were depleted in a hypotonic solution. Single-cell suspensions were stained with fluorescence-conjugated surface marker antibodies from BioLegend in PBS containing 0.1% sodium azide and 2% FBS for 30 min on ice in the dark. For LDB1 staining, LSKs were fixed/permeabilized with a Foxp3/Transcription Factor Staining Buffer Set (Cat# 00-5523-00, eBioscience, San Diego, CA, USA), followed by sequential staining with an antibody against LDB1 (Cat# ab318948, Abcam, Cambridge, UK) and an AF647-conjugated donkey anti-rabbit second antibody (Cat# 150075, Abcam) in permeabilization buffer for 1 h and 30 min at 4 °C, respectively. Samples were washed once in staining buffer. An isotype antibody was included as a negative control. Antibodies against the following surface markers were used: CD3e (Clone: 145-2C11), B220 (Clone: RA3-6B2), CD11b (Clone: M1/70), Gr-1 (Clone: RB6-8C5), Ter119 (Clone: TER-119), c-Kit (Clone: 2B8), Sca-1 (Clone: D7), CD150 (Clone: TC15-12F12.2), CD48 (Clone: HM48-1), CD127 (Clone: A7R34), CD135 (Clone: A2F10), CD16/32 (FcgRII/III) (Clone: 93), CD45.1 (Clone: A20), and CD45.2 (Clone: 104). The mouse lineage cocktail contains anti-CD3e, B220, CD11b, Gr-1, and Ter119 antibodies. For blood samples, an anti-CD16/32 antibody was used to block the Fc receptor. All flow cytometric data were acquired using a Becton Dickinson FACS Fortessa machine (East Rutherford, NJ, USA). For cell sorting, surface staining was performed in PBS containing 2% FBS without NaN_3_. Immediately before sorting, cells were incubated with 4′,6-diamidine-2-phenylindole (DAPI, 0.2 μg/mL) for discrimination of dead cells. Sorting was conducted with a Becton Dickinson FACSAria II sorter. Data were analyzed with FlowJo10.0 (TreeStar, San Carlos, CA, USA) software.

### PCR analysis of recombination efficiency

Recombination efficiency of the *Rack1* allele was performed as previously described [34]. In brief, DAPI^−^Lin^−/low^ Sca-1^+^c-Kit^+^ LSKs were sorted and subjected to genomic DNA isolation, followed by PCR. After 2% agarose gel electrophoresis, PCR products were visualized on a UV transilluminator. The recombination efficiency was calculated via densitometric readings: (*Rack1*-deleted × 100%)/(*Rack1*-deleted + *Rack1*-floxed).

### Isolation of BM stromal cells

Both femurs were cut into small pieces with sterile scissors and mechanically dissociated using a pestle in ice-cold RPMI-1640, followed by digestion with 2 mg/mL of type I collagenase (Cat# C0130, Sigma-Aldrich, St. Louis, MO, USA) and 4 mg/mL dispase II (Cat# D4693, Sigma-Aldrich) in PBS for 45 min at 37 °C with gentle shaking. After being filtered through a 70-μm cell strainer, cells were subjected to centrifugation at 420 × *g*, 4 °C for 5 min. After washing in PBS with 2% FBS, single-cell suspensions were stained with fluorescence-conjugated antibodies against CD45 and Ter119, followed by FACS sorting of CD45^−^DAPI^−^Ter119^−^ stromal singlets.

### Transplantation

To evaluate niche-specific effects, 2 × 10^6^ BM cells from CD45.1 WT mice were injected into the tail vein of a CD45.2 cWT or a CD45.2 cKO littermate after 9 Gy radiation. For chimeric BM transplantation to analyze adulthood *Rack1* deletion in hematopoietic cells, 2 × 10^6^ BM cells from CD45.1CD45.2 cWT and CD45.2 cKO mice were co-transferred into a CD45.1 WT recipient after 9 Gy radiation. For non-chimeric BM transplantation to analyze adulthood *Rack1* deletion in hematopoietic cells, 2 × 10^6^ BM cells from a CD45.1CD45.2 cWT mouse or a CD45.1CD45.2 cKO littermate were transplanted into a CD45.2 WT recipient after 9 Gy radiation. For fetal liver cell transplantation, 1 × 10^6^ fetal liver cells from a CD45.2 embryo were transferred into a CD45.1 WT recipient after 8 or 9 Gy radiation. To analyze the role of LDB1, 1000 transduced CD45.2 LSKs along with 5 × 10^5^ BM cells from CD45.1CD45.2 WT mice were co-transferred into a CD45.1 WT recipient after 9 Gy radiation. The congenic markers CD45.1 and CD45.2 were used for distinguishing cells from the different donors and recipients.

### Apoptosis analysis

Single-cell suspensions were stained with fluorescence-conjugated antibodies against surface markers and Annexin V-FITC (Cat# 640906, BioLegend) or Annexin V-APC (Cat# 640920, BioLegend) resuspended in 300 μL binding buffer containing calcium ions for 30 min on ice in the dark. Samples were washed once in binding buffer. Apoptosis was immediately assessed by flow cytometric analysis of Annexin-V staining.

### 5-bromo-2′-deoxyuridine (BrdU) incorporation

Mice were injected intraperitoneally with 1 mg BrdU (Cat# B9285, Sigma-Aldrich) diluted in 0.1 mL sterile PBS and were sacrificed 24 h later. Single-cell suspensions were stained with fluorescence-conjugated antibodies against surface markers followed by detection of BrdU incorporation with a BD Pharmingen^TM^ BrdU Flow Kit (Cat# 559619, Becton-Dickinson). Briefly, cells were fixed and permeabilized with BD Cytofix/Cytoperm Buffer and BD Cytoperm Permeabilization Buffer Plus, treated with 300 μg/mL DNase I, and then stained with FITC-conjugated anti-BrdU.

### Single-cell RNA sequencing (scRNA-seq) library construction

For droplet-based scRNA-seq (10× Genomics), DAPI^−^Lin^−/low^c-Kit^+^ singlets from the same genotype mice (*n* = 4 mice/sample) were sorted and pooled together. Libraries were produced with a Chromium system (10× Genomics, PN120263) following the manufacturer’s instructions (https://support.10xgenomics.com/single-cell-gene-expression/library-prep/doc/technical-note-assay-scheme-and-configuration-of-chromium-single-cell-3-v2-libraries) and sequenced on the Illumina HiSeq X Ten platform in 150 bp paired-end manner (sequenced by Novogene Genomics).

### Processing of scRNA-seq data

Sequencing data from 10× Genomics was processed with Cell Ranger software (version 6.1.0) with default mapping arguments. Data were mapped to the mm10 reference data (version 3.0.1) downloaded from 10× Genomics official website. The UMI data matrixes of quantitative gene expression for each sample were obtained. Expression levels were normalized by log (TPM/100 + 1), since the unique molecular identifier (UMI) number of most of our samples sequenced by using 10× Genomics method was on the order of 10,000 transcripts.

### Quality control of 10× datasets

The quality control was performed to filter low-quality cells. For each dataset, cells with > 500 genes and < 7,500 genes expressed were retained. In addition, cells for which 10% of reads mapped to mitochondrial genes were excluded. We used DoubletFinder (version 2.0.3, https://doi.org/10.1016/j.cels.2019.03.003) for the elimination of influence from potential cell doublets. For samples collected 12 days after poly(I:C) injection, 1,846 and 2,407 cells in the cWT and cKO datasets were removed, respectively. For samples collected 6 weeks after poly(I:C) injection, 1471 and 675 cells in the cWT and cKO datasets were removed, respectively.

### Integrated analysis of data from different genotypes, dimensionality reduction, and clustering

For droplet-based 10× Genomics data, scRNA-seq analysis was conducted following the Seurat workflow (https://satijalab.org/seurat/)(version 4.0.2) [38]. The mutual nearest neighbor algorithm (MNN) was applied to the pooled raw data from cWT and cKO mice via the fastMNN function implemented in the SeuratWrappers package (version 0.3.1) to integrate datasets derived from different genotypes. Specifically, SCTransform normalization was implemented separately for each dataset, in which the top 2000 feature genes were selected and used for data integration. To eliminate cell cycle effects, we used the CellCycleScoring function in Seurat to calculate the cell cycle phase scores with cell cycle-related genes, including a previously defined core set of 43 G1/S and 54 G2/M genes [39, 40]. Then, genes highly correlated with cell cycle scores (absolute correlation coefficient > 0.3) were excluded from highly variable genes. Highly variable genes after excluding putative cell cycle genes were used for the integration procedure. Then, the top 20 MNN dimensions were used to perform dimensionality reduction by uniform manifold approximation and projection (UMAP). Unsupervised clustering was performed by the FindClusters function at the resolution of 1.

### Differential expression analysis

We used the FindAllMarkers function and FindMarkers function in Seurat to identify cluster feature genes between multiple clusters and differentially expressed genes (DEGs) between two given clusters, respectively. The log(TPM/100 + 1) transformed expression values and “wilcox” method were used. Only genes that are detected in a minimum fraction of 0.1 cells in either of two populations were included for testing. Genes with fold-change ≥ 1.5 and *P* value ≤ 0.05 were retained as DEGs.

### Gene ontology and gene sets score analysis

Gene Ontology biological process (GO_BP) enrichment analysis was performed using clusterProfiler (version 4.0.5) [41]. Gene sets, including proliferation signature (GO:0008283), myeloid (GO:0002573), megakaryocytic (GO:0030219), erythroid (GO:0030218) and lymphoid (GO:0030098), were obtained from Molecular Signatures Database [GSEA | MSigDB (gsea-msigdb.org)].

### Trajectory analysis

The Monocle3 package (version 1.3.7) was used to predict developmental trajectories in the major hematopoietic clusters [42]. The UMAP embedding was input, and the “learn_graph” function was applied to infer the trajectories. The “order_cells” function was then used to select the transcriptional HSC cluster as the root (starting point), thereby inferring the pseudotime value of each cell. The pseudotime trajectory was subsequently visualized using the “plot_cells” function. In addition, the “DiffusionMap” function from the destiny package (version 3.18.0) in R was employed to predict developmental trajectories in the major hematopoietic clusters with default settings [43].

### Cell culture, transduction, and transfection

CD34^+^ human umbilical cord cells were cultured in StemSpan^TM^ SFEM II Medium containing 1× StemSpan^TM^ Human CD34^+^ Cell Expansion Supplement (Cat# 09605 and 02691, respectively, STEMCELL Technologies, Vancouver, Canada). Human T-cell acute lymphoblastic leukemia (T-ALL) cell lines Jurkat, CEM, Molt3, and HSB2 have been described previously [44] and were cultured in RPMI-1640 supplemented with 10% FBS, 100 U/mL penicillin, and 100 μg/mL streptomycin. HEK-293T cells were purchased from the Shanghai Institutes for Biological Sciences and were cultured in DMEM supplemented with 10% FBS, 100 U/mL penicillin, and 100 µg/mL streptomycin. All cell cultures were maintained at 37 °C with 5% carbon dioxide. Hoechst staining was performed quarterly for the detection of mycoplasma contamination, and no extra-nuclear staining was found. Mammalian or prokaryotic expression vectors used in this study were generated by cloning PCR-amplified products into pcDNA3.1 (+) vector, pEGFP-N1 vector, and pGEX-KG vector, respectively, and confirmed by DNA sequencing. Human RACK1 small interfering RNA (siRNA, Cat# 4392420) was ordered from Invitrogen. Transfection was performed with Lipofectamine 2000 (Cat# 11668019, Invitrogen) according to the manufacturer’s protocol.

Lentivirus-based human RACK1 short hairpin RNAs (shRNAs, GATGTGGTTATCTCCTCAGAT and CAAGCTGAAGACCAACCACAT) and the non-targeting control shRNA were from Shanghai GeneChem (Shanghai, China). Lentivirus-based mouse LDB1 (NM_001113408.3) expression vector and the mock control (GV492) were also obtained from Shanghai GeneChem. Lentiviral transduction (multiplicity of infection = 100) was performed according to the manufacturer’s protocols. In particular, live LSKs (4 × 10^4^/sample) were sorted 5 days after Cre induction and resuspended in 200 μL StemSpan^TM^ SFEM Medium (Cat# 09650, STEMCELL Technologies, Vancouver, Canada) containing 100 U/mL penicillin, 100 μg/mL streptomycin, 100 ng/mL mSCF (Cat# 250-03, PeproTech, Cranbury, NJ, USA), 25 ng/mL mFLT-3L (Cat# 250-31L, PeproTech), 25 ng/mL TPO (Cat# 315-14, PeproTech), 10 ng/mL IL-6 (Cat# 216-16, PeproTech), and 10 ng/mL IL-3 (Cat# 213-13-10UG, PeproTech). Then, lentivirus was added with 2 μg/mL polybrene (Cat# TR-1003, Sigma-Aldrich). The infection mixture was centrifuged for 3 h at 2000 rpm, 33 °C, followed by incubation at 37 °C for 96 h.

### Colony-forming ability assays

The soft agar cloning assays for T-ALL cells were performed as described previously [44]. T-ALL cells were seeded into 6-well plates at a density of 3 × 10^3^ per well. On day 28, MTT incorporation was included to make the colonies visible. Colony-forming unit (CFU) assays for CD34^+^ human cord blood cells were performed with MethoCult™ H4435 Enriched (Cat# 04435, STEMCELL Technologies) at a density of 2 × 10^3^ per well in 6-well plates. Typical colonies were photographed and counted using an inverted wide-field microscope (Cat# CKX53, Olympus, Shinjuku, Japan).

### Glutathione S-transferase (GST) pull-down assays

GST and GST-RACK1 were expressed and purified with glutathione-Sepharose beads (Cat# 17-0756-01, Amersham Pharmacia, Uppsala, Sweden), according to the manufacturer’s instruction. Lysates of HEK-293T cells expressing Myc-tagged TAL1, LMO2, LDB1, TCF3, or GATA2 were incubated with GST or GST-RACK1 bound to glutathione-Sepharose beads in NETN buffer (100 mM NaCl, 20 mM Tris-Cl, pH 8.0, 0.5 mM EDTA, 0.5% NP-40, supplemented with protease/phosphatase inhibitors). The adsorbed proteins were washed with NETN buffer five times, followed by IB analysis.

### Co-immunoprecipitation (co-IP)

Cells were solubilized in IP lysis buffer (10 mM Tris-Cl, pH 7.5, 2 mM EDTA, 1% NP-40, 150 mM NaCl, supplemented with protease/phosphatase inhibitors). After the protein concentration was determined by the Bradford assay, appropriate amounts (0.5–1.5 mg) of protein were used for IP with 30 μL protein A/G beads (50% slurry, Cat# sc-2003, Santa Cruz Biotechnology) and 1 μg antibody. Immunoprecipitated proteins were washed with washing buffer (10 mM Tris-Cl, pH 7.5, 2 mM EDTA, 1% NP-40, 500 mM NaCl, supplemented with PMSF) five times, followed by IB analysis.

### In vivo ubiquitination assay with IP

In vivo ubiquitination assay with IP was performed as previously described [34]. In brief, cells were solubilized under partially denaturing conditions. Genomic DNA was disrupted with sonication, followed by IP and IB.

### Statistical analysis

Statistical analysis of scRNA-seq data was conducted in R language. Two-sample Wilcoxon Rank Sum test was employed for comparisons of gene numbers, transcript counts, gene expression levels, and feature scores between two given clusters of cells. More detailed information of statistical analysis is described above. Statistical analysis other than scRNA-seq were analyzed using Prism 6.0 (GraphPad). All samples represent biological replicates. Two-tailed Student’s *t* test or one-way ANOVA was used to evaluate quantitative variables that passed the normality test (Shapiro-Wilk test) and homogeneity of variance test (Levene’s test). Wilcoxon rank-sum test, Welch ANOVA, or Kruskal–Wallis test was used to evaluate quantitative data that failed to pass the normality test or homogeneity of variance test. *P* < 0.05 was considered significant. The exact sample size (*n*), how many times the experiment shown was replicated, and definitions of statistical methods and measures are provided in figure legends.

## Results

### RACK1 is expressed across various hematopoietic cell types

**Fig. 1:**
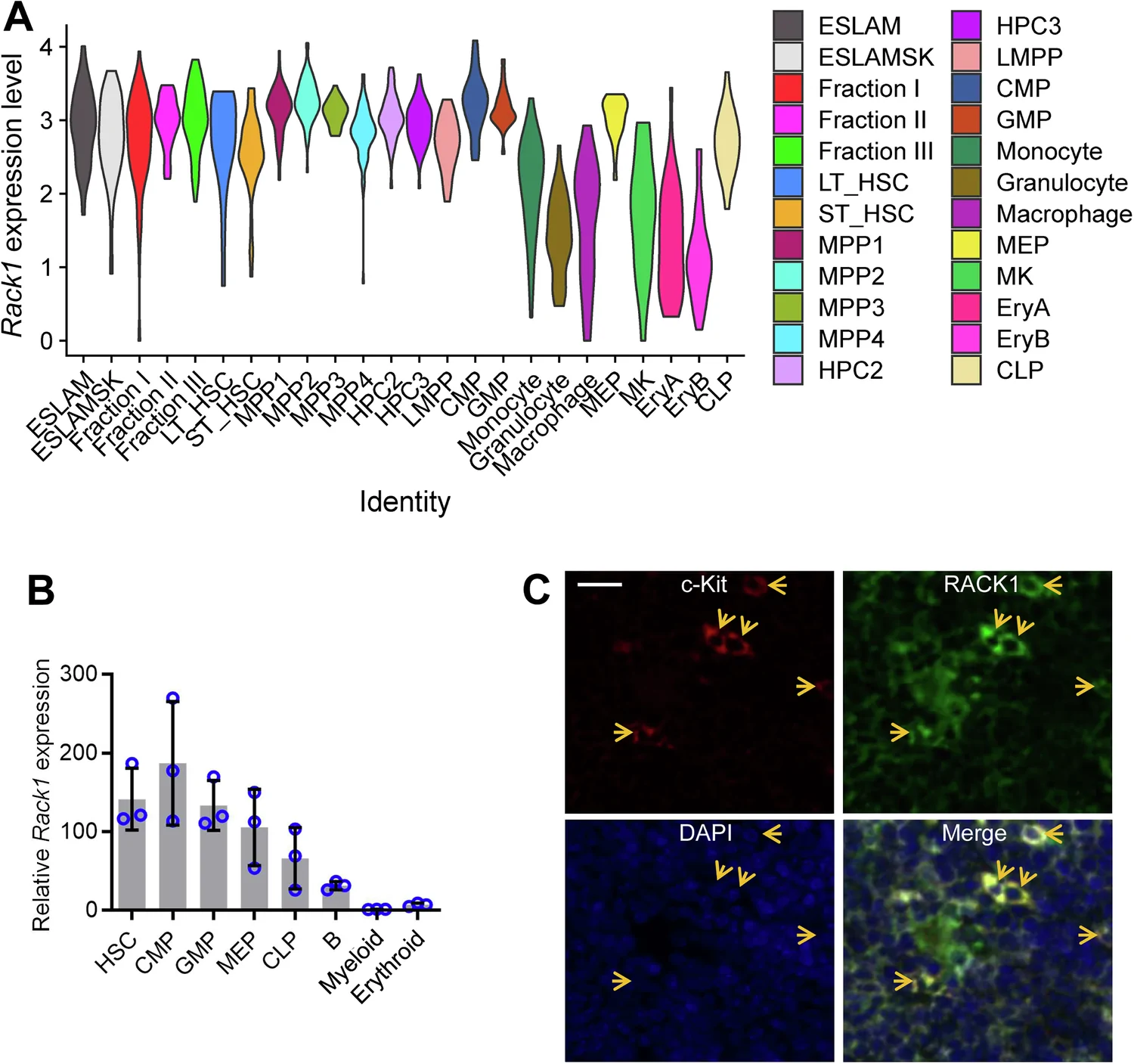
RACK1 is expressed across various hematopoietic cell types. **A** *Rack1* expression in BM hematopoietic populations of adult mice as referred from reported scRNA-seq data [45]. Hematopoietic stem cell (HSC) populations: LT-HSC (long-term HSC), Fraction I, Fraction III, ESLAM (EPCR^+^CD150^+^CD48^−^CD45^+^), and ESLAMSK (Sca-1^+^c-Kit^+^ESLAM). Multipotent progenitor (MPP) populations: ST-HSC (short-term HSC), LMPP (CD34^+^CD135^+^LSK), MPP1, MPP2, MPP3, MPP4, Fraction II, HPC2 (hematopoietic progenitor cell 2), and HPC3. Committed progenitor populations: common myeloid progenitor (CMP), granulocyte-monocyte progenitor (GMP), megakaryocyte-erythrocyte progenitor (MEP), and common lymphoid progenitor (CLP). Mature myeloid cell populations: granulocyte, monocyte, and macrophage. Other populations: erythroid population A and B (EryA and EryB), and megakaryocyte (Mk). **B** Quantitative RT-PCR analysis of *Rack1* expression in the indicated hematopoietic subsets sorted from the BM of adult mice (*n* = 3 mice). Error bars show mean ± SD. Data are representative of two independent experiments. **C** Fluorescent multiplex immunohistochemistry with tyramide signal amplification was performed to analyze the expression of RACK1 and c-Kit in the BM of adult mice. DAPI, 4′,6-diamidino-2-phenylindole. Scale bar: 20 μm. Data are representative of three independent experiments.

Searching the scRNA-seq data in a previous report [45] revealed high *Rack1* expression in BM immunological and transcriptional hematopoietic stem and progenitor cells (HSPCs) as well as lineage-committed progenitors of adult mice, as compared with monocytes, granulocytes, macrophages, megakaryocytes, and erythroid cells (Fig. 1A). Then, live Lin^−/low^Sca-1^+^c-Kit^+^CD150^+^CD48^−^ HSCs, Lin^−/low^Sca-1^−^c-Kit^+^CD34^+^CD16/32^-^ common myeloid progenitors (CMPs), Lin^−/low^Sca-1^−^c-Kit^+^CD34^+^CD16/32^+^ granulocyte-monocyte progenitors (GMPs), Lin^−/low^Sca-1^−^c-Kit^+^CD34^-^CD16/32^−^ megakaryocyte-erythroid progenitors (MEPs), Lin^−/low^Sca-1^low^c-Kit^low^CD127^+^CD135^+^ common lymphoid progenitors (CLPs), CD19^+^CD11b^−^Ter119^−^ B cells, CD19^−^CD11b^+^Ter119^−^ myeloid cells, and CD19^−^CD11b^−^Ter119^+^ erythroid cells were sorted from the BM of adult mice. Quantitative RT-PCR revealed a similar tendency to the reported scRNA-seq data (Fig. 1B). Furthermore, fluorescent multiplex IHC with antibodies against c-Kit and RACK1 demonstrated ubiquitous RACK1 expression in the BM of adult mice with enrichment in c-Kit^+^ hematopoietic cells (Fig. 1C).

### Adulthood *Rack1* deletion in IFN-I-responsive cells results in rapid and profound hematopoietic failure

**Fig. 2:**
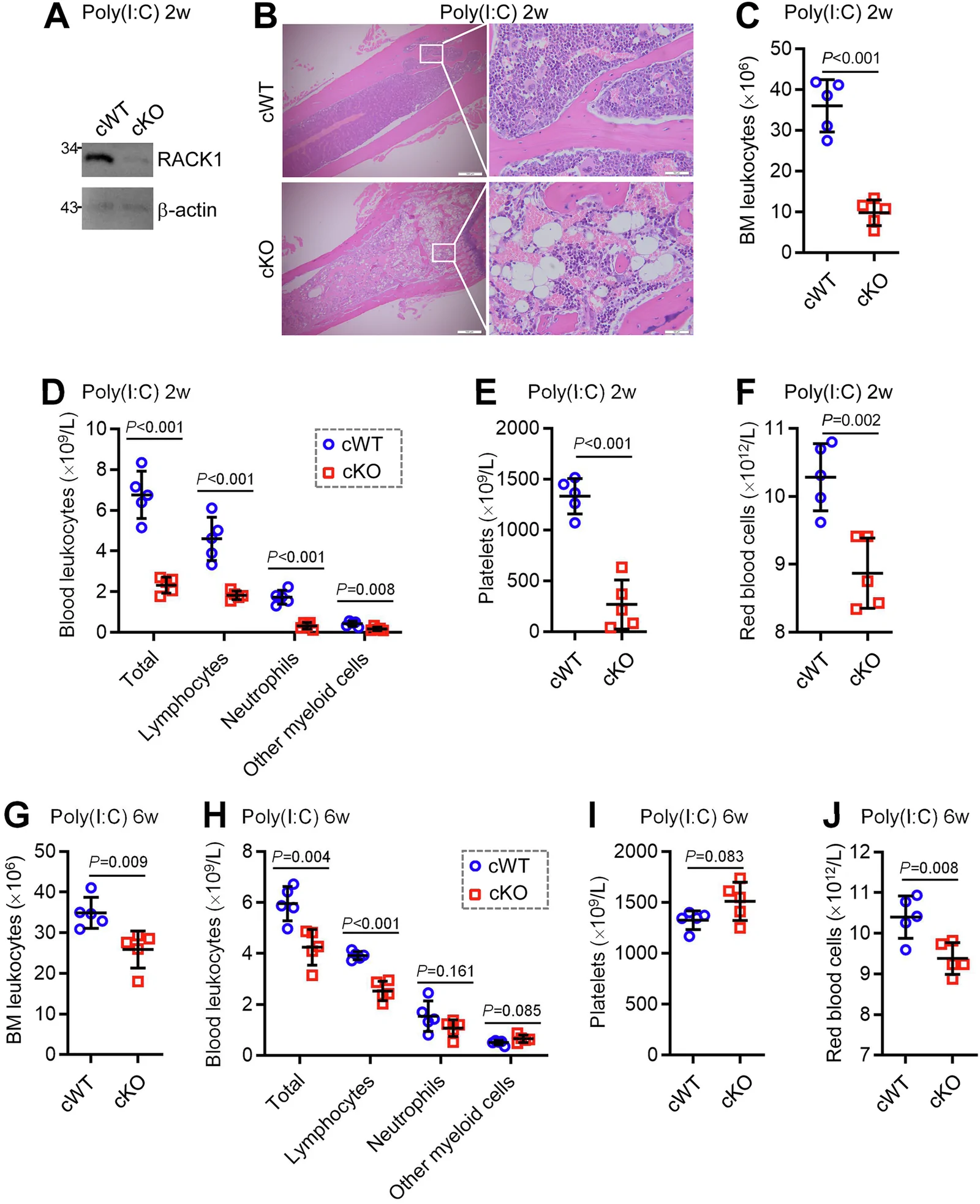
Adulthood *Rack1* deletion in IFN-I-responsive cells results in rapid and profound hematopoietic failure. Two (**A**−**F**) or six (**G**−**J**) weeks after intraperitoneal injection with 10 mg/kg poly(I:C), adult *Mx1-Cre*^−/−^*:Rack1*^F/F^ (cWT) and *Mx1-Cre*^+/−^*:Rack1*^F/F^ (cKO) mice (*n* = 5 mice/group) were subjected to the following assays. **A** Immunoblotting (IB) analysis of RACK1 expression in MACS-enriched c-Kit^+^ BM cells. **B** Hematoxylin and eosin staining of transversal sections of the femurs. Scale bar: 500 μm (left) or 50 μm (right). **C**, **G** The number of total BM cells in the femurs. **D**, **H** The numbers of blood total leukocytes, lymphocytes, neutrophils, and other myeloid cells. **E**, **I** The number of platelets. **F**, **J** The number of red blood cells. Error bars show mean ± SD. *P* values are shown above with two-tailed unpaired Student’s *t* test. Data are representative of three independent experiments.

To study whether RACK1 has a role in hematopoiesis, *Mx1-Cre*^−/−^*:Rack1*^F/F^ (cWT) and *Mx1-Cre*^+/−^*:Rack1*^F/F^ (cKO) mice were generated. In this model, the injection of poly(I:C) induces the deletion of the targeted gene in IFN-I-responsive cells, including hematopoietic cells [36]. Two weeks after treatment of adult cKO mice and their littermates with a single injection of 10 mg/kg poly(I:C), IB revealed the loss of RACK1 in MACS-enriched BM c-Kit^+^ hematopoietic cells from cKO mice, as compared with cWT littermates (Fig. 2A). Hematoxylin and eosin staining revealed that the bone cavities of cKO mice were devoid of hematopoietic cells (Fig. 2B), with less than one third of the BM cellularity in cWT littermates (Fig. 2C). Accordingly, the numbers of blood total leukocytes, lymphocytes, neutrophils, and platelets in cKO mice all dropped to less than one third of their counterparts in cWT littermates (Fig. 2D, E). Furthermore, cKO mice showed fewer other myeloid cells and red blood cells than cWT mice (Fig. 2D, F). Despite that, cKO mice looked as normal as their littermates during the next several months observed. Therefore, we tracked the BM and blood cellularity over time. Six weeks after poly(I:C) injection, the BM cellularity in cKO mice returned to more than two-thirds of that in cWT littermates (Fig. 2G). In the blood, the numbers of total leukocytes and lymphocytes also returned to around two-thirds of their counterparts in cWT littermates (Fig. 2H). The numbers of neutrophils and other myeloid cells in cKO mice were largely comparable to their counterparts in cWT littermates (Fig. 2H). Furthermore, cKO mice showed a tendency of more platelets than their littermates (Fig. 2I). There was also some recovery of red blood cells in cKO mice (Fig. 2J). Collectively, these results show that adulthood *Rack1* deletion in IFN-I-responsive cells results in rapid hematopoietic failure, but recovery occurs soon after.

### Adulthood *Rack1* deletion in IFN-I-responsive cells leads to rapid and profound loss of HSCs

**Fig. 3:**
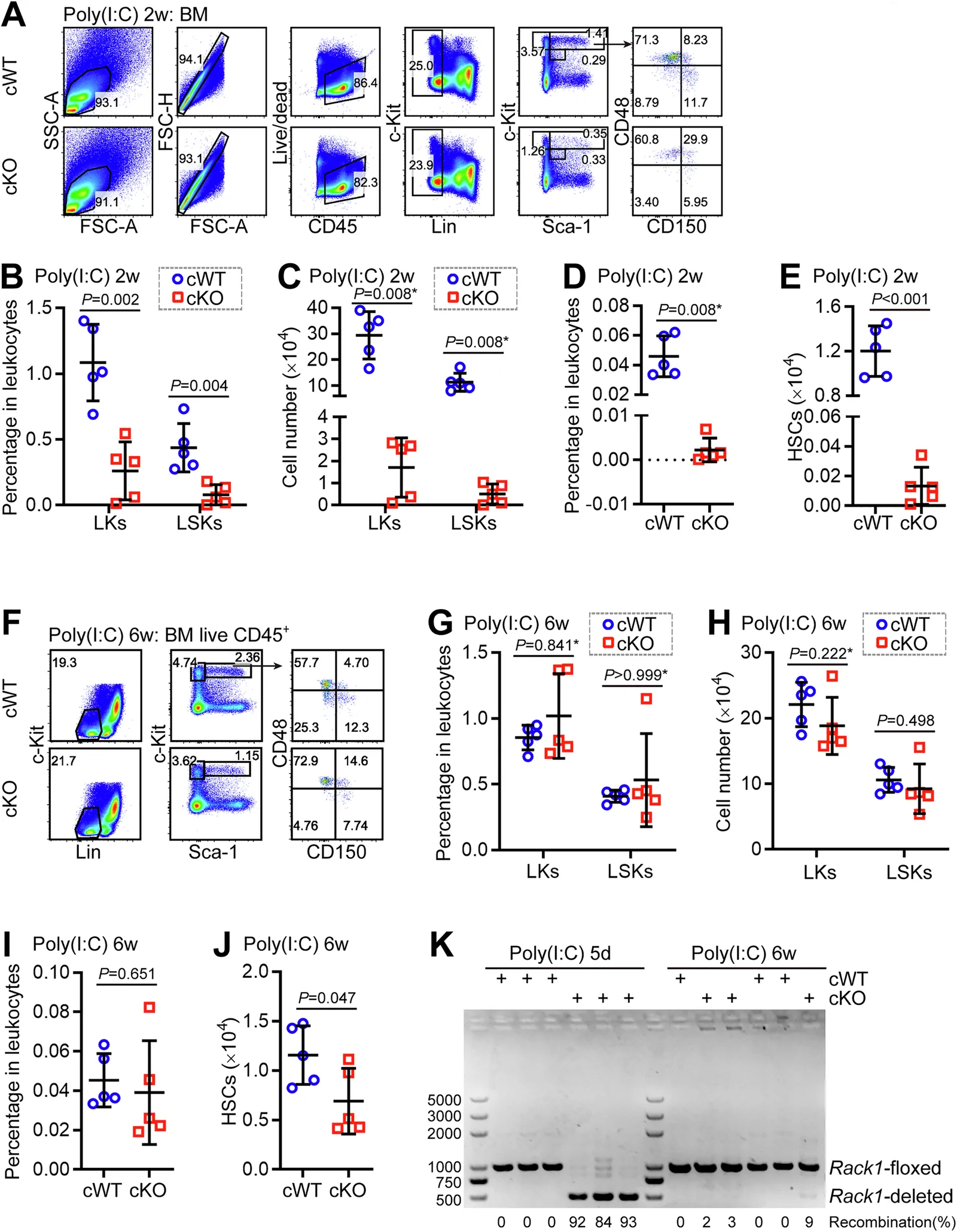
Adulthood *Rack1* deletion in IFN-I-responsive cells leads to rapid and profound loss of HSCs. Two (**A**−**E**) or six (**F**−**J**) weeks after poly(I:C) injection, adult cWT and cKO mice (*n* = 5 mice/group) were subjected to the following assays. **A**, **F** Representative gating strategy for flow cytometry analysis of LKs, LSKs, and HSCs in the BM. **B**, **G** The frequencies of LKs and LSKs. **C**, **H** The numbers of LKs and LSKs. **D**, **I** The frequency of HSCs. **E**, **J** The number of HSCs. **K** Recombination efficiency of the conditional *Rack1* allele in live LSKs sorted from adult cWT and cKO mice (*n* = 3 mice/group) 5 days or 6 weeks after poly(I:C) injection. Error bars show mean ± SD. *P* values are shown above with two-tailed unpaired Student’s *t* test or Wilcoxon rank-sum test (indicated with the symbol ‘*’). Data are representative of three independent experiments.

At the HSPC level, flow cytometry analysis indicated that both Lin^−/low^Sca-1^−^c-Kit^+^ LK and Lin^−/low^Sca-1^+^c-Kit^+^ LSK compartments were barely recognizable in cKO BM cells 2 weeks after Cre induction (Fig. 3A). Accordingly, their frequencies and numbers were much lower in cKO mice than those in cWT mice (Fig. 3A−C). Furthermore, the frequency and number of HSCs significantly decreased (Fig. 3A, D, E). As compared with LKs in cWT mice, LKs in cKO mice displayed a similar MEP proportion, a lower CMP proportion, and a tendency of higher GMP proportion (Fig. S1A, B). Despite that, the numbers of GMPs, CMPs, and MEPs in cKO mice were all significantly lower than those in cWT mice (Fig. S1B) because of the dramatic reduction of LKs (Fig. 3C). As for CLPs, their frequency and number were also lower in cKO mice than those in cWT mice (Fig. S1C, D). In line with the prompt recovery of BM cellularity (Fig. 2G) and various lineages of blood cells (Fig. 2H−J), cKO mice showed comparable frequencies and numbers of LKs and LSKs to their littermates 6 weeks after poly(I:C) injection (Fig. 3F−H). The frequency of HSCs was largely comparable between the two genotypes, although their number in cKO mice was still lower than that in cWT mice (Fig. 3F, I, J).

Cre-loxp system-driven recombination may not be as efficient as expected. It is possible that some HSPCs in cKO mice escape recombination after poly(I:C) injection. They might expand thereafter and mediate the apparent hematopoietic recovery. In line with this notion, PCR analysis revealed around 90% recombination efficiency of the *Rack1* allele in live LSKs sorted from cKO mice 5 days after poly(I:C) injection. However, the efficiency dropped to < 10% 6 weeks after poly(I:C) injection (Fig. 3K).

### Rapid HSC exhaustion upon adulthood *Rack1* deletion results from cell-intrinsic defects

The *Mx1-Cre* transgene may induce significant deletion outside the hematopoietic system, including the BM stromal compartment and the liver [36]. Indeed, IB revealed the reduction of RACK1 in live CD45^−^Ter119^−^BM stromal cells sorted from cKO mice 2 weeks after Cre induction, as compared to those from cWT mice (Fig. S2A). To rule out a possible contribution to the observed phenotypes from cKO BM stromal cells, BM cells from CD45.1 WT mice were transplanted into lethally irradiated CD45.2 cWT mice (WT→cWT) and CD45.2 cKO mice (WT→cKO). After 8 weeks of BM reconstitution, these recipients were treated with a single injection of 10 mg/kg poly(I:C). The BM cellularity in these two groups of recipients was comparable 2 weeks after poly(I:C) injection (Fig. S2B). The frequencies and numbers of LKs and HSCs were also comparable (Fig. S2C−G), although WT→cKO recipients exhibited a tendency for reduced LSKs than WT→cWT recipients (Fig. S2C−E). Thus, niche-specific effects are not a major contributor to the rapid hematopoietic failure.

On the other hand, BM cells from CD45.1CD45.2 cWT mice (cWT→WT) and CD45.1CD45.2 cKO mice (cKO→WT) were separately transplanted into lethally irradiated CD45.2 WT recipients. After 8 weeks of BM reconstitution, over 90% of blood leukocytes in these recipients came from donor cells (Fig. S3A, B). The numbers of blood leukocytes, platelets, and red blood cells were comparable between these two groups of recipients (Fig. S3C−E). Two weeks after poly(I:C) injection, over 80% of leukocytes in the blood of these two groups were still donor-derived, even though the frequency of donor-derived leukocytes was lower in cKO→WT recipients than cWT→WT recipients (Fig. S3F, G). Flow cytometry revealed that the numbers of donor-derived blood total leukocytes and myeloid cells in cKO→WT recipients dropped to about one third of their counterparts in cWT→WT recipients (Fig. S3F, H). Notably, the numbers of donor-derived blood B cells and CD45^+^ erythroid cells in cKO→WT recipients dropped even more profoundly (Fig. S3F, H), partially in line with our previous finding that RACK1 is essential for B-cell development [34]. However, the number of donor-derived blood T cells was only slightly reduced in cKO→WT recipients (Fig. S3F, H). In the BM, about 99% and 97% of leukocytes were donor-derived in cWT→WT and cKO→WT recipients, respectively (Fig. S3I, J). The frequencies and numbers of donor-derived LKs and LSKs were much lower in cKO→WT recipients than those in cWT→WT mice (Fig. S3I, K, L). Furthermore, the frequency and number of donor-derived HSCs in cKO→WT recipients were also significantly lower (Fig. S3I, M, N). These data suggest that rapid HSC exhaustion upon adulthood *Rack1* deletion results from cell-intrinsic defects.

**Fig. 4:**
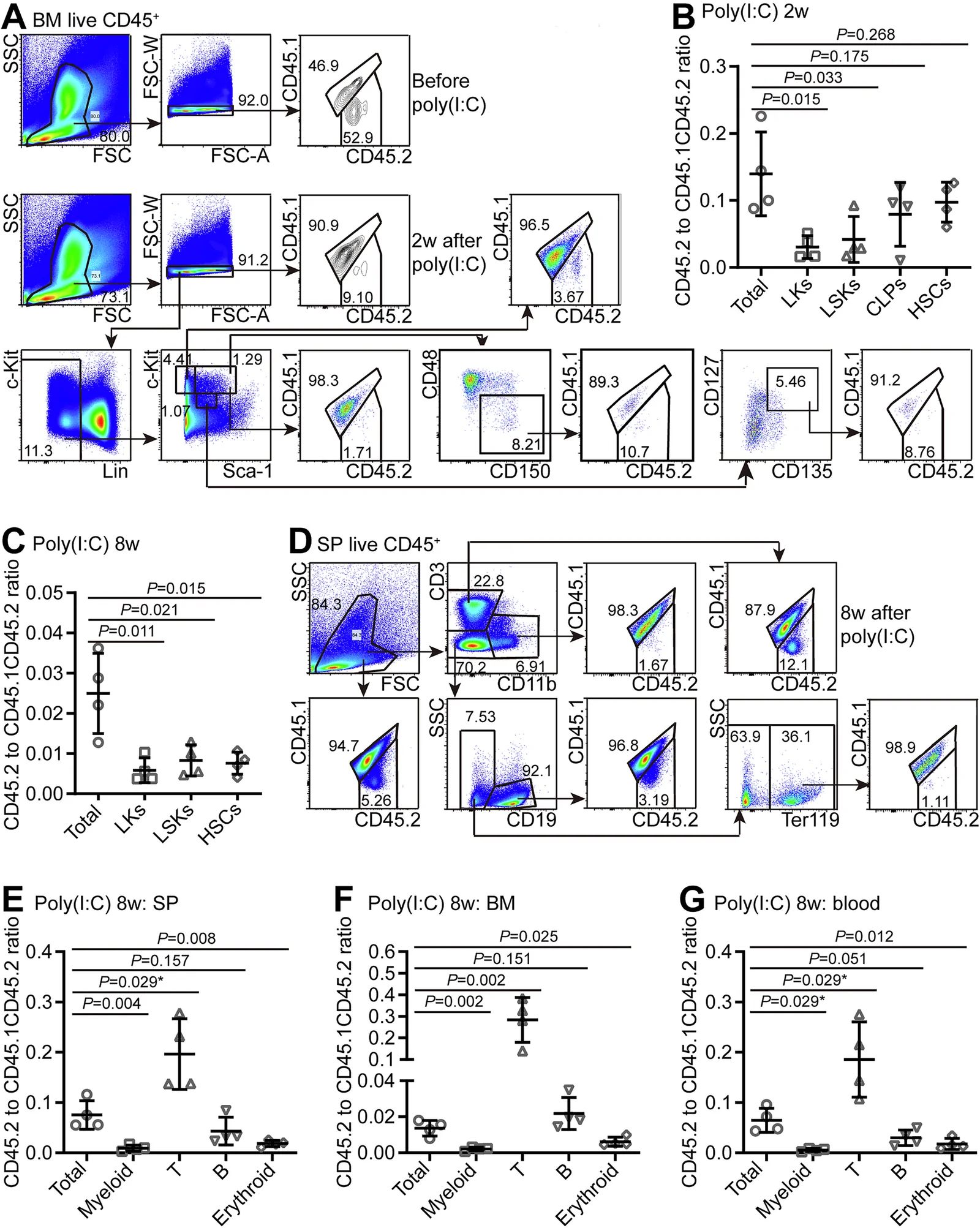
Rapid HSC exhaustion upon adulthood *Rack1* deletion results from cell-intrinsic defects. BM cells from CD45.1CD45.2 cWT and CD45.2 cKO mice were co-transferred into lethally irradiated CD45.1 WT recipients at the ratio of 1:1. After 8 weeks of BM reconstitution, chimeras (*n* = 4 mice/group) were subjected to the following assays. **A** Representative gating strategy for flow cytometry analysis of the chimerism of total leukocytes, LKs, LSKs, CLPs, and HSCs in the BM before and 2 weeks after poly(I:C) injection. CD45.2 to CD45.1CD45.2 ratios of total leukocytes, LKs, LSKs, CLPs, and HSCs in the BM 2 (**B**) and 8 (**C**) weeks after poly(I:C) injection. **D** Representative gating strategy for flow cytometry analysis of the chimerism of total leukocytes, myeloid cells, T cells, B cells, and erythroid cells in the spleen (SP) 8 weeks after poly(I:C) injection. CD45.2 to CD45.1CD45.2 ratios of total leukocytes, myeloid cells, T cells, B cells, and erythroid cells in the SP (**E**), BM (**F**), and blood (**G**) 8 weeks after poly(I:C) injection. Error bars show mean ± SD. *P* values are shown above with two-tailed unpaired Student’s *t* test or Wilcoxon rank-sum test (indicated with the symbol ‘*’). Data are representative of two independent experiments.

To further distinguish cell-intrinsic or -extrinsic mechanisms underlying the rapid HSC loss, we created mice with mixed BM through transferring BM cells from CD45.1CD45.2 cWT mice and CD45.2 cKO mice into lethally irradiated CD45.1 WT mice at the ratio of 1:1. After 8 weeks of BM reconstitution, about half BM leukocytes in the chimeras came from CD45.2 donor cells (Fig. 4A). These chimeric mice were then treated with a single injection of 10 mg/kg poly(I:C). As expected, CD45.2 donor cells were specifically lost as the CD45.2 to CD45.1CD45.2 ratio of total BM leukocytes decreased to around 0.15 2 weeks after poly(I:C) injection, much lower than a hypothetical ratio of 1 (Fig. 4A, B). In particular, the CD45.2 to CD45.1CD45.2 ratios of LKs and LSKs were even lower than that of total leukocytes in the BM, although those of CLPs and HSCs were largely comparable to that of total leukocytes (Fig. 4A, B). Eight weeks after poly(I:C) injection, the CD45.2 to CD45.1CD45.2 ratio of total BM leukocytes further dropped to around 0.02 (Fig. 4C). Notably, the CD45.2 to CD45.1CD45.2 ratios of LKs, LSKs, and HSCs were all lower than that of total leukocytes in the BM (Fig. 4C). We also checked the chimerism of different leukocyte lineages in the spleen, BM, and blood at this time point. Indeed, the CD45.2 to CD45.1CD45.2 ratios of total leukocytes across tissues were all very low 8 weeks after poly(I:C) injection (Fig. 4D−G). Thus, the rapid hematopoietic failure upon adulthood *Rack1* deletion results from cell-intrinsic defects. In particular, CD45.2 donor-derived myeloid and erythroid cells were most severely affected whereas CD45.2 donor-derived T cells gained a selective advantage across tissues (Fig. 4D−G). As for B cells, their CD45.2 to CD45.1CD45.2 ratio tended to be higher than that of total leukocytes in the BM but tended to be lower in the spleen and blood (Fig. 4D−G), echoing our previous finding that RACK1 deficiency blocks B-cell development at the pro-B-cell stage [34].

### Adulthood *Rack1* deletion affects the survival of HSCs

Next, we tried to explore the mechanism(s) underlying the progressive loss of HSCs after Cre induction. We evaluated the effect of *Rack1* deletion on the proliferation of HSCs with thymidine analog BrdU incorporation assays. A higher proportion of HSCs in cKO mice incorporated BrdU, as compared with cWT mice (Fig. S4A, B). Such a defect was not cell-intrinsic because augmented BrdU incorporation by RACK1-deficient HSCs was not observed in chimeric mice (Fig. S4C, D). We also analyzed whether enhanced apoptosis occurred upon *Rack1* deletion by performing Annexin V staining. As expected, more HSCs in cKO mice underwent apoptosis, as compared with cWT mice (Fig. S4E, F). Such a defect was cell autonomous because augmented apoptosis of RACK1-deficient HSCs was also observed in chimeric mice (Fig. S4G, H). Collectively, these data indicate that the progressive loss of HSCs upon *Rack1* deletion may be attributed, at least partially, to their defective survival.

### Adulthood *Rack1* deletion in IFN-I-responsive cells leads to aberrant lineage-geneset-scores of transcriptional HSCs and the emergence of stressed HSCs

**Fig. 5:**
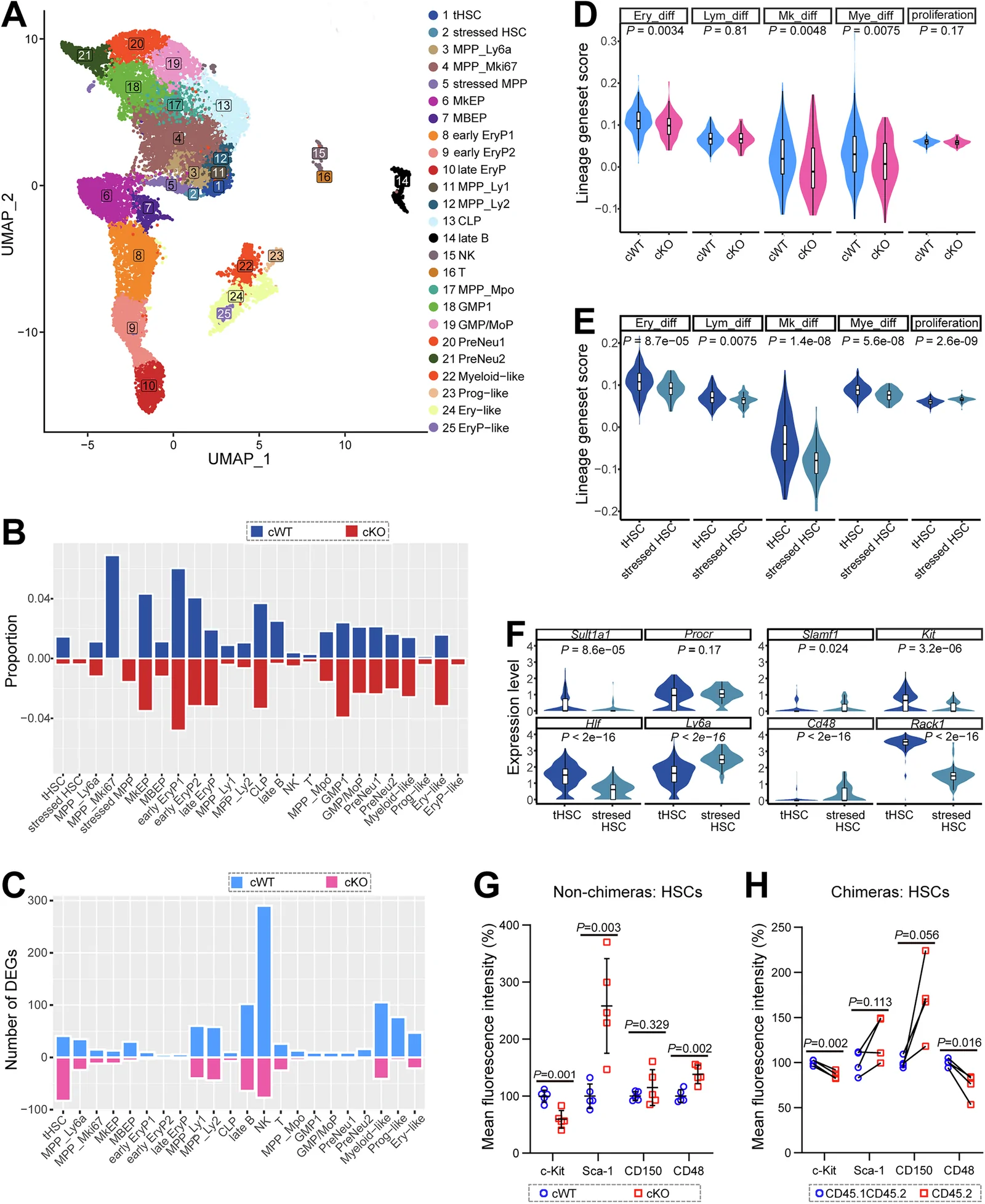
Adulthood *Rack1* deletion leads to aberrant transcriptional HSCs and the emergence of stressed HSCs. Twelve days after adult cWT and cKO mice were injected with poly(I:C), live Lin^−/low^c-Kit^+^ BM singlets were sorted and subjected to scRNA-seq. **A** Uniform manifold approximation and projection (UMAP) visualization of 17,216 qualified cells (cWT: 8,745, cKO: 8,471). Each dot represents an individual cell, and different colors mean different cell populations. **B** Proportion of each cluster as separated by different genotypes. **C** The number of differentially expressed genes (DEGs) between different genotypes in each cluster if either genotype contained ≥ 10 cells. **D** Geneset-scores of cWT and cKO tHSCs about erythroid differentiation (Ery_diff), lymphoid differentiation (Lym_diff), megakaryocyte differentiation (Mk_diff), myeloid differentiation (Mye_diff), and proliferation signature. **E** Geneset-scores of tHSCs and stressed HSCs. **F** Expression levels of key genes in tHSCs and stressed HSCs. Mean fluorescence intensity (MFI) of c-Kit, Sca-1, CD150, and CD48 on cWT and cKO HSCs described in Fig. 3A, D, E (**G**) and Fig. 4A, B (**H**). Error bars show mean ± SD. For MFI data, *P* values are shown above with two-tailed unpaired (**G**) or paired (**H**) Student’s *t* test. Data are representative of three (**G**) or two (**H**) independent experiments.

To further explore the underlying mechanism(s), BM mononuclear cells of cWT and cKO mice were subjected to MACS enrichment of c-Kit^+^ cells 12 days after poly(I:C) injection, followed by FACS sorting of live Lin^−/low^c-Kit^+^ singlets and scRNA-seq. The datasets from two libraries were pooled in downstream analysis and MNN was used to correct batch effects [46]. After quality control, 8,745 and 8,471 cells from cWT and cKO mice, respectively, remained for further analysis. Data dimension reduction, clustering, and annotation led to the identification of 25 cell clusters including 5 HSPC clusters (tHSC, stressed HSC, MPP_Ly6a, MPP_Mki67, and stressed MPP), 5 clusters of megakaryocyte-erythrocyte lineage (MkEP, MBEP, early EryP1, early EryP2, and late EryP), 6 lymphoid clusters (MPP_Ly1, MPP_Ly2, CLP, late B, NK, and T), 5 myeloid clusters (MPP_Mpo, GMP1, GMP/MoP, PreNeu1, and PreNeu2), and 4 clusters with both myeloid and erythroid characteristics (Myeloid-like, Prog-like, Ery-like, and EryP-like) (Figs. 5A and S5). Notably, the HSC cluster (referred as tHSC to distinguish from HSCs identified by flow cytometry) showed obvious enrichment of mouse HSC markers such as *Procr*, *Sult1a1*, *Ly6a* (Sca-1), *Hlf*, *Esam*, *Ifitm1*, and *Adgrl4* (Fig. S5). Meanwhile, stressed HSCs and stressed MPPs exhibited enrichment of *Gdf15* (Fig. S5), a marker of cellular stress [47], and thereby got the nomenclature. All clusters identified contained cells from cKO mice, although with varied proportions (Fig. 5B). Among HSPCs, MPP-Ly6a cells from different genotypes occupied a similar proportion. tHSCs and MPP_Mki67 cells from cKO mice occupied much lower proportions than their counterparts from cWT mice. By contrast, stressed HSCs and stressed MPPs came almost exclusively from cKO mice (Fig. 5B).

Differential expression analysis was employed to compare the transcriptomes between different genotypes in each cluster if either genotype contained ≥10 cells. Clusters in megakaryocyte-erythrocyte and myeloid lineages showed few DEGs (Fig. 5C). By contrast, clusters in the lymphoid lineage exhibited more DEGs except for CLP (Fig. 5C). tHSC, MPP-Ly6a, Myeloid-like, Prog-like, and Ery-like clusters also showed dozens of DEGs (Fig. 5C). In line with our aforementioned flow cytometry data, cKO tHSCs showed reduced erythroid, megakaryocyte, and myeloid putative differentiation potential but unaltered lymphoid putative differentiation potential, as compared with cWT tHSCs (Fig. 5D). However, cKO tHSCs showed the tendency of a lower proliferation signature than cWT tHSCs (Fig. 5D). In this scenario, we also analyzed the stressed HSC cluster, which is in close correlation with tHSC (Fig. S6A). Monocle 3 trajectory analysis and diffusion map trajectory analysis revealed that stressed HSCs were located downstream of tHSCs on the developmental trajectory (Fig. S6B). Notably, the stressed HSC cluster showed no connection with more mature subpopulations (Fig. S6B), suggesting that it may represent a specific cell state that has lost both the molecular signatures of HSCs and lineage differentiation capacity. Indeed, the stressed HSC cluster showed considerably reduced erythroid, megakaryocyte, and myeloid putative differentiation potential, in comparison to tHSC. Its lymphoid putative differentiation potential was also lower albeit to a less extent (Fig. 5E). Importantly, this cluster exhibited a higher proliferation signature than tHSC (Fig. 5E). Moreover, GO_BP enrichment revealed that this cluster showed molecular enrichment in apoptosis with terms such as “intrinsic apoptotic signaling pathway by p53 class mediator” and “intrinsic apoptotic signaling pathway in response to endoplasmic reticulum stress” (Fig. S7). Intriguingly, GO_BP enrichment also highlighted IFN-I signaling with terms such as “response to virus” and “interferon-beta production” (Fig. S7).

Then, we tried to further characterize stressed HSCs. In comparison to tHSCs, stressed HSCs showed reduced levels of *Sult1a1*, *Procr*, *Hlf*, and *Kit*, suggesting reduced pluripotency (Fig. 5F). Even though conditional deletion of exon 2 may not affect the mRNA level of *Rack1*, stressed HSCs contained a much lower level of *Rack1* than tHSCs (Fig. 5F). Meanwhile, stressed HSCs showed significantly higher levels of *Ly6a* and *CD48* and a slightly higher level of *Slamf1* (CD150) (Fig. 5F). In this scenario, we re-examined the flow cytometry data of HSCs. As expected, flow cytometry-identified HSCs in cKO mice showed significantly lower c-Kit mean fluorescence intensity (MFI), significantly higher Sca-1 MFI, comparable CD150 MFI, and slightly higher CD48 MFI 2 weeks after Cre induction, as compared with their counterparts in cWT mice (Fig. 5G). Thus, flow cytometry-identified HSCs in cKO mice 2 weeks after Cre induction should contain both tHSCs and stressed HSCs identified by scRNA-seq, mainly reflecting the characteristics of stressed HSCs about marker expression and proliferation. HSCs in WT→cKO recipients showed slightly lower MFI of c-Kit but comparable MFI of Sca-1, CD150, and CD48 2 weeks after Cre induction, as compared to their counterparts in WT→cWT recipients (Fig. S8A). On the other hand, HSCs in cKO→WT recipients showed significantly lower MFI of c-Kit, Sca-1, and CD150 but slightly higher MFI of CD48 2 weeks after Cre induction, as compared to their counterparts in cWT→WT recipients (Fig. S8B). In the chimeras, HSCs from cKO mice exhibited slightly lower c-Kit MFI, a tendency of higher Sca-1 and CD150 MFI, and slightly lower CD48 MFI 2 weeks after Cre induction, as compared to their counterparts from cWT mice (Fig. 5H). These data suggest that few or no stressed HSCs exist in WT→cKO recipients, cKO→WT recipients, and chimeric mice.

The downregulation of c-Kit and the upregulation of Sca-1 in HSCs from cKO mice diminished 6 weeks after poly(I:C) injection (Fig. S9A), suggesting the disappearance of the stressed HSC cluster. To test this notion, live Lin^−/low^c-Kit^+^ singlets isolated from cWT and cKO mice 6 weeks after poly(I:C) injection were also subjected to scRNA-seq. The datasets from two libraries were pooled, as addressed above. After quality control, 10,705 and 6525 cells from cWT and cKO mice, respectively, remained for further analysis. Data dimension reduction, clustering, and annotation were performed according to the conditions used for samples collected 12 days after poly(I:C) injection. These efforts resulted in the identification of 17 cell clusters, including 2 HSPC clusters (tHSC and MPP_Ly6a), 5 clusters of megakaryocyte-erythrocyte lineage (MkEP, MBEP, early EryP1, early EryP2, and late EryP), 5 lymphoid clusters (MPP_Ly, CLP, late B, NK, and T), and 5 myeloid clusters (MPP_Mpo, GMP1, GMP/MoP, PreNeu1, and PreNeu2) (Fig. S9B). Among HSPCs, tHSCs from different genotypes showed a similar proportion (Fig. S9C). In line with the findings that RACK1-deficient stromal cells tend to affect LSKs (Fig. S2C−E), MPP_Ly6a cells from cKO mice occupied a lower proportion than their counterparts from cWT mice (Fig. S9C). As compared to the data in Fig. 5A, the disappearance of MPP_Mki67 cells from both cWT and cKO mice 6 weeks after poly(I:C) injection (Fig. S9B) indicates that these cells are shortly expanded by poly(I:C) injection. This situation is also applicable to clusters with both myeloid and erythroid characteristics (Figs. 5A and S9B). Due to the disappearance of stressed HSCs and stressed MPPs from cKO mice 6 weeks after poly(I:C) injection (Fig. S9B), apparently residual tHSCs in cKO mice that escape Cre-loxp system-driven recombination should be the origin of the later hematopoietic recovery.

### Prenatal deletion of *Rack1* in hematopoietic cells results in reduced and defective HSCs in the fetal liver

**Fig. 6:**
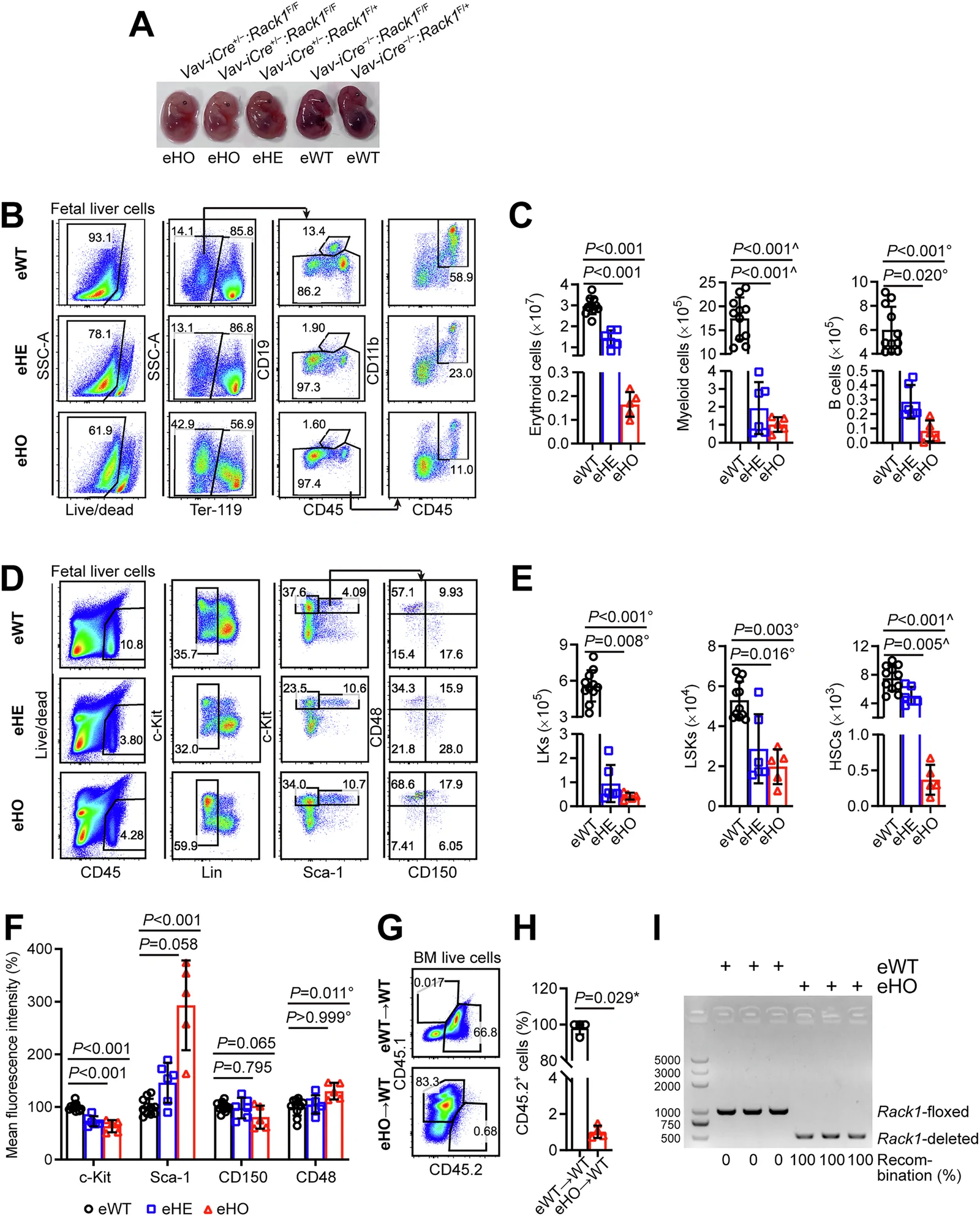
Prenatal deletion of *Rack1* in hematopoietic cells results in reduced HSCs in the fetal liver. **A–F** Embryonic day-14.5 *Vav-iCre*^+/−^*:Rack1*^F/F^ (eHO, *n* = 5), *Vav-iCre*^+/−^*:Rack1*^F/+^ (eHE, *n* = 6), *Vav-iCre*^−/−^*:Rack1*^F/F^ and *Vav-iCre*^−/−^*:Rack1*^F/+^ (eWT, *n* = 11) mice from three pregnant female mice were subjected to the following assays and their data were grouped together. Representative images of embryos (**A**). Representative gating strategy for flow cytometry analysis of erythroid, myeloid, and B cells in the fetal liver (**B**) and their corresponding numbers (**C**). Representative gating strategy for flow cytometry analysis of LKs, LSKs, and HSCs in the fetal liver (**D**) and their corresponding numbers (**E**). MFI of c-Kit, Sca-1, CD150, and CD48 on fetal liver HSCs (**F**). **G**, **H** Fetal liver cells from embryonic day-14.5 CD45.2 eWT mice (eWT→WT) and CD45.2 eHO mice (eHO→WT) were separately transferred into sublethally irradiated CD45.1 WT recipients. Eight weeks after transplantation, BM cells of recipients (*n* = 4 mice/group) were analyzed by flow cytometry. Representative gating strategy (**G**) and the frequency of CD45.2^+^ cells among total leukocytes (**H**) are shown. **I** Recombination efficiency of the conditional *Rack1* allele in live LSKs sorted from embryonic day-14.5 eWT and eHO mice (*n* = 3 mice/group). Error bars show mean ± SD. *P* values are shown above with one-way ANOVA, Welch ANOVA (indicated with the symbol ‘^’), Kruskal–Wallis test (indicated with the symbol ‘°’), or Wilcoxon rank-sum test (indicated with the symbol ‘*’). Data are representative of two independent experiments.

To further address the role of RACK1 in HSCs, we conditionally deleted *Rack1* from prenatal hematopoietic cells with mice expressing a transgene encoding an optimized variant of Cre recombinase under the control of mouse *Vav1* oncogene promoter [37]. *Vav-iCre*^+/−^*:Rack1*^F/F^ (referred to as embryonic homozygous, eHO) mice survived at expected Mendelian ratios to embryonic day-14.5 with the same morphology as control littermates (*Vav-iCre*^−/−^*:Rack1*^F/F^ or *Vav-iCre*^−/−^*:Rack1*^F/+^, referred to as embryonic wild type, eWT) and pale bodies (Fig. 6A). *Vav-iCre*^+/−^*:Rack1*^F/+^ (referred to as embryonic heterozygous, eHE) mice were also pale, although to a lesser extent (Fig. 6A). All eHO embryos died before birth although eHE mice can survive to adults. *Vav-iCre*-driven RACK1 deficiency in hematopoietic cells led to more death of fetal liver cells in a dose-dependent manner (Fig. 6B). Cells of different lineages were dramatically reduced in the liver of eHO embryos, as compared to control littermates (Fig. 6B, C). Notably, B cells were most severely affected (Fig. 6B, C), echoing the key role of RACK1 in B-cell development [34]. eHE mice showed partially reduced erythroid cells but similarly severe reduction of myeloid cells and B cells to eHO mice (Fig. 6B, C). At the stem/progenitor cell level, eHO mice exhibited dramatic reduction of LKs and HSCs and partial reduction of LSKs, as compared to control littermates (Fig. 6D, E). eHE mice showed partial reduction of HSCs but similar reduction of LKs and LSKs to eHO mice (Fig. 6D, E). About the feature of stressed HSCs, flow cytometry-identified HSCs in eHO mice showed slightly lower c-Kit MFI, significantly higher Sca-1 MFI, largely unchanged CD150 MFI, and slightly upregulated CD48 MFI (Fig. 6F). The changes in the expression of these markers were even weaker in HSCs from eHE mice (Fig. 6F). Thus, prenatal deletion of *Rack1* in hematopoietic cells results in reduced HSCs in the fetal liver, with partially diminished characteristics of stressed HSCs.

To functionally assess residual HSCs in eHO embryos and their recovery potential, fetal liver cells from CD45.2 eWT mice (eWT→WT) and CD45.2 eHO mice (eHO→WT) were separately transplanted into lethally irradiated CD45.1 WT recipients. All eHO→WT recipients died during the second post-radiation week if 9 Gy was employed, although all eWT→WT recipients survived under the same conditions. In this scenario, the irradiation dose was reduced to 8 Gy. All recipients survived at this dose of irradiation. After 8 weeks of BM reconstitution, all BM leukocytes in eWT→WT transplantation were CD45.2^+^ whereas almost all BM leukocytes in eHO→WT transplantation were CD45.1^+^ (Fig. 6G, H). Therefore, residual HSCs in eHO embryos lose their function and show no recovery potential. Accordingly, PCR analysis revealed 100% recombination efficiency of the *Rack1* allele in live LSKs sorted from eHO embryos (Fig. 6I). Apparently, eHO donor-derived HSCs and their downstream progenitors generate some blood cells, which help eHO→WT recipients survive until recipient-derived residual HSCs and their downstream progenitors expand and partially regenerate the hematopoietic system.

### RACK1 prevents HSC loss through maintaining the protein level of LDB1

**Fig. 7:**
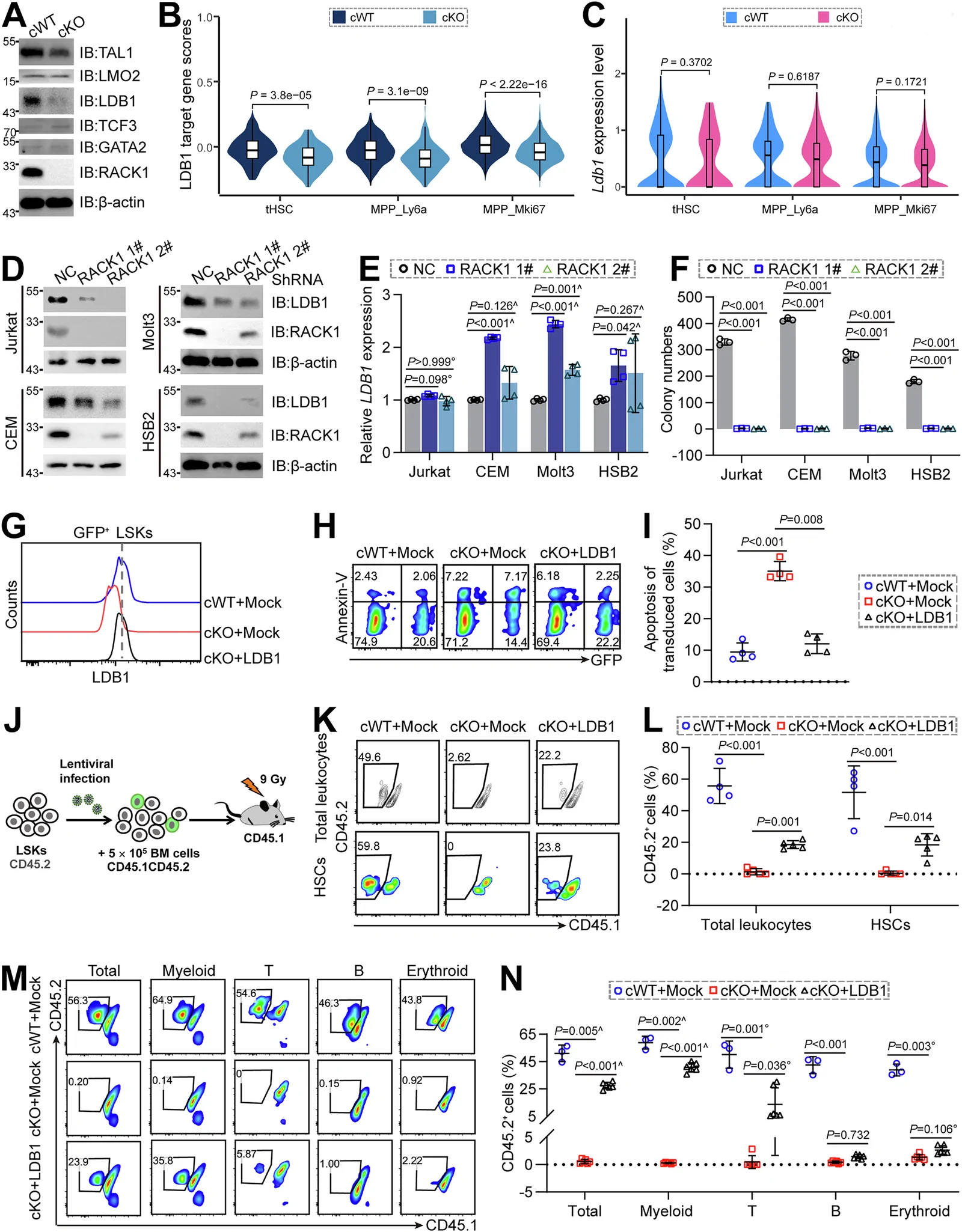
RACK1 prevents HSC loss through maintaining the protein level of LDB1. **A** Five days after adult cWT and cKO mice were injected with poly(I:C), live LSKs were sorted and subjected to IB with the indicated antibodies. LDB1 target gene scores (**B**) and mRNA levels (**C**) in cWT- and cKO-derived tHSC, MPP_Ly6a, and MPP_Mki67 cells identified in Fig. 5A−C. At 96 h after infection with a lentiviral vector carrying the indicated shRNA, live transduced (GFP^+^) T-ALL cells were sorted and subjected to IB (**D**), quantitative RT-PCR (**E**, *n* = 4/group), or soft-agar assays (**F**, *n* = 3/group). **G−L** Live LSKs were sorted from CD45.2 cWT and CD45.2 cKO mice 5 days after poly(I:C) injection and transduced with lentivirus expressing LDB1 or mock control lentivirus. The levels of LDB1 protein (**G**) and apoptosis (**H**, **I**, *n* = 4 mice/group) in transduced (GFP^+^) cells were examined by flow cytometry 96 h later. Live GFP^+^ cells were subsequently sorted, followed by transplantation into lethally irradiated CD45.1 recipients along with unfractionated BM support cells from CD45.1CD45.2 mice (**J**). Eight (**K**, **L** *n* = 4 ∼ 5 mice/group) or twenty (**M**, **N** *n* = 3 or 6 mice/group) weeks after transplantation, BM cells of recipients were analyzed by flow cytometry. Representative gating strategy (**K**, **M**) and the frequencies of CD45.2^+^ cells among total leukocytes and the indicated subpopulations (**L**, **N**) are shown. Error bars show mean ± SD. For statistical analysis other than scRNA-seq, *P* values are shown above with one-way ANOVA, Welch ANOVA (indicated with the symbol ‘^’), or Kruskal–Wallis test (indicated with the symbol ‘°’). Data are representative of two (**M**, **N**) or three (**A**, **D**−**F**, **G**) independent experiments or grouped from two independent experiments (**I**, **L**).

The above phenotypes suggest aberrant function of the core complex containing TFs TAL1/TCF3/GATA2 and adaptors LMO2/LDB1 in the absence of RACK1. Therefore, we set out to examine their protein levels in live LSKs sorted from cWT and cKO mice 5 days after poly(I:C) injection. IB analysis revealed a significant reduction of TAL1 and LDB1, a weak reduction of LMO2 and GATA2, but a slight increase of TCF3, in the absence of RACK1 (Fig. 7A). As *Ldb1* deletion results in more severe hematopoietic phenotypes than those observed with other factors in the core complex [22, 23], we decided to focus on LDB1. Indeed, gene set enrichment analysis indicated lower LDB1 target gene scores in tHSC, MPP_Ly6a, and MPP_Mki67 clusters from cKO mice than those from cWT mice 12 days after Cre induction (Fig. 7B). However, its mRNA level in these clusters was comparable between the two genotypes (Fig. 7C), suggesting that RACK1 maintains LDB1 expression at the post-transcription level.

To explore whether the RACK1-LDB1 axis is conserved in human HSCs, CD34^+^ human cord blood cells were employed and transduced with lentivirus carrying RACK1 shRNA or non-targeting control shRNA. IB confirmed the efficient knockdown of RACK1, which was associated with significant reduction of LDB1, TAL1, GATA2, and LMO2 but no change in TCF3 (Fig. S10A). In line with the notion that RACK1 maintains LDB1 expression at the posttranscription level, LDB1 mRNA was not reduced upon RACK1 knockdown (Fig. S10B). CFU assays indicated that RACK1-deficient CD34^+^ human cord blood cells lost the ability to form any type of myeloid colonies (Fig. S10C). LDB1 has been implicated in T-cell acute lymphoblastic leukemia (T-ALL) [48, 49]. In this scenario, human T-ALL cell lines Jurkat, CEM, Molt3, and HSB2 were also employed. As expected, lentivirus-mediated knockdown of RACK1 led to the reduction of LDB1 protein (Fig. 7D), but not its mRNA (Fig. 7E) in all four cell lines. Similar to the data obtained in CD34^+^ human cord blood cells (Fig. S10A), RACK1 deficiency also resulted in a significant reduction of TAL1, GATA2, and LMO2 in Molt3 and HSB2 cells (Fig. S10D). Accordingly, soft agar assays revealed that RACK1 knockdown hindered the colony-forming ability of all four T-ALL cell lines (Fig. 7F). As lentivirus-based Flag-LDB1 ectopic expression reversed the reduction of TAL1, GATA2, and LMO2 protein levels in Molt3 and HSB2 cells with RACK1 knockdown (Fig. S10D), LDB1 should be a key downstream factor of RACK1 in maintaining the expression of core complex components.

To address whether LDB1 is a major factor involved in the rapid HSC loss upon RACK1 deficiency, we performed another LDB1 complementation experiment. Live LSKs were purified from cWT and cKO mice 5 days after poly(I:C) injection and transduced with lentivirus expressing LDB1 or mock control lentivirus. Flow cytometry analysis demonstrated a lower LDB1 protein level (Fig. 7G) but a higher apoptosis level (Fig. 7H, I) in cKO LSKs than in cWT LSKs, which was fully reversed by lentivirus-based LDB1 expression. We further tried BM transplantation; lentivirus-transduced CD45.2 LSKs were injected into the tail vein of CD45.1 recipients alongside unfractionated BM support cells from CD45.1CD45.2 mice (Fig. 7J). After 8 weeks of BM reconstitution, flow cytometry analysis confirmed that LDB1 ectopic expression partially restored the hematopoietic reconstitution capacity of HSPCs with RACK1 deficiency (Fig. 7K, L). After 20 weeks of BM reconstitution, around 25% of total leukocytes in recipients injected with cKO LSKs expressing LDB1 were still CD45.2^+^ (Fig. 7M, N), indicating the restored function of long-term HSCs. About different leukocyte lineages, LDB1 ectopic expression potently rescued myeloid and T cells (Fig. 7M, N). Consistent with our previous findings that RACK1 is indispensable for B-cell development via stabilizing Pax5 [34], LDB1 complementation failed to rescue B cells (Fig. 7M, N). Unexpectedly, the rescue effects of LDB1 ectopic expression on erythroid cells were also marginal (Fig. 7M, N).

### RACK1 directly binds to LDB1 and prevents its ubiquitination

**Fig. 8:**
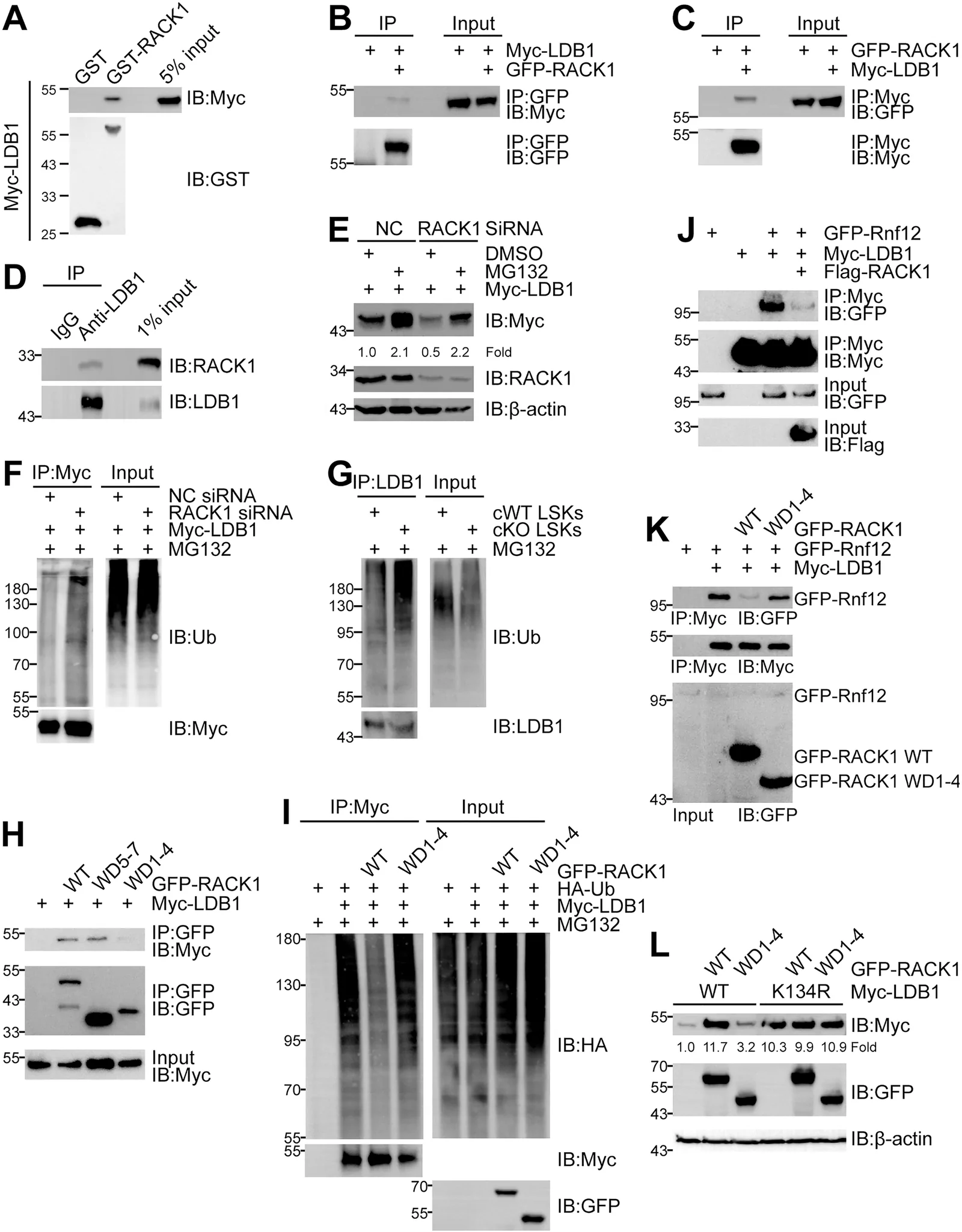
RACK1 directly binds to LDB1 and prevents its ubiquitination. **A** GST or GST-RACK1 bound to glutathione-Sepharose beads were incubated with lysates of HEK-293T cells expressing Myc-tagged core complex components. Precipitates were subjected to IB with the indicated antibodies. At 24 h after transfection with the indicated mammalian expression vectors, HEK-293T cells were subjected to immunoprecipitation (IP) with an antibody against GFP (**B**, **H**) or Myc-tag (**C**, **J**, **K**). Precipitates were then subjected to IB with these two antibodies. **D** MACS-enriched BM c-Kit^+^ cells from WT mice were subjected to IP with an anti-LDB1 antibody (control antibody: rabbit IgG). Precipitates were then subjected to IB with antibodies against RACK1 and LDB1. **E**, **F**, **I**, **L** At 42 h after transfection with mammalian expression vector(s) in the presence or absence of the indicated siRNA, HEK-293T cells were treated with or without 20 µM MG132 or DMSO of an equal volume for 6 h. The protein level of Myc-LDB1 was examined by IB (**E**, **L**). Densitometric readings are shown for Myc-LDB1 and normalized with β-actin. Ubiquitination of Myc-LDB1 was analyzed by IB after IP under partially denatured conditions with an anti-Myc antibody (**F**, **I**). Ub, ubiquitin. **G** Five days after adult cWT and cKO mice were injected with poly(I:C), MACS-enriched BM c-Kit^+^ cells were harvested and treated with 20 µM MG132 for 6 h. Ubiquitination of endogenous LDB1 was analyzed via IB after IP under partially denaturing conditions with an anti-LDB1 antibody. Data in this figure are representative of three (**A−I**) or two (**J−L**) individual experiments.

Because RACK1 can affect the stability of its binding proteins through the ubiquitin-proteasome system [31–34] and LDB1 undergoes ubiquitination under certain circumstances [26], we next examined whether RACK1 affects the protein level of LDB1 by directly binding to it. Indeed, in vitro GST pull-down assays revealed that substantial Myc-tagged LDB1 (Fig. 8A), but not TAL1, LMO2, TCF3, and GATA2 (Fig. S11A), in lysates of 293 T cells was precipitated specifically by GST-RACK1, but not by GST alone. Furthermore, Myc-LDB1 co-precipitated with co-expressed GFP-RACK1 (Fig. 8B) and GFP-RACK1 co-precipitated with co-expressed Myc-LDB1 (Fig. 8C) in HEK-293T cells. The possible physiological interaction of LDB1 with RACK1 was confirmed by immunoprecipitating endogenous LDB1 in MACS-enriched BM c-Kit^+^ cells from WT mice (Fig. 8D). In this scenario, HEK-293T cells were transfected with non-targeting control siRNA or siRNA against RACK1 and a mammalian expression vector encoding Myc-tagged TAL1, LMO2, LDB1, TCF3, or GATA2. IB revealed that RACK1 knockdown resulted in a reduced protein level of Myc-LDB1 (Fig. 8E), but not that of Myc-tagged TAL1, LMO2, TCF3, or GATA2 (Fig. S11B). The proteasome inhibitor MG132 reversed the reduction of Myc-LDB1 protein upon RACK1 deficiency (Fig. 8E). Accordingly, Myc-LDB1 ubiquitination in HEK-293T cells was augmented upon RACK1 knockdown (Fig. 8F). Notably, enhanced ubiquitination of endogenous LDB1 was observed in MACS-enriched BM c-Kit^+^ cells from cKO mice 5 days after poly(I:C) injection (Fig. 8G). RACK1 contains seven Trp‒Asp (WD) repeats [34]. While RACK1 truncated mutant containing WD repeats 5~7 (WD5-7) alone was sufficient to anchor LDB1, its truncated mutant containing WD repeats 1~4 (WD1-4) lost such ability (Fig. 8H). As GFP-RACK1 WD1-4 only marginally affected the ubiquitination of Myc-LDB1 under the conditions that GFP-RACK1 WT showed potent inhibition (Fig. 8I), RACK1 hinders LDB1 ubiquitination and thereby stabilizes it through direct interaction.

LDB1 ubiquitination occurred at lysine 134 [26], which is mediated by E3 ubiquitin ligase RING finger protein 12 (Rnf12) [50, 51]. It was demonstrated that single-strand DNA-binding protein 2 protects LDB1 from proteasomal degradation by directly binding to LDB1 and preventing its interaction with Rnf12 [51]. We speculate that RACK1 might hinder LDB1 ubiquitination via the same mechanism. Indeed, Flag-RACK1 abrogated the interaction between Myc-LDB1 and GFP-Rnf12 in HEK-293T cells (Fig. 8J). However, GFP-RACK1 WD1-4 failed to affect the recruitment of co-expressed E3 ligase by Myc-LDB1 under the same conditions that GFP-RACK1 WT showed potent effects (Fig. 8K). Accordingly, the protein level of Myc-LDB1 K134R was always higher than that of Myc-LDB1 WT when the same amount of plasmid was used (Fig. 8L). GFP-RACK1 WT significantly augmented the protein level of Myc-LDB1 WT but GFP-RACK1 WD1-4 showed diminished effects (Fig. 8L). Under the same conditions, neither GFP-RACK1 WT nor its truncated mutant defective of anchoring LDB1 affected the protein level of Myc-LDB1 K134R (Fig. 8L). These data suggest that RACK1 prevents the recruitment of Rnf12 through directly binding to LDB1 and thereby mediates site-specific stabilization.

## Discussion

Our study indicates that RACK1 is indispensable for both fetal and adult hematopoiesis. RACK1 is enriched in mouse hematopoietic progenitor cells. Adulthood *Rack1* deletion in IFN-I-responsive cells leads to rapid and profound hematopoietic failure (Fig. S12), which is associated with rapid and profound loss of HSCs and downstream progenitors. The progressive loss of HSCs in this model may be attributed, at least partially, to massive apoptosis. BM transplantation through various strategies indicates that the rapid loss of HSCs and their defective survival result from cell-intrinsic defects. Moreover, *Vav-iCre*-driven *Rack1* deletion in prenatal hematopoietic cells results in reduced and defective HSCs in the fetal liver (Fig. S12).

Although predominantly distributed in the cytoplasm of various cells, RACK1 can interact with nuclear proteins and function in the nucleus. For example, RACK1 binds to Smad3 and hinders Smad3 DNA-binding [29, 30]. In this study, we show that RACK1 directly binds to LDB1. RACK1 maintains the protein level of LDB1 in both mouse and human HSPCs, which can not be explained by its mRNA level. Indeed, we have found that enhanced ubiquitination of LDB1 upon RACK1 deficiency. Moreover, the reduction of LDB1 protein upon RACK1 knockdown can be reversed by the proteasome inhibitor MG132. With RACK1 truncated mutants, we have further shown that RACK1 prevents LDB1 ubiquitination through direct anchoring and thereby hinders the recruitment of E3 ubiquitin ligase Rnf12 (Fig. S12). Importantly, LDB1 ectopic expression partially restores the function of RACK1-deficient HSPCs. Even though it was reported that LDB1 does not affect the apoptosis of fetal liver HSCs [22], in our hands, it fully reverses the augmented apoptosis of RACK1-deficient HSPCs in vitro. Intriguingly, even though RACK1 maintains the expression of TAL1, GATA2, and LMO2 in both mouse and human HSPCs, its effects on GATA2 and LMO2 are more profound in CD34^+^ human cord blood cells and human T-ALL cell lines than in mouse LSKs. Through analyzing how RACK1 knockdown in T-ALL cells might affect LDB1 expression and the colony-forming ability, we have also provided clinical implications of RACK1 disturbances in human hematopoiesis. Importantly, enforced LDB1 expression in the RACK1-deficient setting (using T-ALL cell lines) effectively restores the protein levels of these core complex components. These findings close the mechanistic loop.

Despite that, it should be pointed out that LDB1 complementation can’t fully restore the function of RACK1-deficient HSCs. Indeed, RACK1 is a multifaceted adaptor. By anchoring the 40S small ribosome subunit, RACK1 has been suggested to control selective mRNA translation [52]. Recently, ribosome assembly and protein synthesis have been revealed to be essential for HSC regeneration [53]. Meanwhile, RACK1 promotes the assembly of the autophagy initiation complex [35]. HSCs and various downstream progenitors are reduced in the absence of the key autophagy protein Atg7, although the number of LSKs is elevated [54]. Besides, RACK1 limits IFN-I production [55], which prevents IFN-I-driven HSC proliferation and apoptosis [56, 57]. Thus, multiple other signaling pathways regulated by RACK1 might also contribute to the hematopoietic aberrancies observed.

On the other hand, enforced LDB1 expression in RACK1-deficient HSPCs can’t fully restore impaired lineage differentiation either. LDB1 specifically rescues the myeloid/T-cell arms while leaving the B-cell/erythroid defects largely intact. The failure of LDB1 to rescue B-cell deficiency echoes our previous finding that RACK1 promotes B-cell development via stabilizing Pax5 [34]. Even though LDB1 is required for erythroid differentiation, its enforced expression blocks this process [58, 59]. The lentivirus vector we used to express LDB1 employs the powerful Ubc promoter, which might result in LDB1 overexpression in erythroid progenitors and thereby abrogate erythroid differentiation. On the other hand, there might be yet-to-be identified RACK1-binding partner(s) that are essential for erythroid differentiation.

Unexpectedly, scRNA-seq identified stressed HSCs and stressed MPPs, which come almost exclusively from cKO mice 12 days after Cre induction. Stressed HSCs are located downstream of tHSCs on the developmental trajectory. Due to the low *Rack1* level, these cells should come from HSCs with the recombination of both *Rack1* alleles. The features of stressed HSCs — particularly the Sca-1 upregulation, IFN-response gene enrichment, and a high apoptotic and proliferative signature — bear a striking resemblance to the well-characterized “IFN-activated” HSC state described by several groups [56, 57]. Accordingly, this cluster appears to deviate from normal hematopoietic development and disappears 6 weeks after poly(I:C) injection. Thus, the stressed HSC cluster represents a transient transcriptomic snapshot of cells in the process of apoptosis instead of a stable stress-induced state. This notion may also be applicable to stressed MPPs.

Stem cells exist in and depend on specific microenvironments [60]. The absence of RACK1 in hematopoietic cells leads to augmented IFN-I production, which should contribute to the enrichment of IFN-I signaling in stressed HSCs. Despite that, the absence of stressed HSCs in cWT/cKO chimeric mice and cKO→WT recipients indicates that RACK1 deficiency in hematopoietic cells is not enough for the generation of stressed HSCs. The deficiency of RACK1 in non-hematopoietic IFN-I-responsive cells, especially BM stromal cells, of cKO mice should also contribute to elevated IFN-I production. Among stromal cells, *Vav-iCre* only targets vascular endothelial cells and no poly(I:C) is required for Cre expression [37]; there should be lower levels of IFN-Is in the hematopoietic microenvironments of eHO mice than cKO mice. Accordingly, flow cytometry-identified HSCs in the fetal liver of eHO mice show partially diminished characteristics of stressed HSCs.

Flow cytometry-identified HSCs in cKO mice 2 weeks after Cre induction show high BrdU incorporation, mainly reflecting the high proliferative signature of stressed HSCs. However, this does not mean that the stressed HSC cluster is solely responsible for the bulk BrdU signal. It is known that flow cytometry-identified HSCs show transcriptional heterogeneity and contain a small portion functionally more associated with MPPs [45]. This portion might become enlarged upon RACK1 deficiency. Nevertheless, the enhanced proliferation in flow cytometry-identified HSCs should contribute to the later hematopoietic recovery.

This study has limitations. As stated above, *Mx1-Cre* and *Vav-iCre* target all hematopoietic cells and some stromal cells [36, 37]. HSC-specific models like *Fgd5-CreERT2* might provide strengthened clarification. Meanwhile, lineage-tracing of hematopoietic cells and stromal cells can confirm the origin and outcome of stressed HSCs and stressed MPPs. The notion that IFN-I signaling drives the emergence of stressed HSCs can be tested with neutralization antibodies to the receptor. These issues await future efforts. In addition, the role of RACK1 in erythroid differentiation remains to be addressed.

## Supplementary information

Supplementaldata

uncropped IB blots

## Data Availability

All data are available in the main text or in the supplementary information. All reagents reported in the paper may be available to the scientific community. All scRNA-seq data have been deposited in Gene Expression Omnibus. For samples collected 12 days after poly(I:C) injection, the accession number is GSE315108 with reviewer token ezojmuqetvolnan. For samples collected 6 weeks after poly(I:C) injection, the accession number is GSE315109 with reviewer token clmfwykmjhcvvud.
