# Supplementary material for: RACK1 maintains mouse hematopoietic stem cells by directly binding to and stabilizing LDB1
Source: Cell Death Dis. 2026 Aug 9;17(1):943. doi: 10.1038/s41419-026-09177-z (PMC13737179; doi:10.1038/s41419-026-09177-z)
Supplement: Supplementary file 1 [file 41419_2026_9177_MOESM1_ESM.pdf]

Supplementary Data for:

RACK1 maintains mouse hematopoietic stem cells by directly binding to and stabilizing LDB1

Authors: Lijiao Deng[1,2#], Junjie Du[3,4,#], Zhe Xu[1,5,#], Lu Guo[1], Qingyang Wang[1], Anbiao Wu[1], Guang Yang[1], Yu-chen Lu[1], Tingting Guo[1], Chunxiao Niu[1], Junxia Cao[1], Yu Lan[6,*], Bingshui Xiu[1,5,*], Jiannan Feng[2,*], Jiyan Zhang[1,5,7,*]

Supplementary Figures S1-S12 and Figure Legends

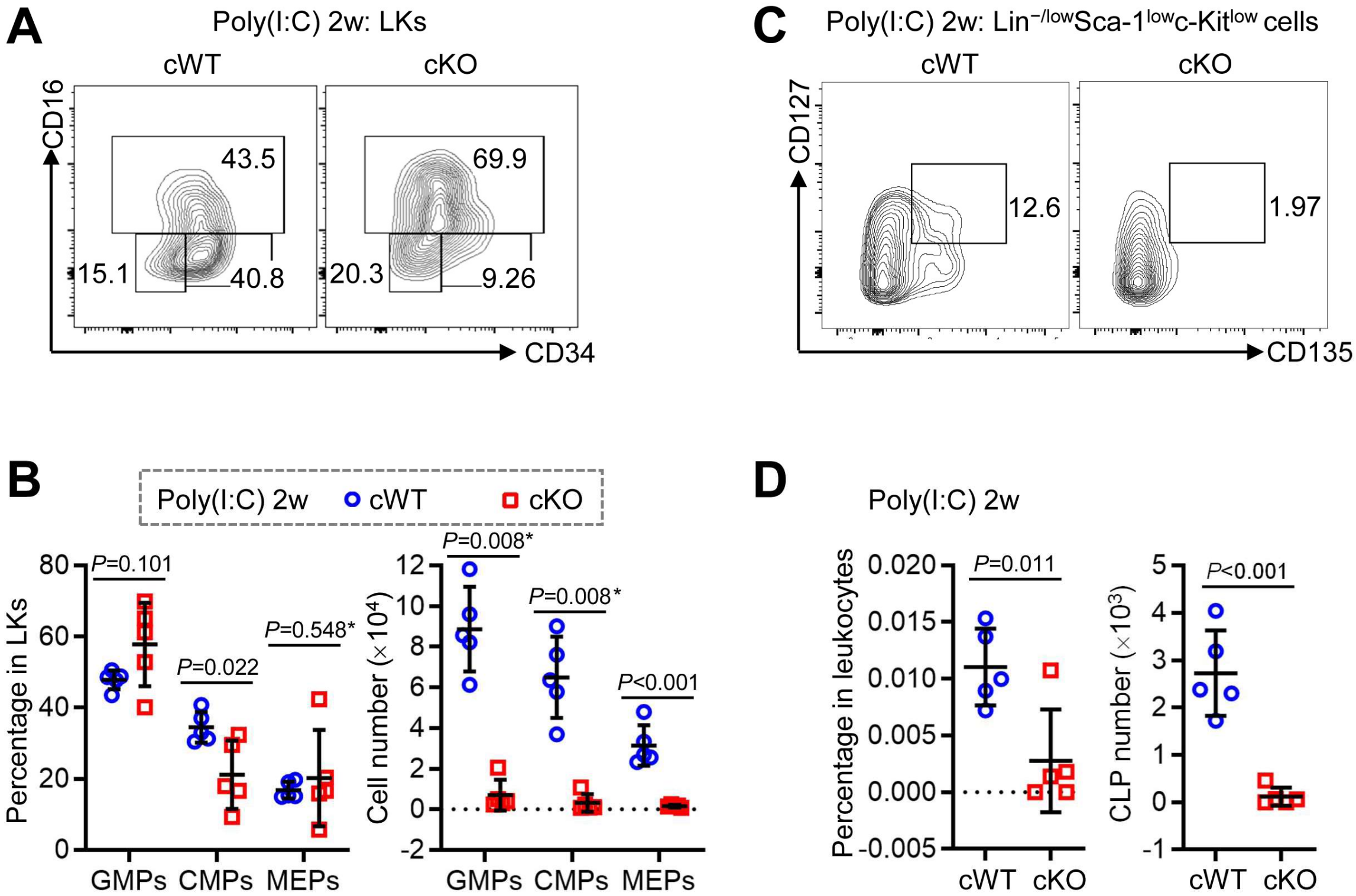

Fig. S1 Adulthood *Rack1* deletion in IFN-I-responsive cells leads to rapid loss of hematopoietic progenitors. Two weeks after poly(I:C) injection, adult *Rack1*$^{\mathrm{F/F;\ Mx1\text{-}Cre}}$ (cKO) mice and their littermates (cWT) (n = 5 mice/group) were subjected to the following assays. (**A**) Representative gating strategy for flow cytometry analysis of GMPs, CMPs, and MEPs among LKs. (**B**) The percentages of GMPs, CMPs, and MEPs among LKs (*left*) and their numbers in the BM (*right*). (**C**) Representative gating strategy for flow cytometry analysis of CLPs in Lin$^{-/\mathrm{low}}$Sca-1^{low}c-Kit^{low} compartment as indicated in Fig. 3A. (**D**) The frequency (*left*) and number (*right*) of CLPs in the BM. Error bars show mean ± SD. *P* values are shown above with two-tailed unpaired Student's *t* test or Wilcoxon rank-sum test (indicated with the symbol '*'). Data are representative of two independent experiments.

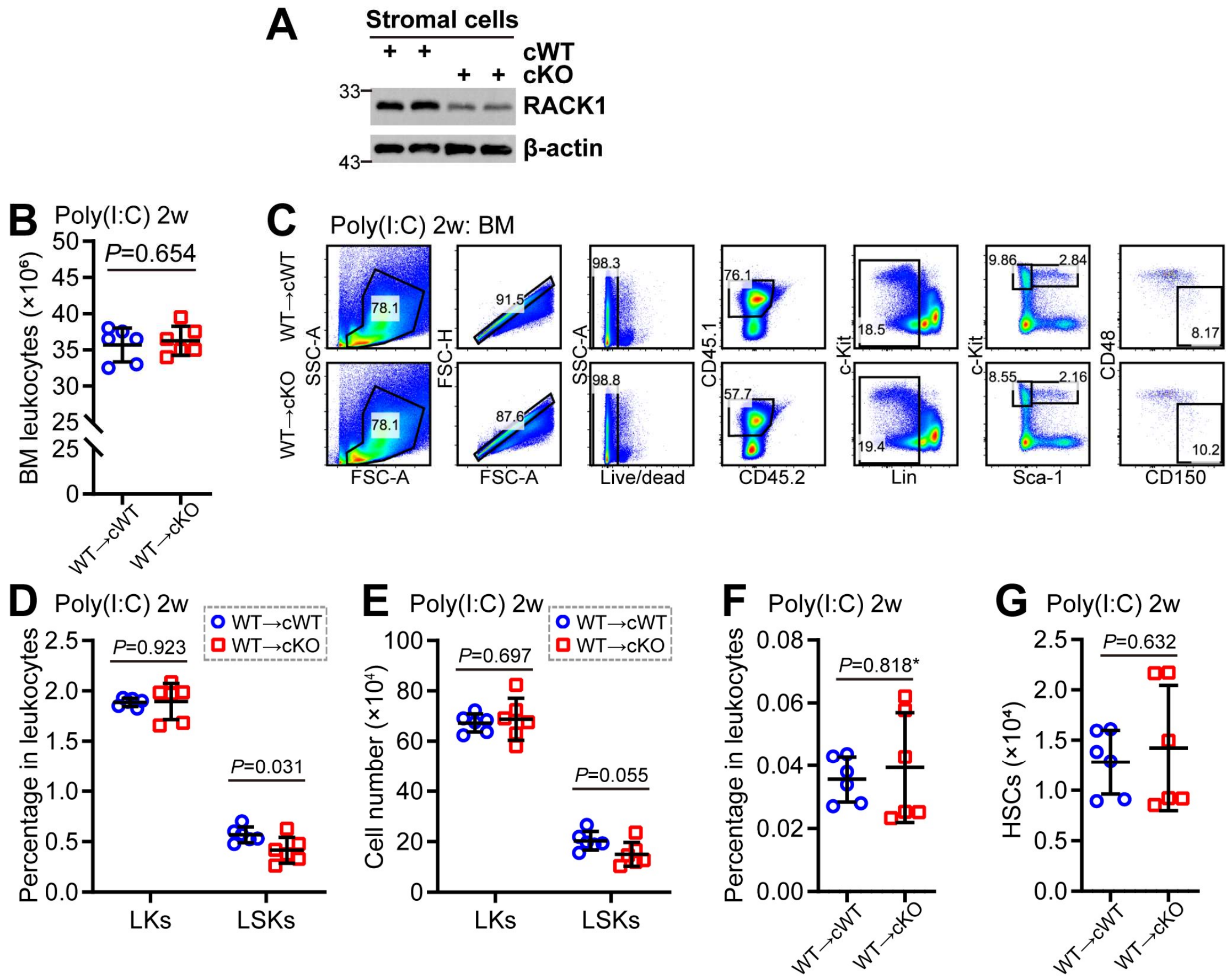

Fig. S2 The niche-specific effects are not a major contributor to the rapid hematopoietic failure upon adulthood *Rack1* deletion. (**A**) IB analysis of RACK1 expression in live $CD45^{-}Ter119^{-}$ BM stromal cells sorted from cWT and cKO mice (n = 2 mice/group) 2 weeks after Cre induction. (**B–G**) BM cells from CD45.1 WT mice were transplanted into lethally irradiated CD45.2 cWT mice (WT→cWT) and CD45.2 cKO mice (WT→cKO). After 8 weeks of BM reconstitution, these recipients were treated with a single injection of 10 mg/kg poly(I:C). Two weeks later, recipient mice (n = 6 mice/group) were subjected to the following assays. (**B**) The number of total BM cells in the femurs. (**C**) Representative gating strategy for flow cytometry analysis of LKs, LSKs, and HSCs in the BM. (**D**) The frequencies of LKs and LSKs. (**E**) The numbers of LKs and LSKs. (**F**) The frequency of HSCs. (**G**) The number of HSCs. Error bars show mean ± SD. P values are shown above with two-tailed unpaired Student's t test or Wilcoxon rank-sum test (indicated with the symbol '*').

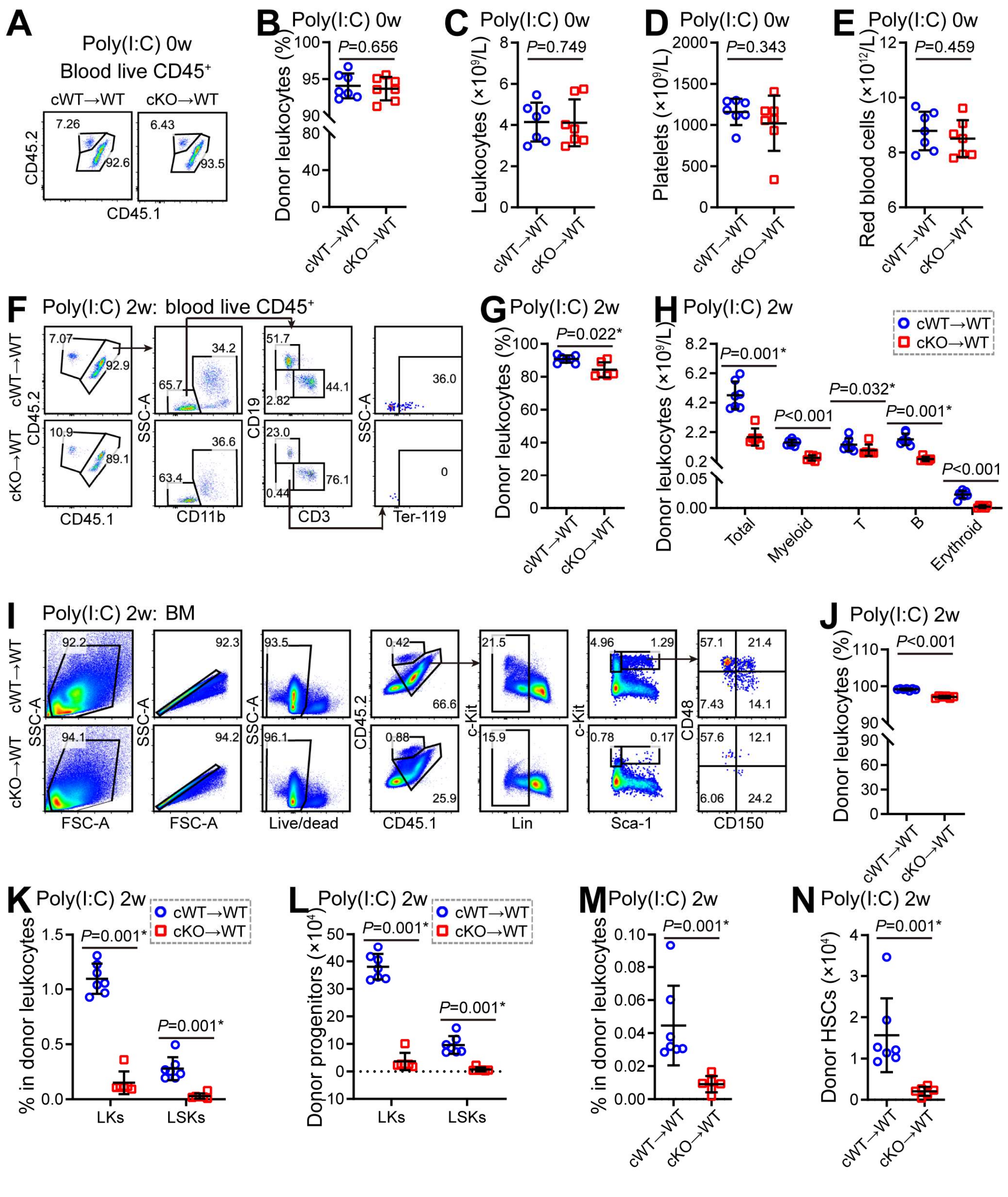

Fig. S3 Adulthood *Rack1* deletion in hematopoietic cells results in rapid hematopoietic failure. CD45.1CD45.2 cWT (cWT→WT, *n* = 7 mice) and CD45.1CD45.2 cKO (cKO→WT, *n* = 7 mice) BM cells were transplanted into lethally irradiated CD45.2 WT recipients. After 8 weeks of BM reconstitution, these recipients were treated with a single injection of 10 mg/kg poly(I:C). One cKO→WT recipient died 10 days after Cre induction. (**A**, **F**, **I**) Representative gating strategy for flow cytometry analysis of the chimerism and

donor-derived subsets. (**B**, **G**, **J**) The frequency of donor-derived leukocytes. (**C**–**E**) The numbers of blood total leukocytes (**C**), platelets (**D**), and red blood cells (**E**). (**H**) The numbers of donor-derived total leukocytes, myeloid cells, T cells, B cells, and $CD45^+$ erythroid cells in the blood. (**K**, **L**) The frequencies (**K**) and numbers (**L**) of donor-derived LKs and LSKs. (**M**, **N**) The frequency (**M**) and number (**N**) of donor-derived HSCs. Error bars show mean ± SD. *P* values are shown above with two-tailed unpaired Student's *t* test or Wilcoxon rank-sum test (indicated with the symbol '*').

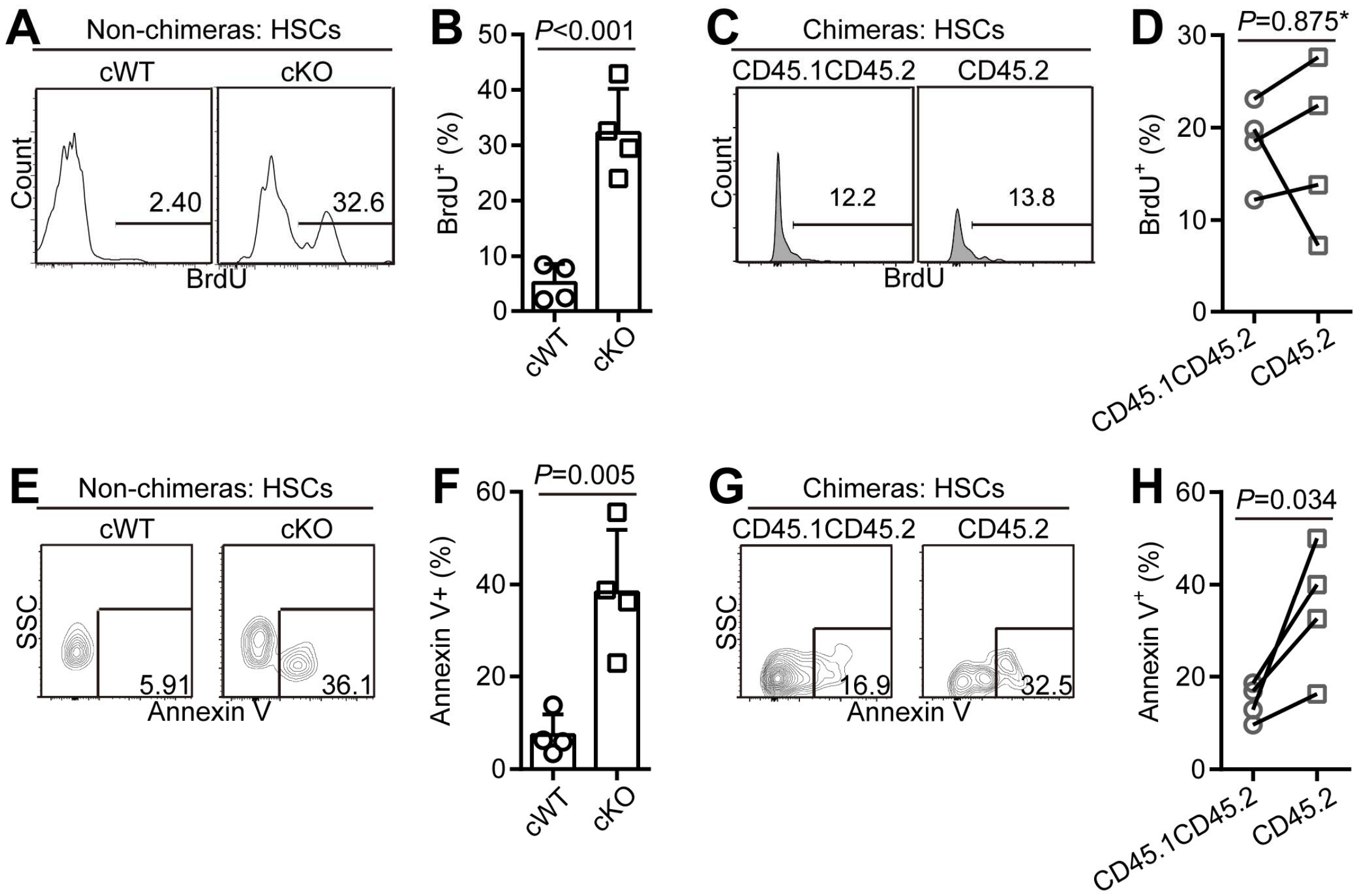

Fig. S4 Adulthood *Rack1* deletion affects the survival of HSCs. HSCs in adult cWT and cKO mice or the chimeras described in Fig. 4A, B (n = 4 mice/group) were analyzed by flow cytometry 2 weeks after poly(I:C) treatment. (**A**, **C**) Representative gating strategy for flow cytometry analysis of BrdU incorporation by HSCs 24 h after intraperitoneal injection of 1 mg thymidine analog BrdU in 0.1 mL PBS. (**B**, **D**) The frequency of $BrdU^+$ HSCs. (**E**, **G**) Representative gating strategy for flow cytometry analysis of Annexin V^+ HSCs. (**F**, **H**) The frequency of Annexin V^+ HSCs. Please note that in the chimeras, CD45.1CD45.2 donor-derived and CD45.2 donor-derived HSCs were separately analyzed. Error bars show mean ± SD. P values are shown above with unpaired (**B**, **F**) or paired (**H**) two-tailed Student's t test or Wilcoxon rank-sum test (**D**, indicated with the symbol '*').

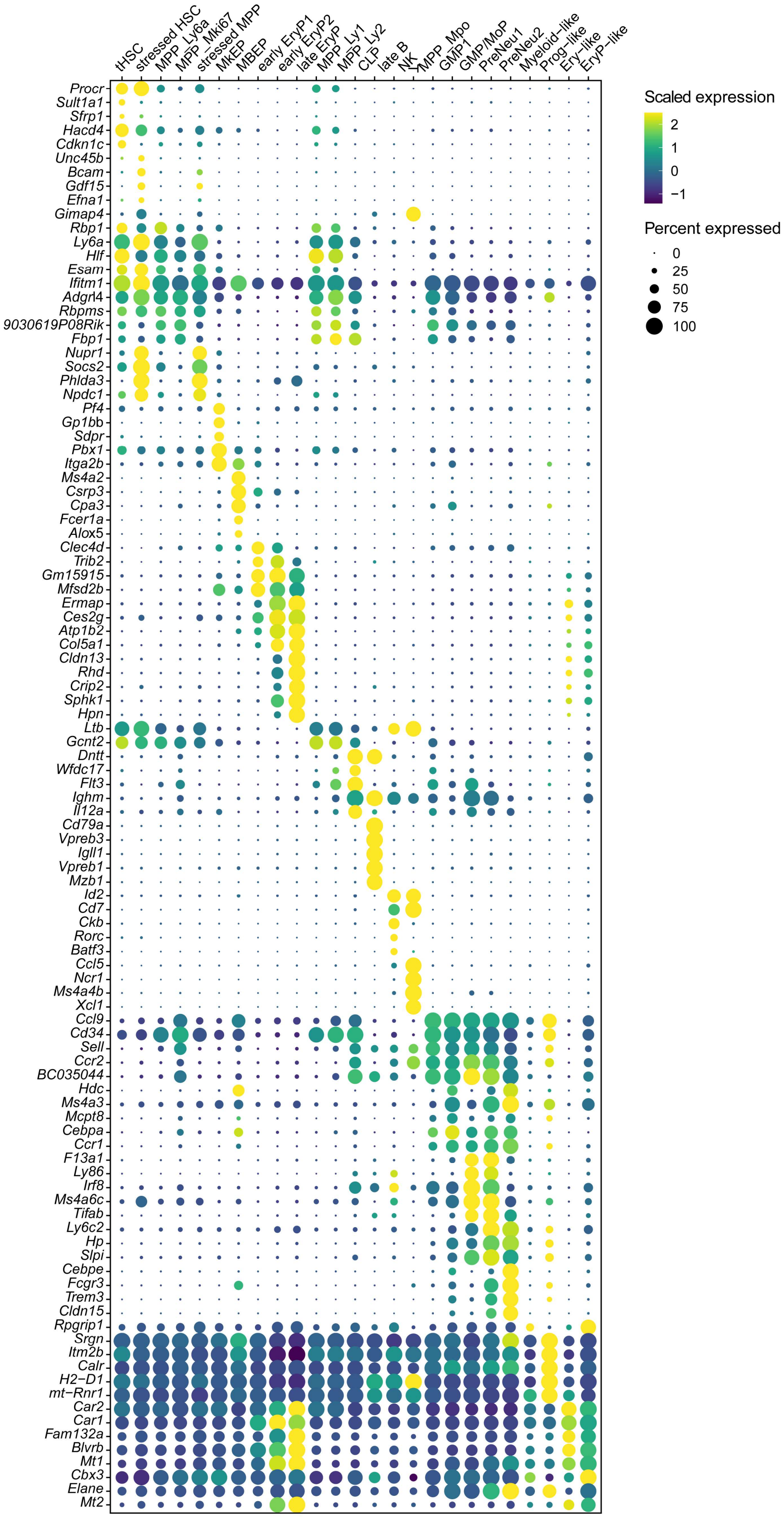

Fig. S5 Dot plots showing percentages of cells expressing marker genes and scaled gene expression in each cluster identified in Fig. 5A.

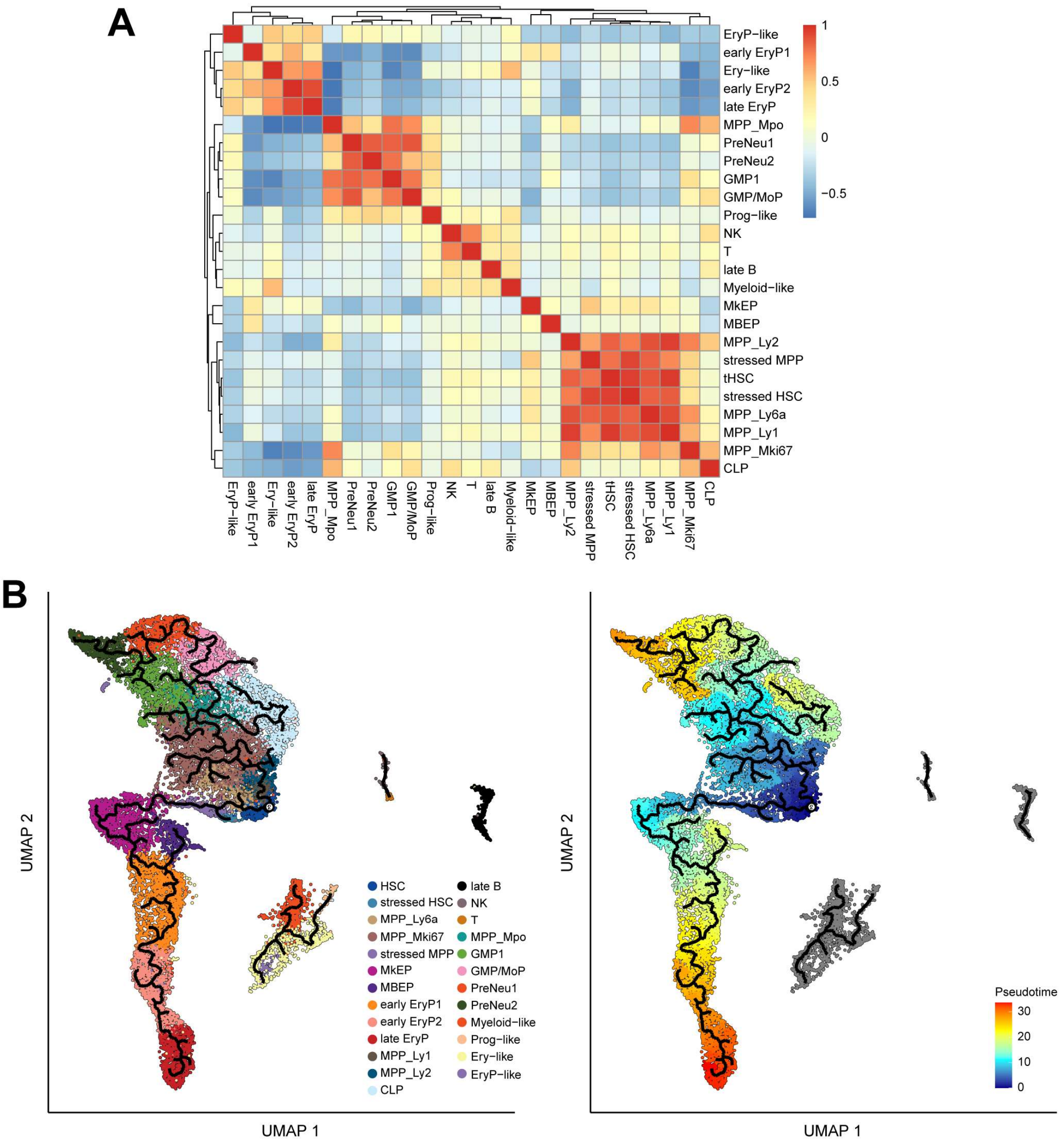

Fig. S6 Close correlation of the stressed HSC cluster with the tHSC cluster**.** (**A**) Pearson correlation coefficients between each pair of clusters identified in Fig. 5A. (**B**) Monocle 3 trajectory analysis of all clusters identified in Fig. 5A (*left*) and diffusion map trajectory analysis in the destiny package (*right*).

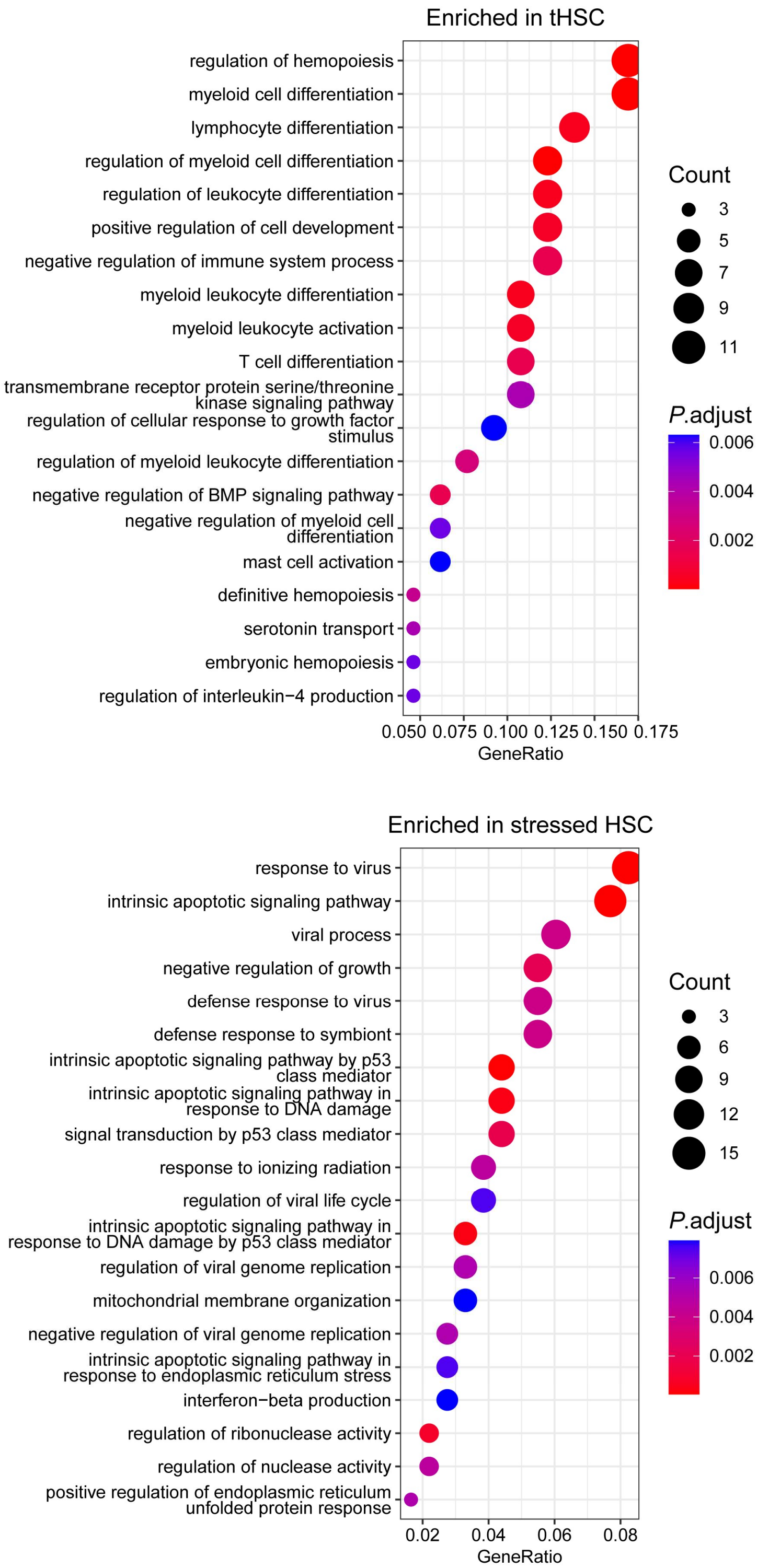

Fig. S7 Gene ontology biological process terms enriched in the tHSC cluster (*top*) and the stressed HSC cluster (*bottom*), respectively**.**

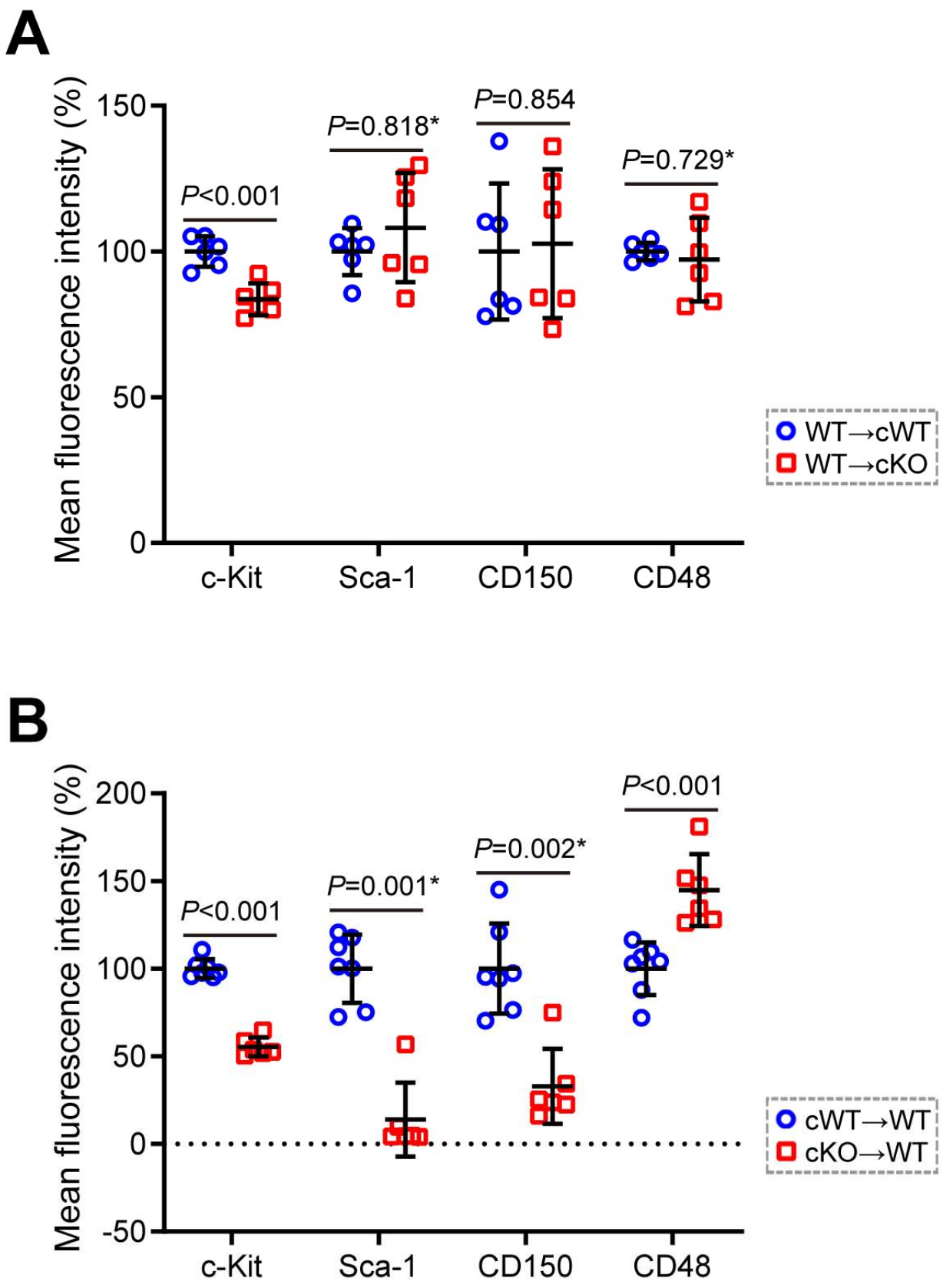

Fig. S8 RACK1 deficiency in stromal cells and RACK1 deficiency in hematopoietic cells plus severe HSC stress are not sufficient conditions for the generation of stressed HSCs**.** Mean fluorescence intensity (MFI) of c-Kit, Sca-1, CD150, and CD48 on HSCs described in Fig. S2C, F, G (**A**) and Fig. S3I, M, N (**B**). Error bars show mean ± SD. *P* values are shown above with two-tailed unpaired Student's *t* test or Wilcoxon rank-sum test (indicated with the symbol '*').

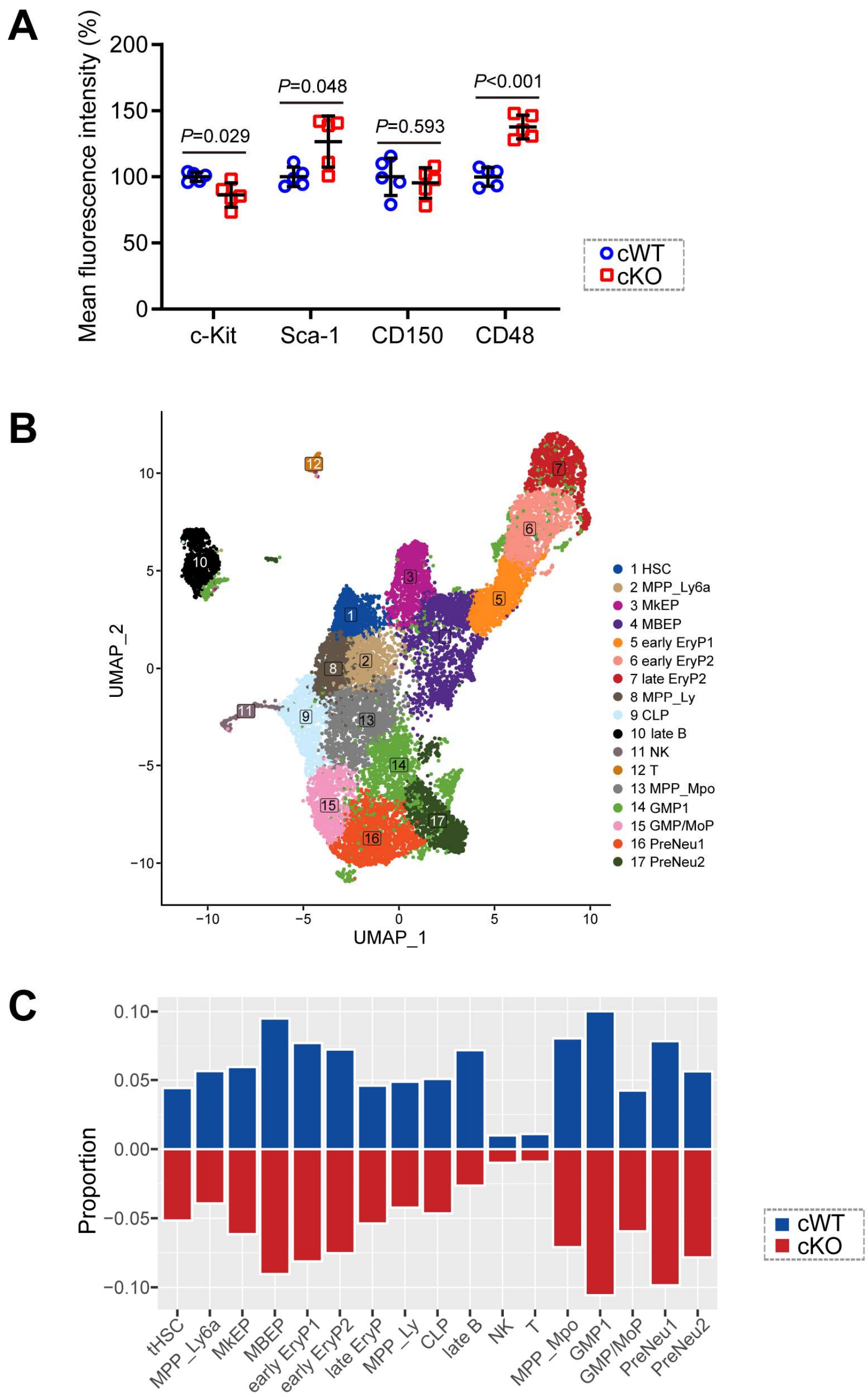

Fig. S9 Stressed HSCs in cKO mice diminish 6 weeks after poly(I:C) injection. (**A**) MFI of c-Kit, Sca-1, CD150, and CD48 on HSCs. Error bars show mean ± SD. *P* values are shown above with two-tailed unpaired Student's *t* test. (**B**) After scRNA-seq, UMAP visualization of 17,230 qualified live Lin$^{-/low}$c-Kit^{+} singlets (cWT: 10,705; cKO: 6,525). Each dot represents an individual cell, and different colors mean different cell populations. (**C**) Proportion of each cluster as separated by different genotypes.

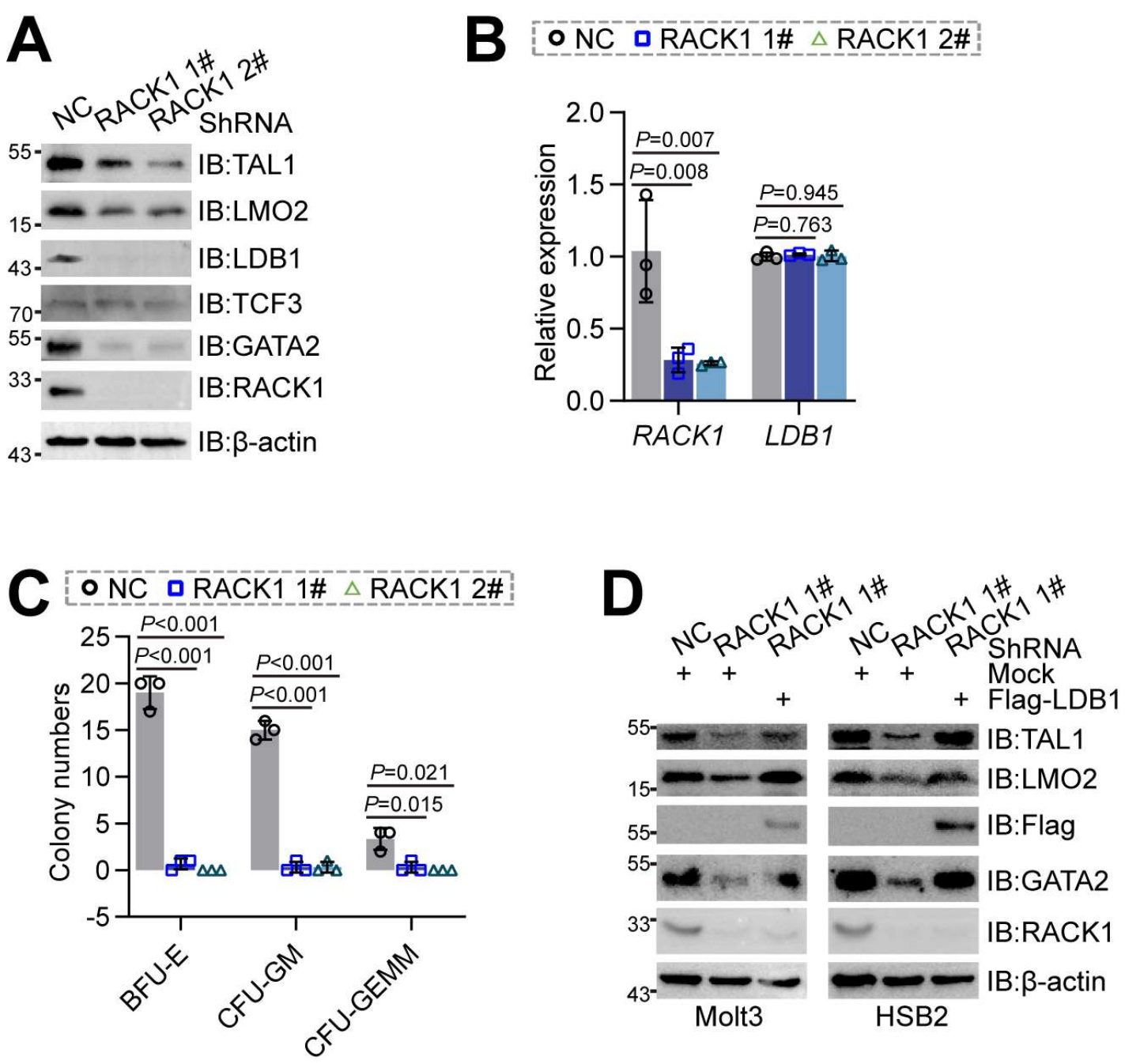

Fig. S10 The RACK1-LDB1 axis is conserved in human CD34$^+$ cord blood cells and T-ALL cells. (**A–C**) At 96 h after infection with a lentiviral vector carrying the indicated shRNA, live transduced (GFP$^+$) CD34$^+$ human cord blood cells were sorted and subjected to IB (**A**), quantitative RT-PCR (**B**, n = 3/group), or CFU assays (**C**, n = 3/group). BFU-E, burst-forming unit-erythrocytes; CFU-GM, CFU-granulocytes/macrophages; CFU-GEMM, CFU-granulocyte/erythrocyte/macrophage/megakaryocyte. (**D**) At 96 h after infection with a lentiviral vector carrying the indicated shRNA and lentivirus expressing Flag-LDB1 or mock control lentivirus, live transduced (GFP$^+$) T-ALL cells were sorted and subjected to IB. Error bars show mean ± SD. P values are shown above with one-way ANOVA. All data in this figure are representative of two independent experiments.

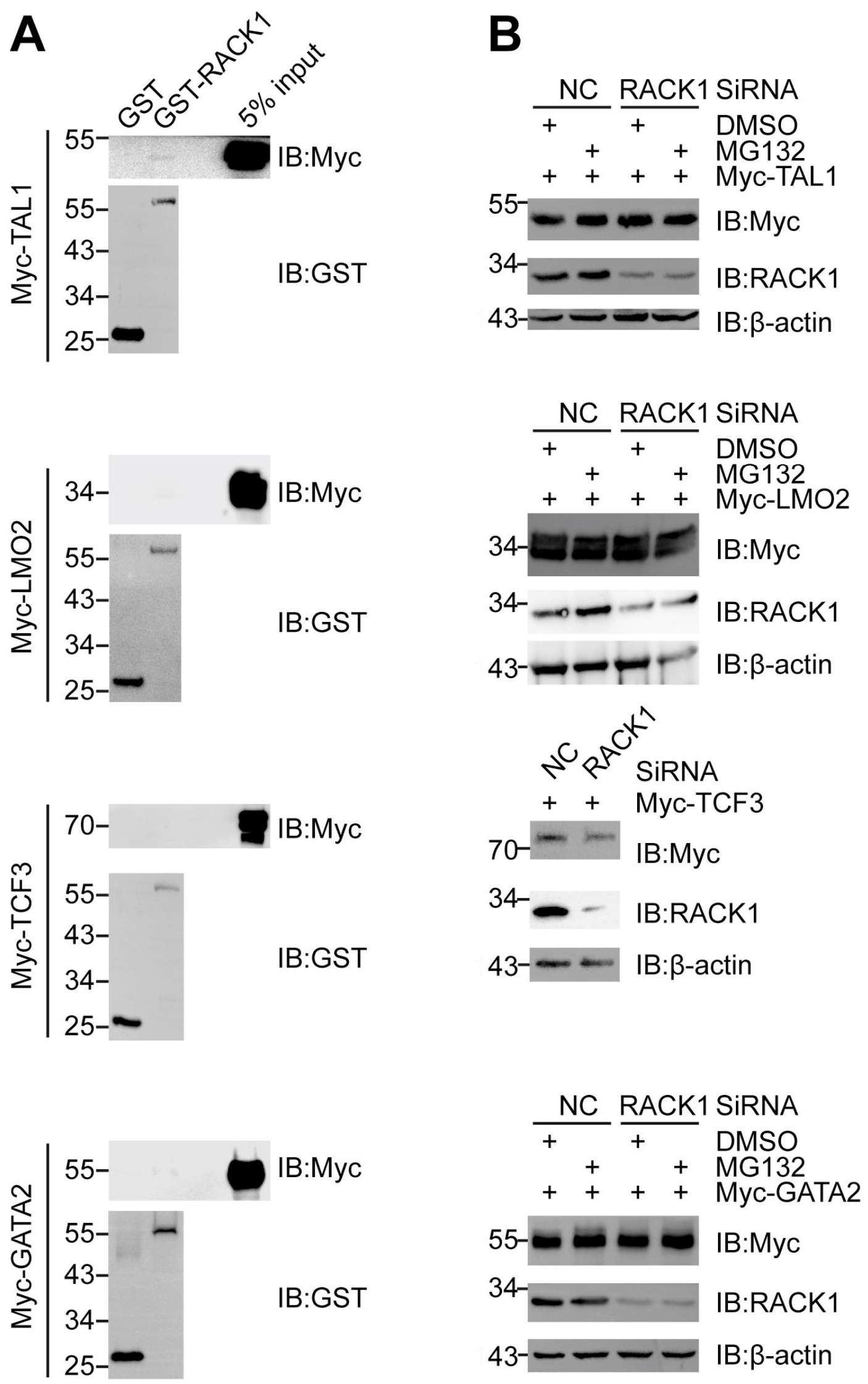

Fig. S11 RACK1 does not directly affect the protein levels of TAL1, LMO2, TCF3, and GATA2. (**A**) GST or GST-RACK1 bound to glutathione-Sepharose beads were incubated with lysates of HEK-293T cells expressing Myc-tagged TAL1, LMO2, TCF3, and GATA2. Precipitates were subjected to IB with the indicated antibodies. (**B**) At 42 h after transfection with the indicated siRNA and mammalian expression vector, HEK-293T cells were treated with 20 μM MG132 or DMSO of an equal volume for 6 h. The protein levels of Myc-tagged TAL1, LMO2, TCF3, and GATA2 were examined by IB.

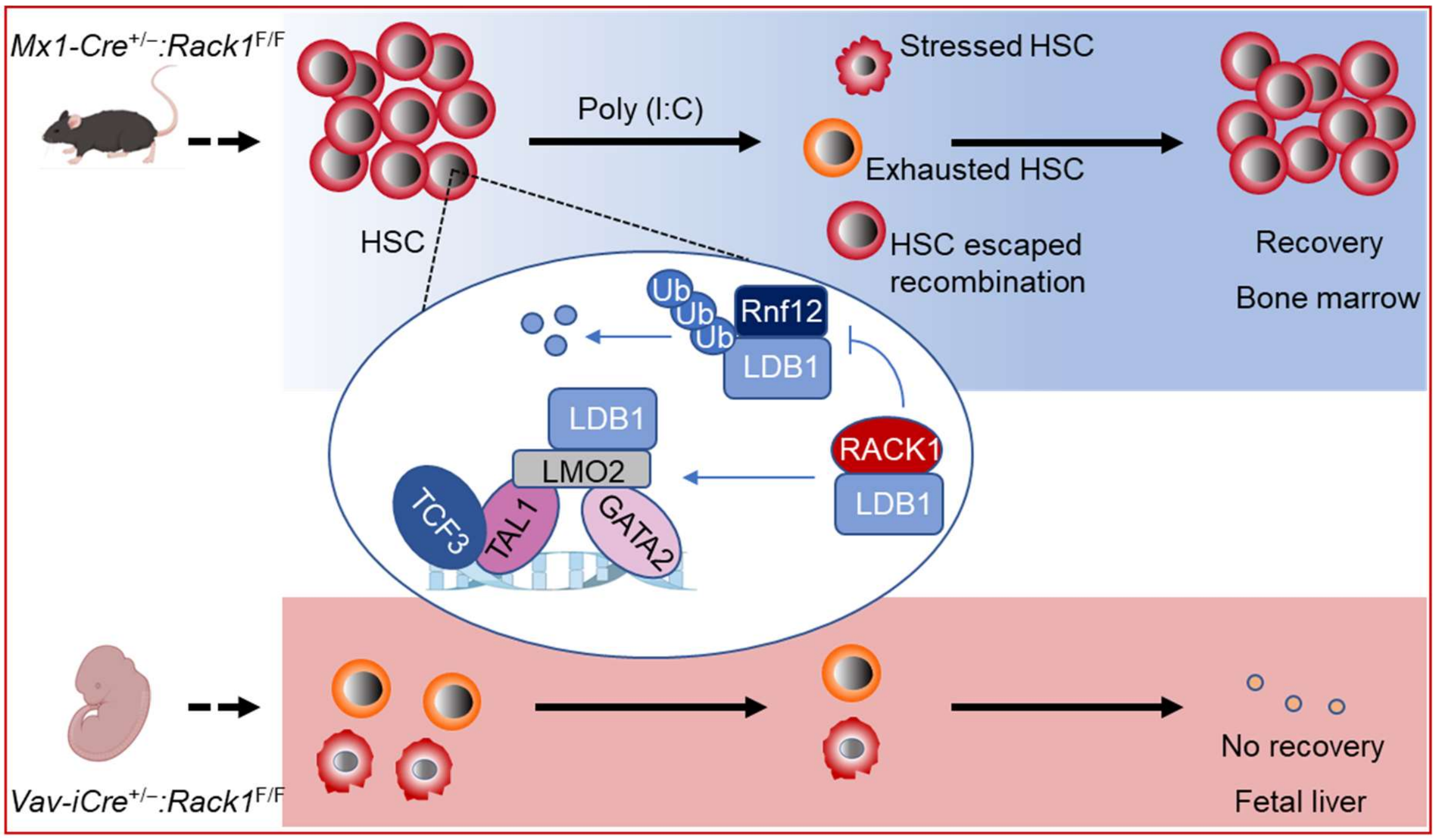

Fig. S12 A schematic presentation of the role of RACK1 in fetal and adult hematopoiesis.
