# Supplementary material for: RACK1 maintains mouse hematopoietic stem cells by directly binding to and stabilizing LDB1
Source: Cell Death Dis. 2026 Aug 9;17(1):943. doi: 10.1038/s41419-026-09177-z (PMC13737179; doi:10.1038/s41419-026-09177-z)

Source Fig. 2A

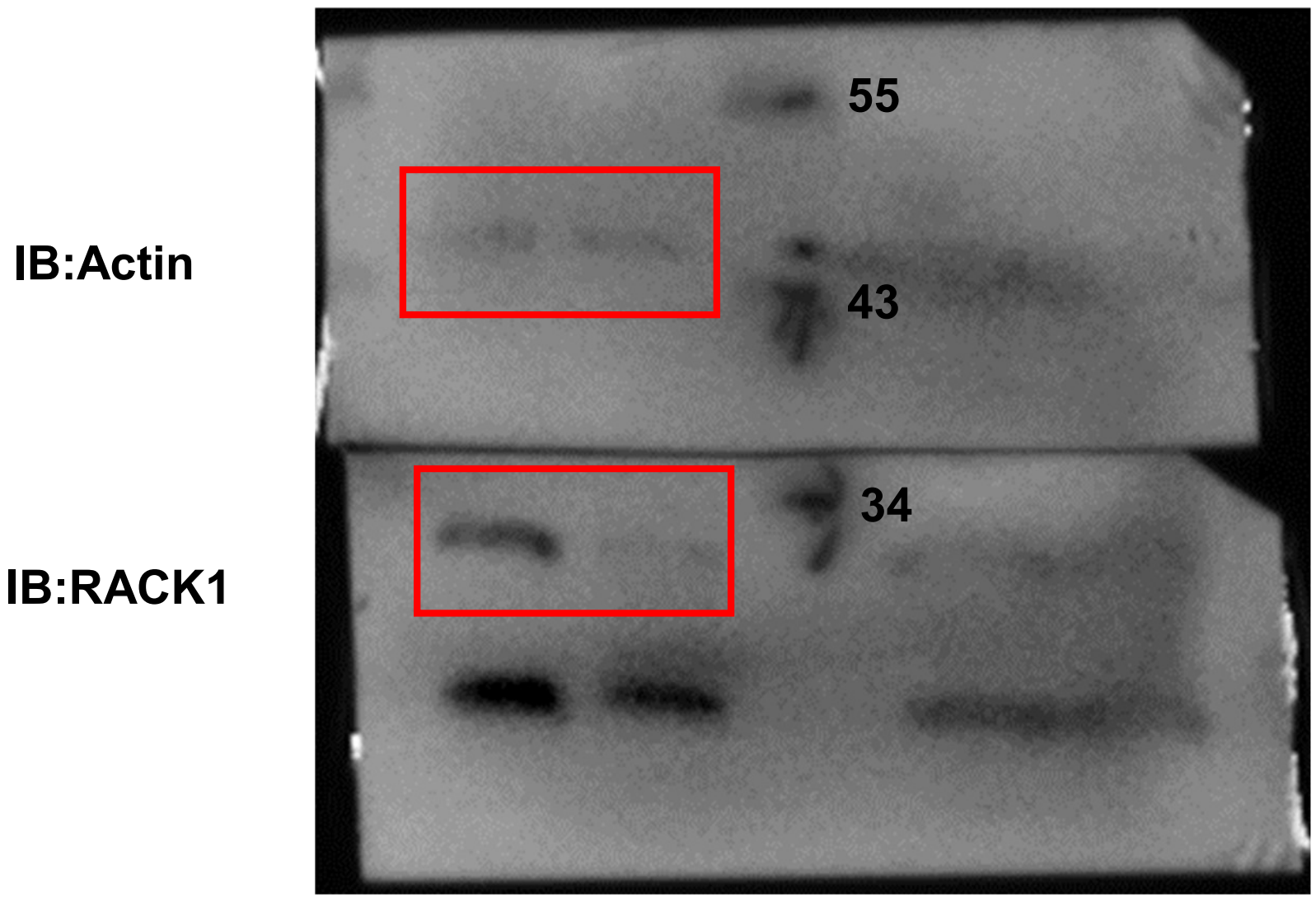

Source Fig. 7A

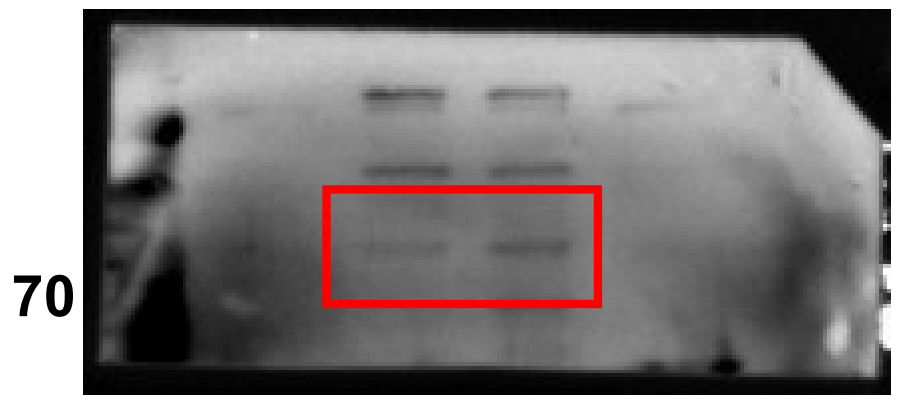

IB:TCF3

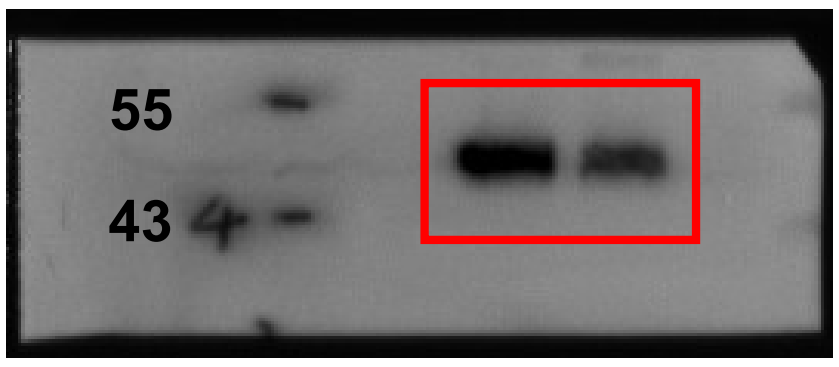

IB:TAL1

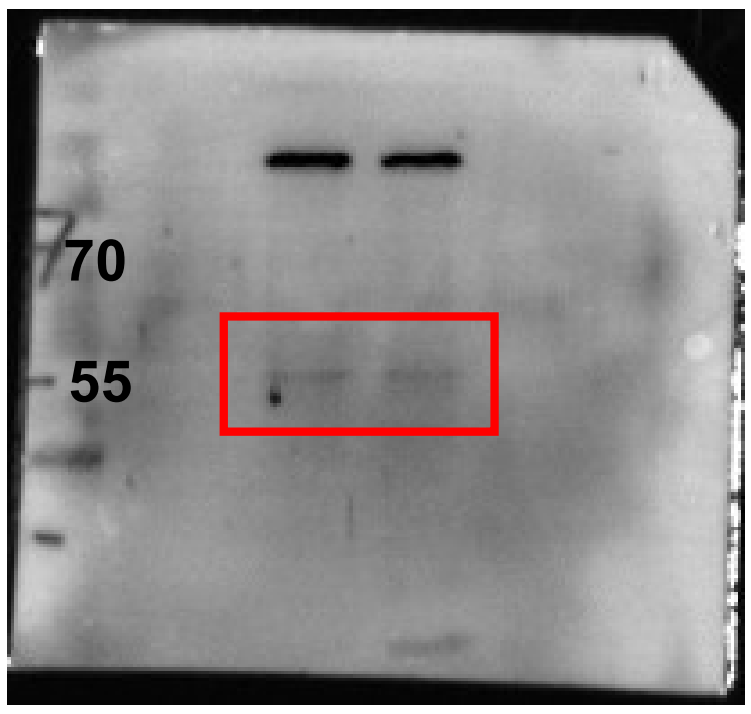

IB:GATA2

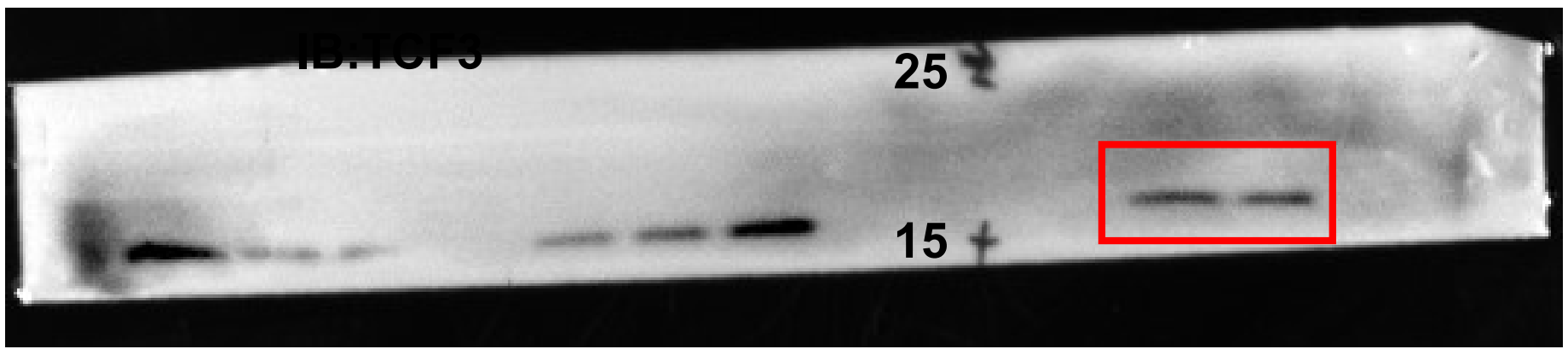

IB:LMO2

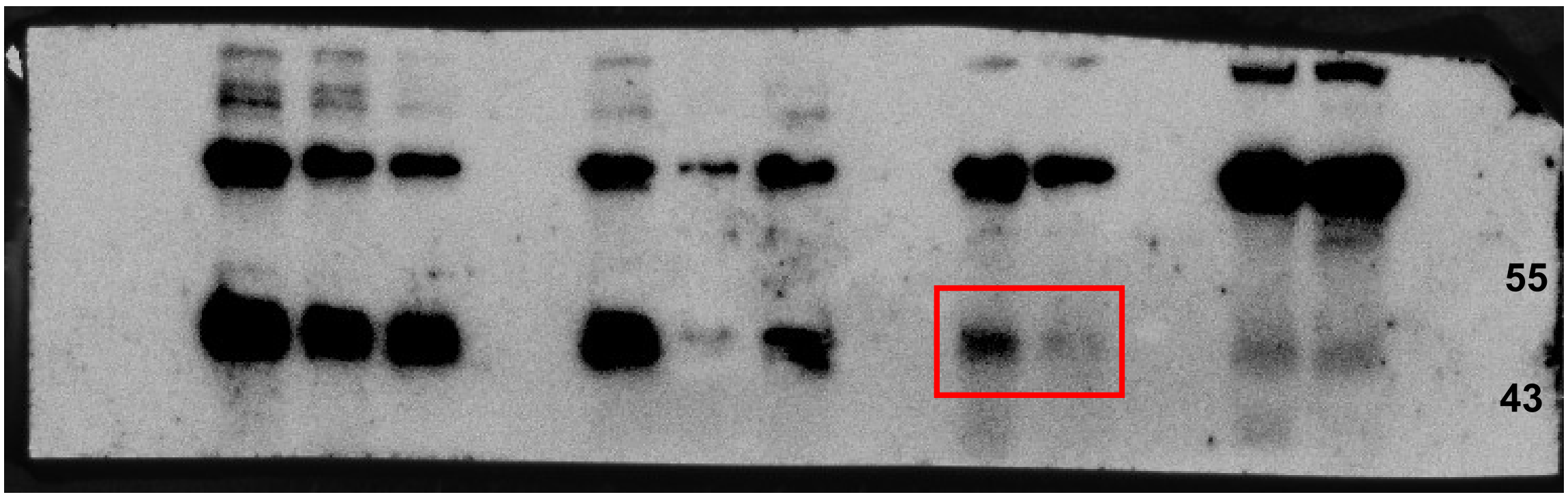

IB:LDB1

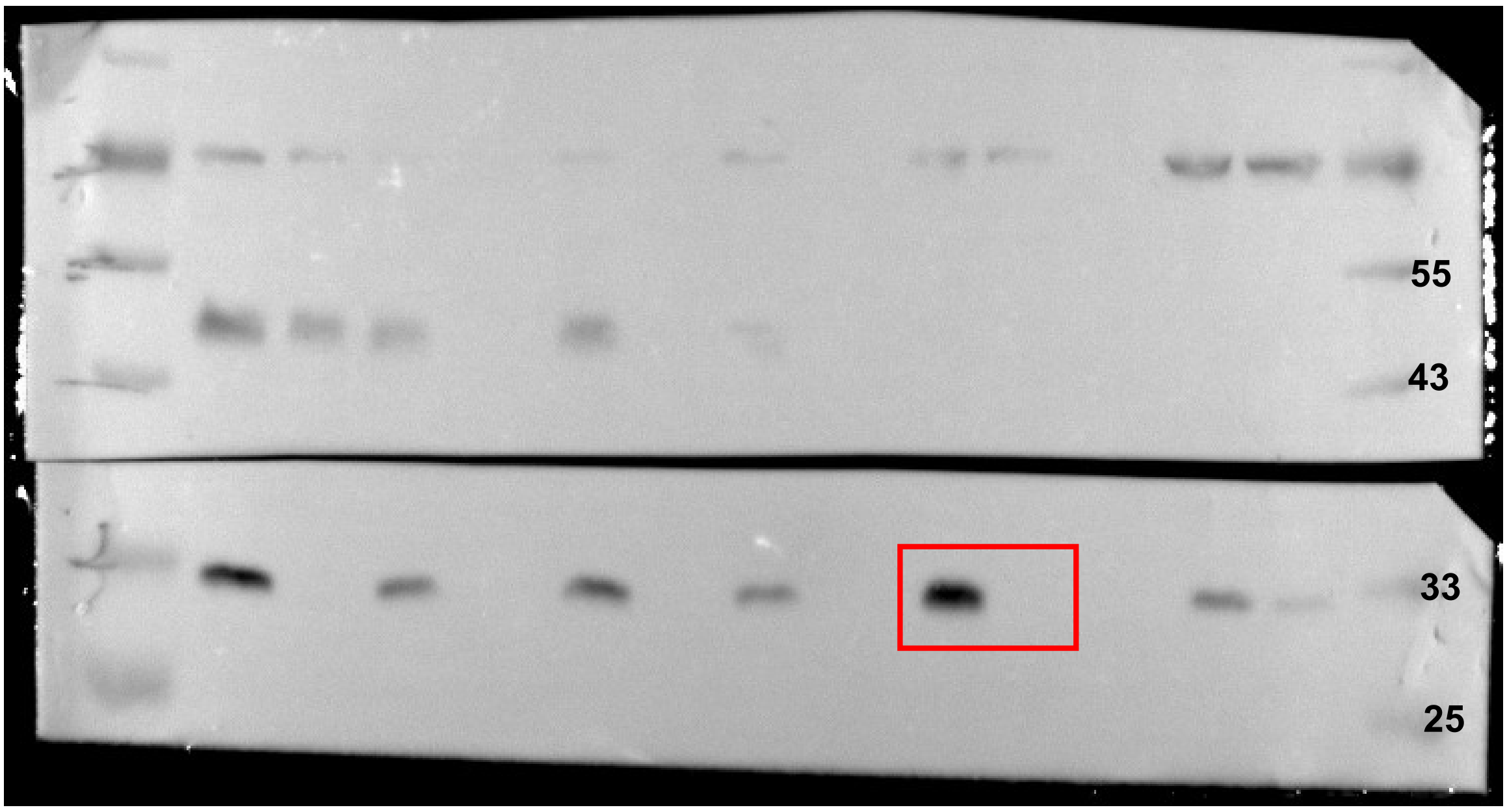

IB:LDB1

IB:RACK1

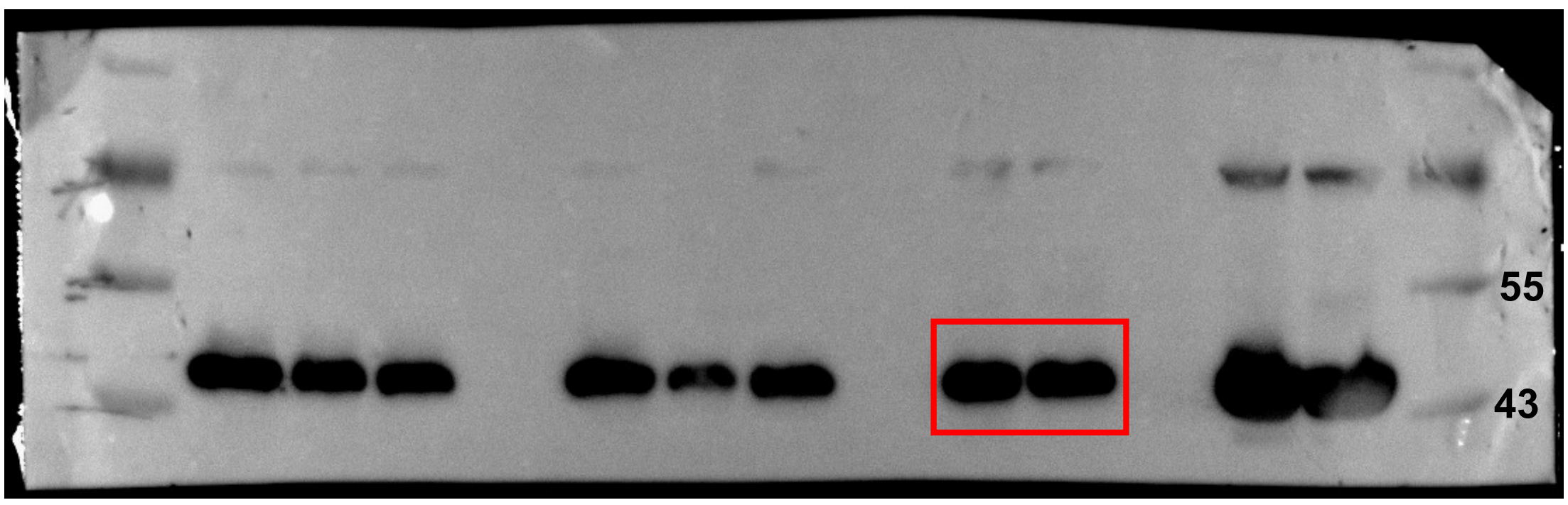

IB:β-actin

Source Fig. 7D

Jurkat

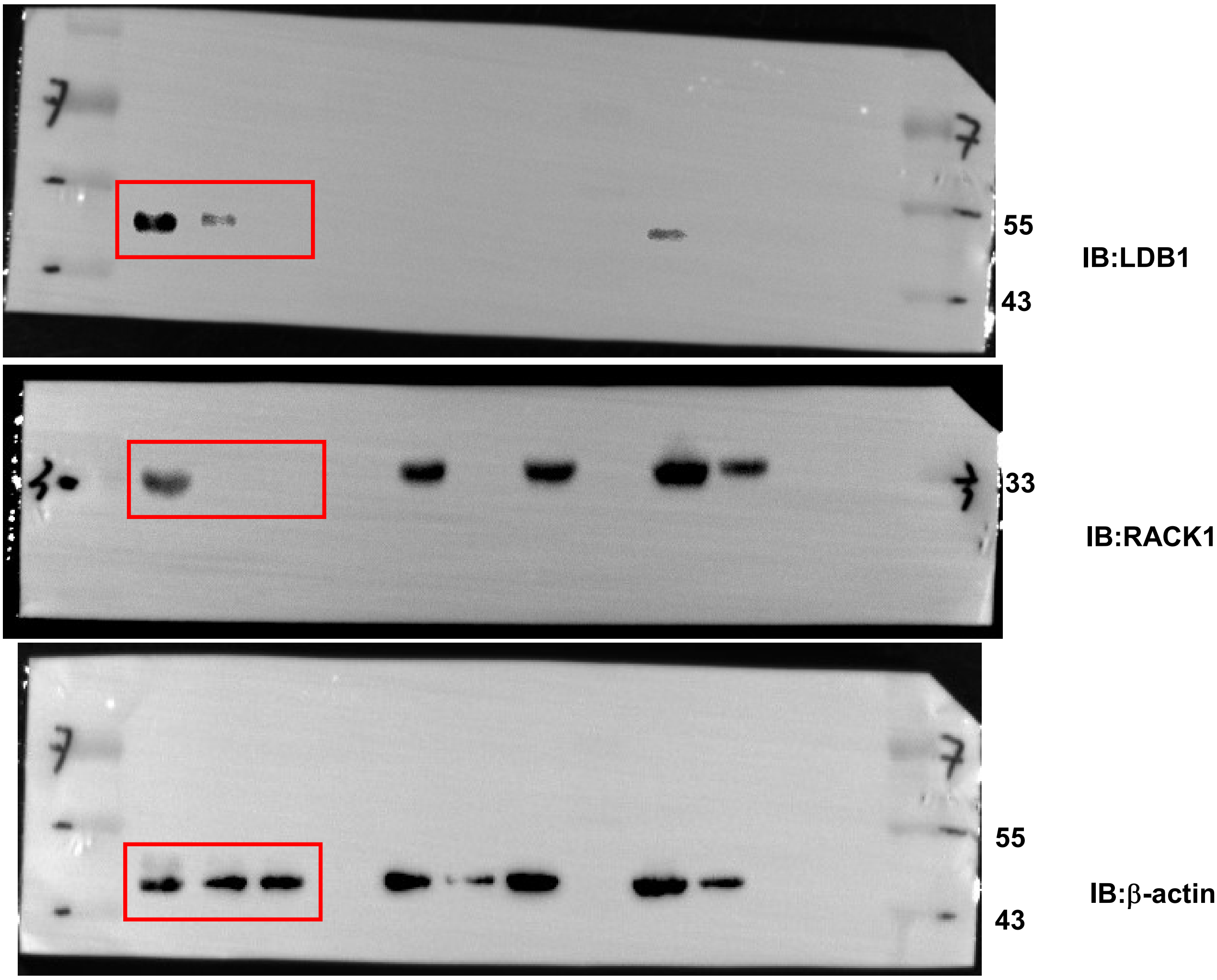

CEM

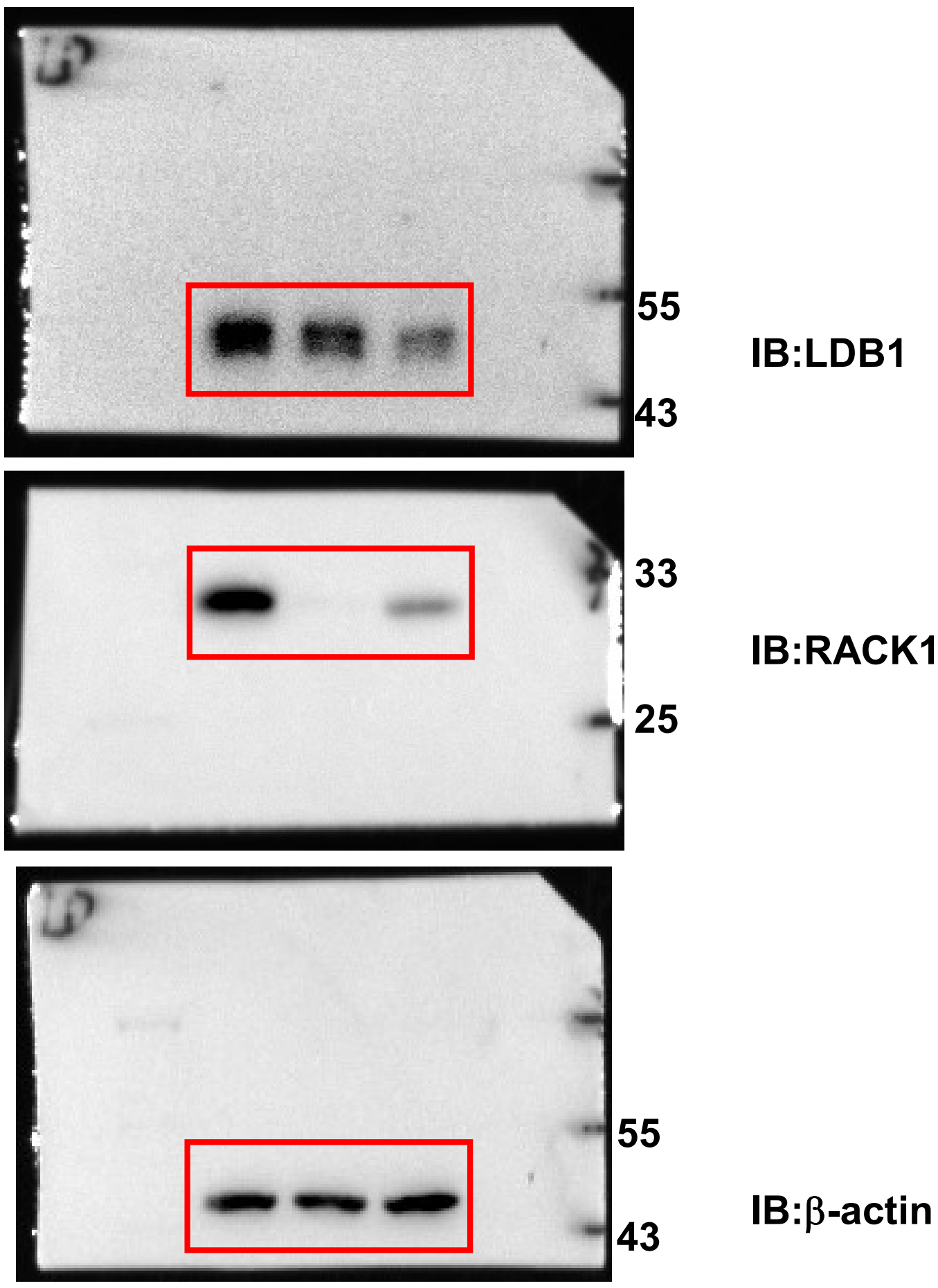

Source Fig. 7D (continued)

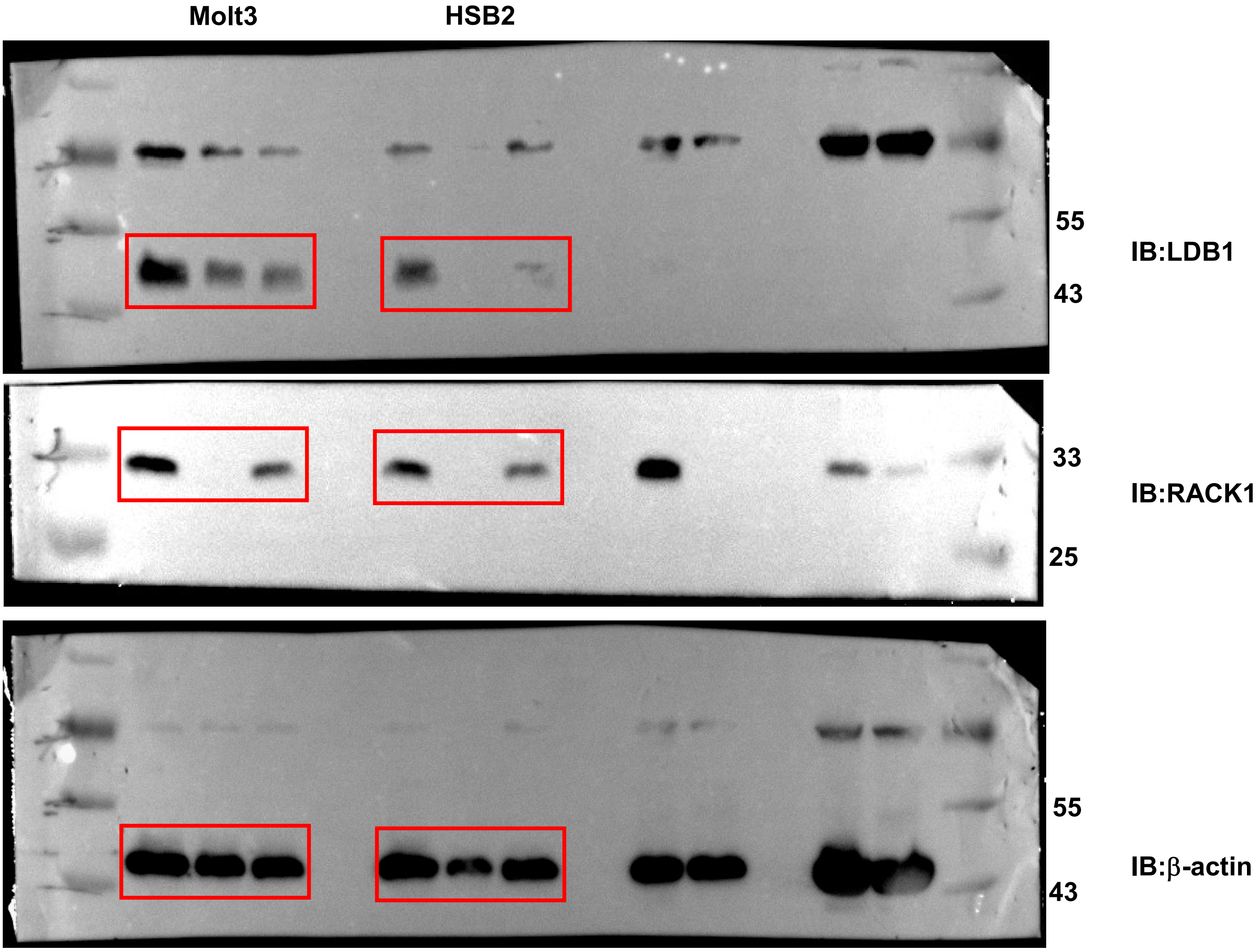

Source Fig. 8A

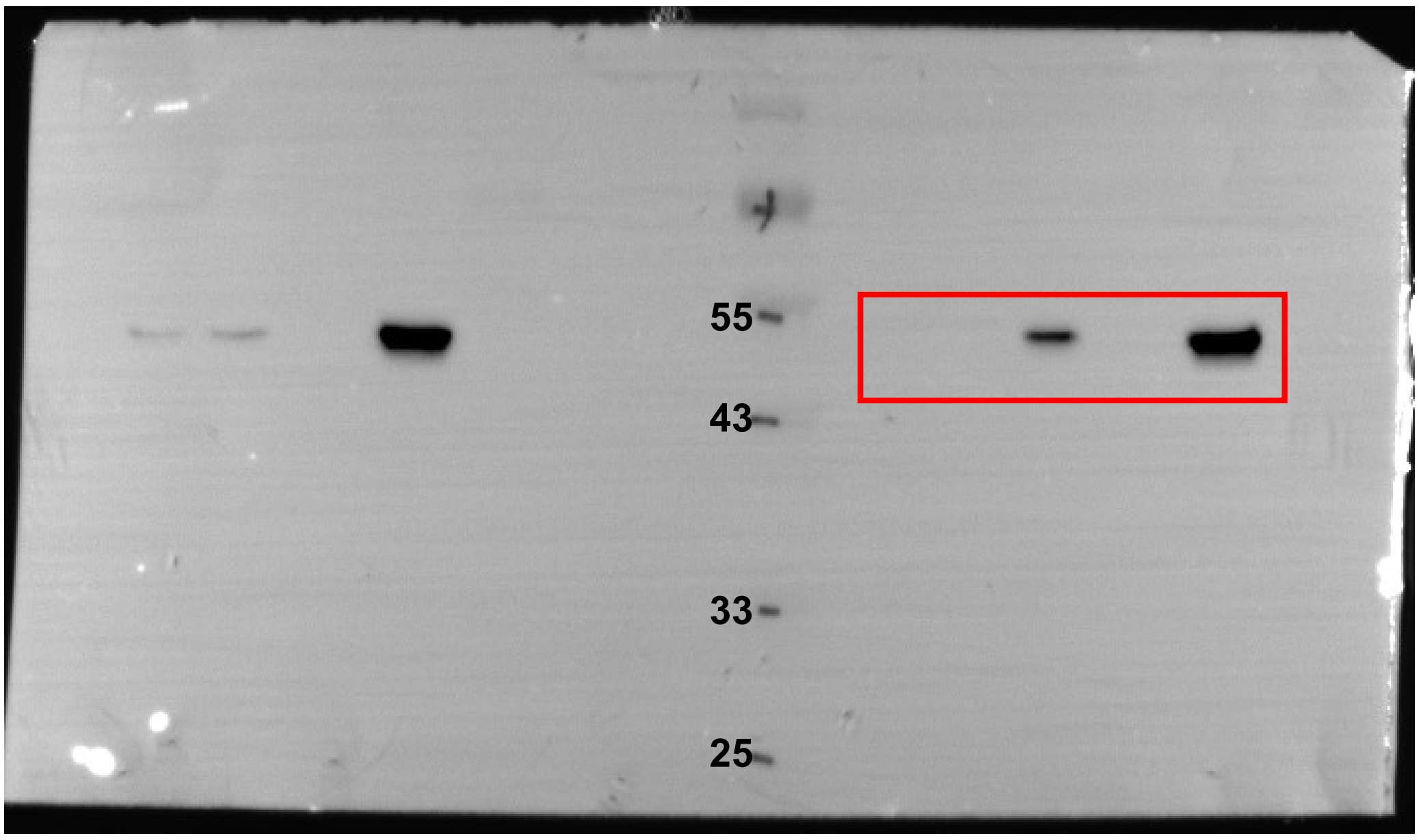

Myc-TCF3

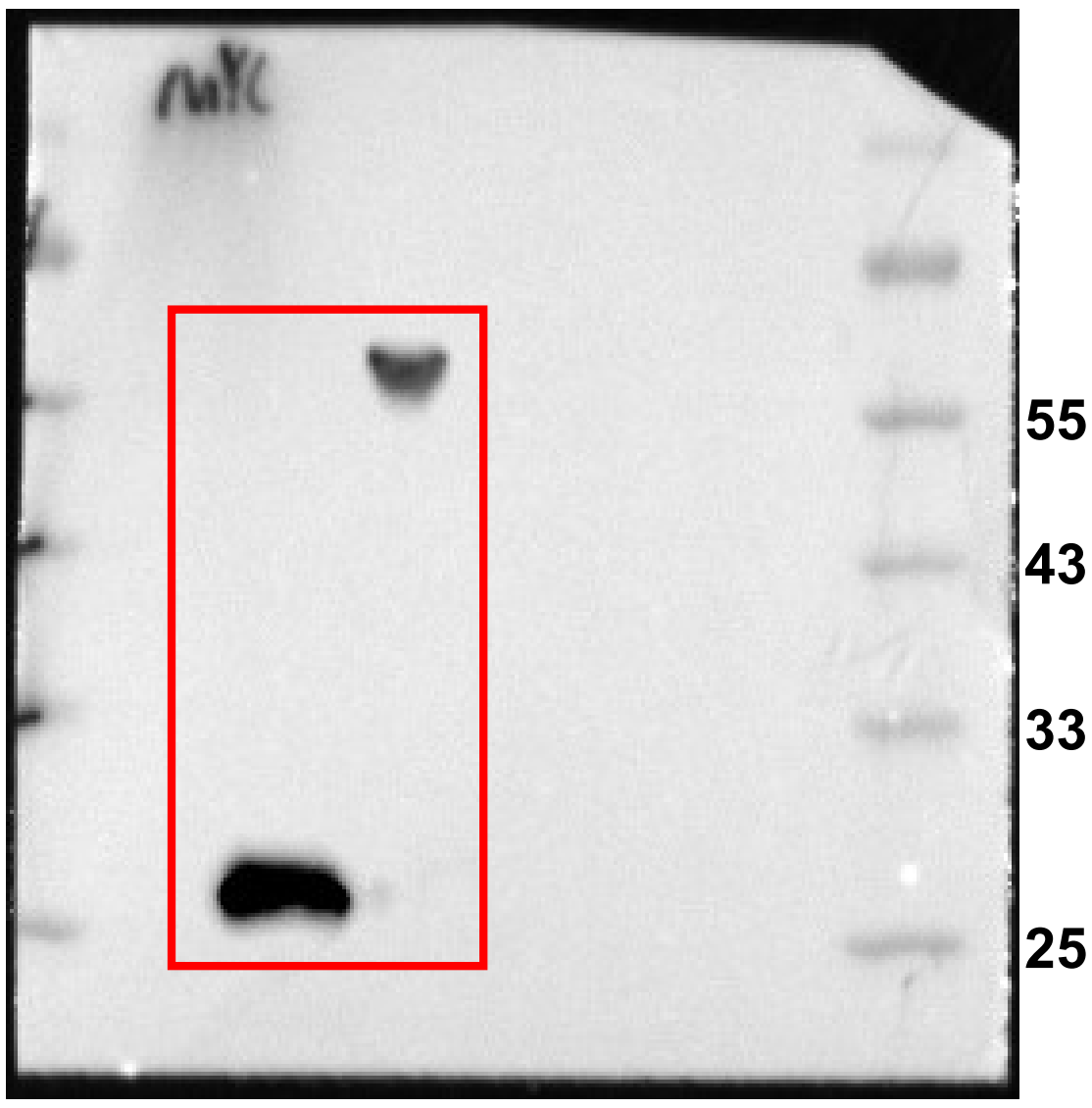

IB GST for Myc-LDB1

Source Fig. 8B

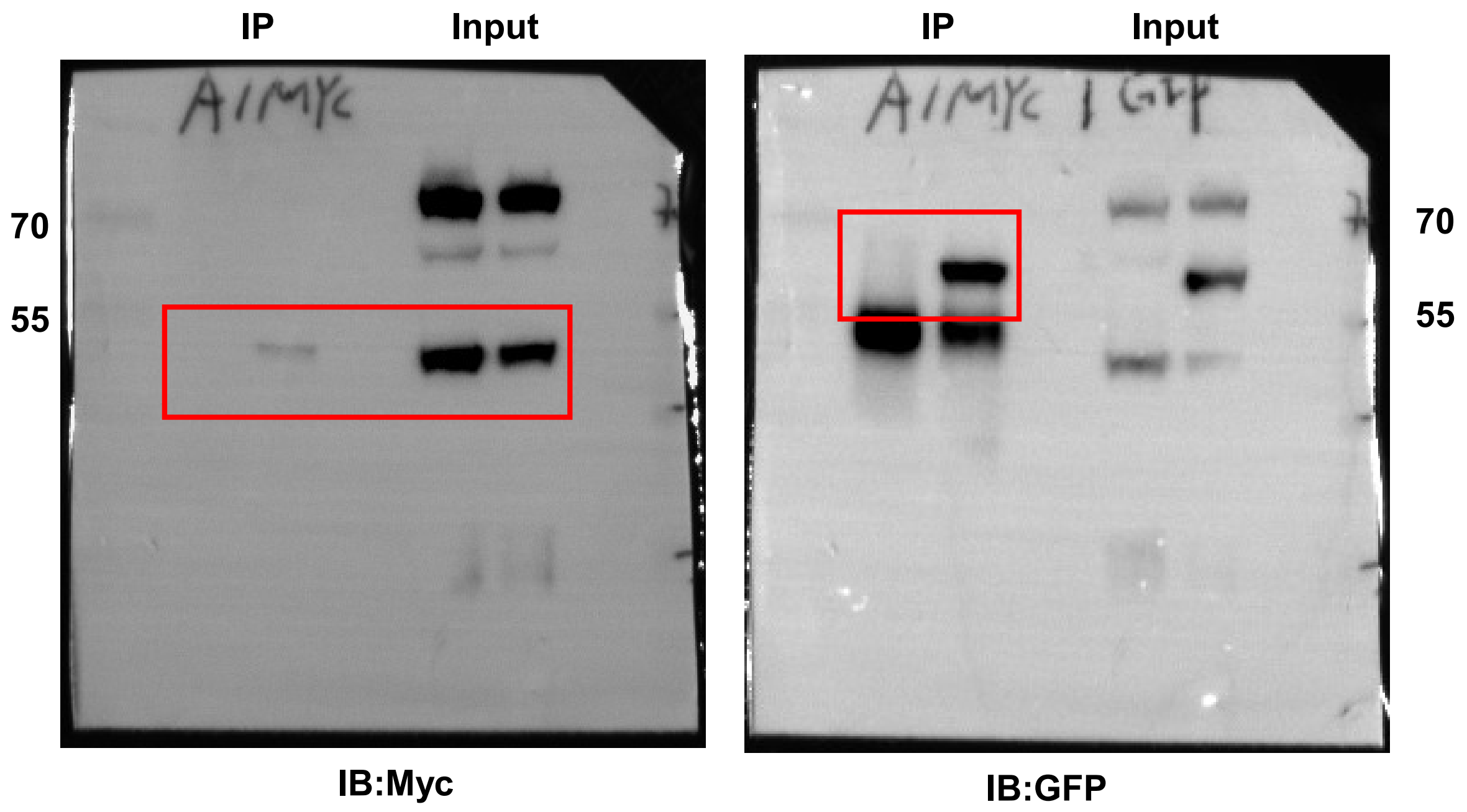

Source Fig. 8C

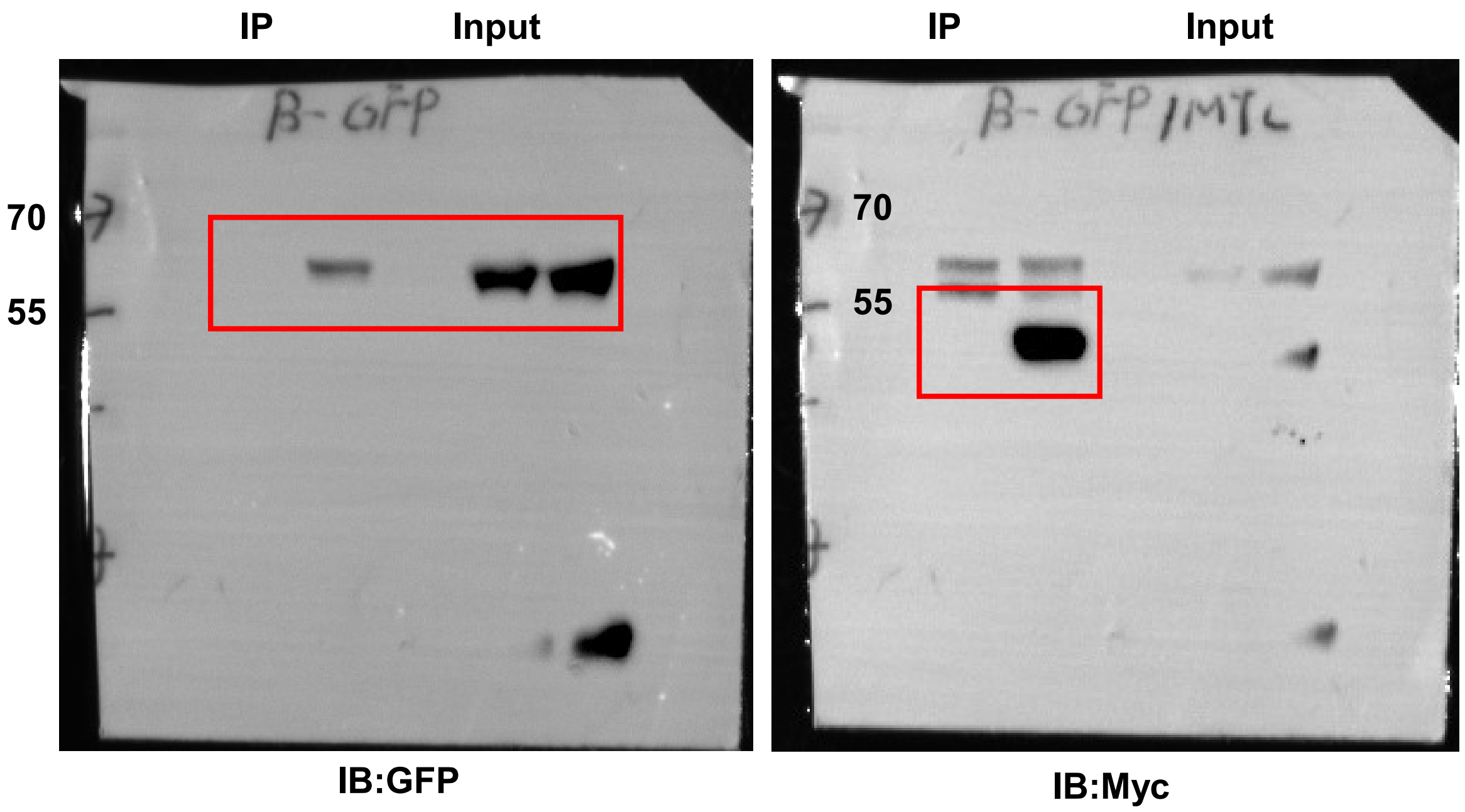

Source Fig. 8D

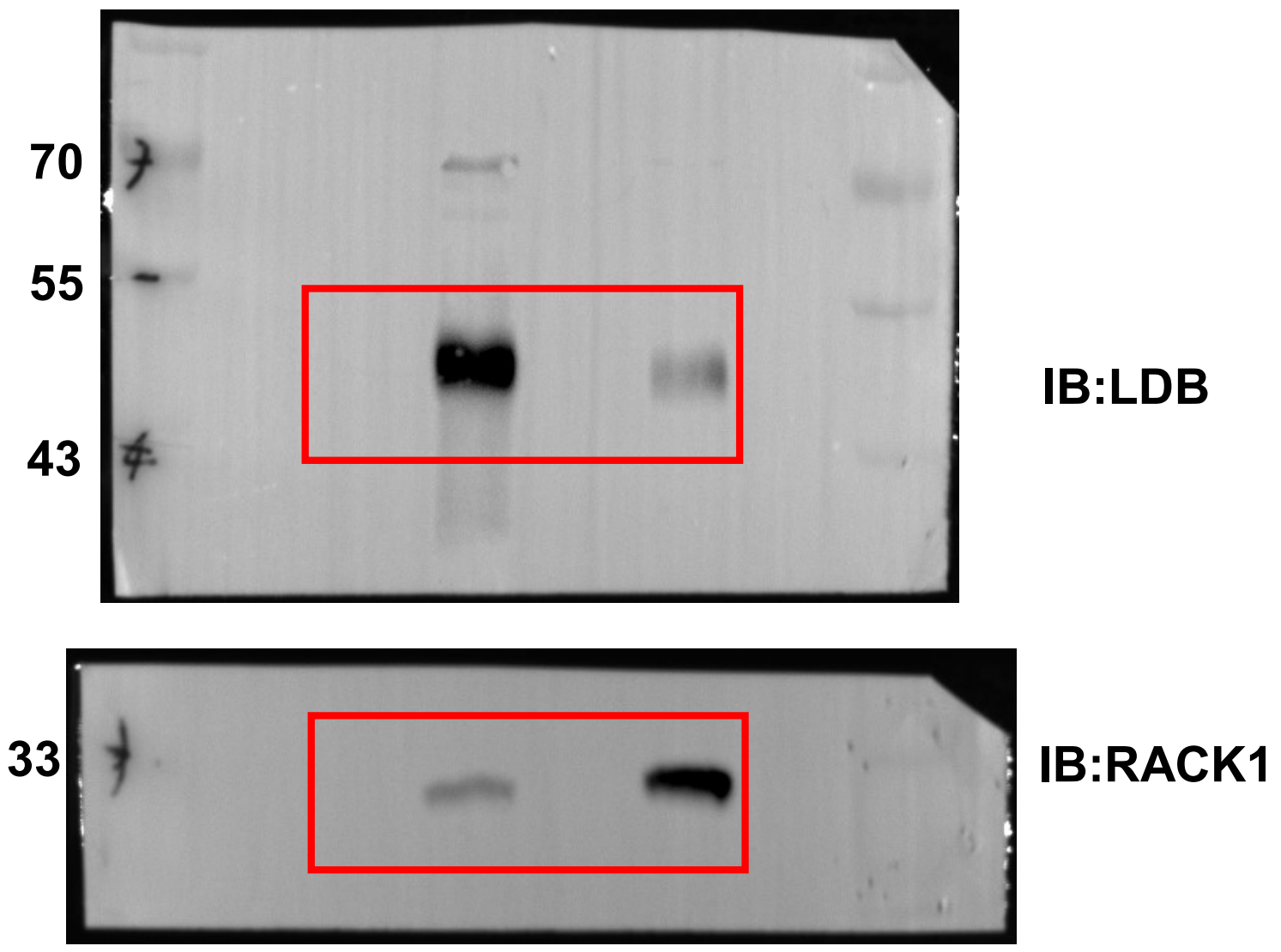

Source Fig. 8E

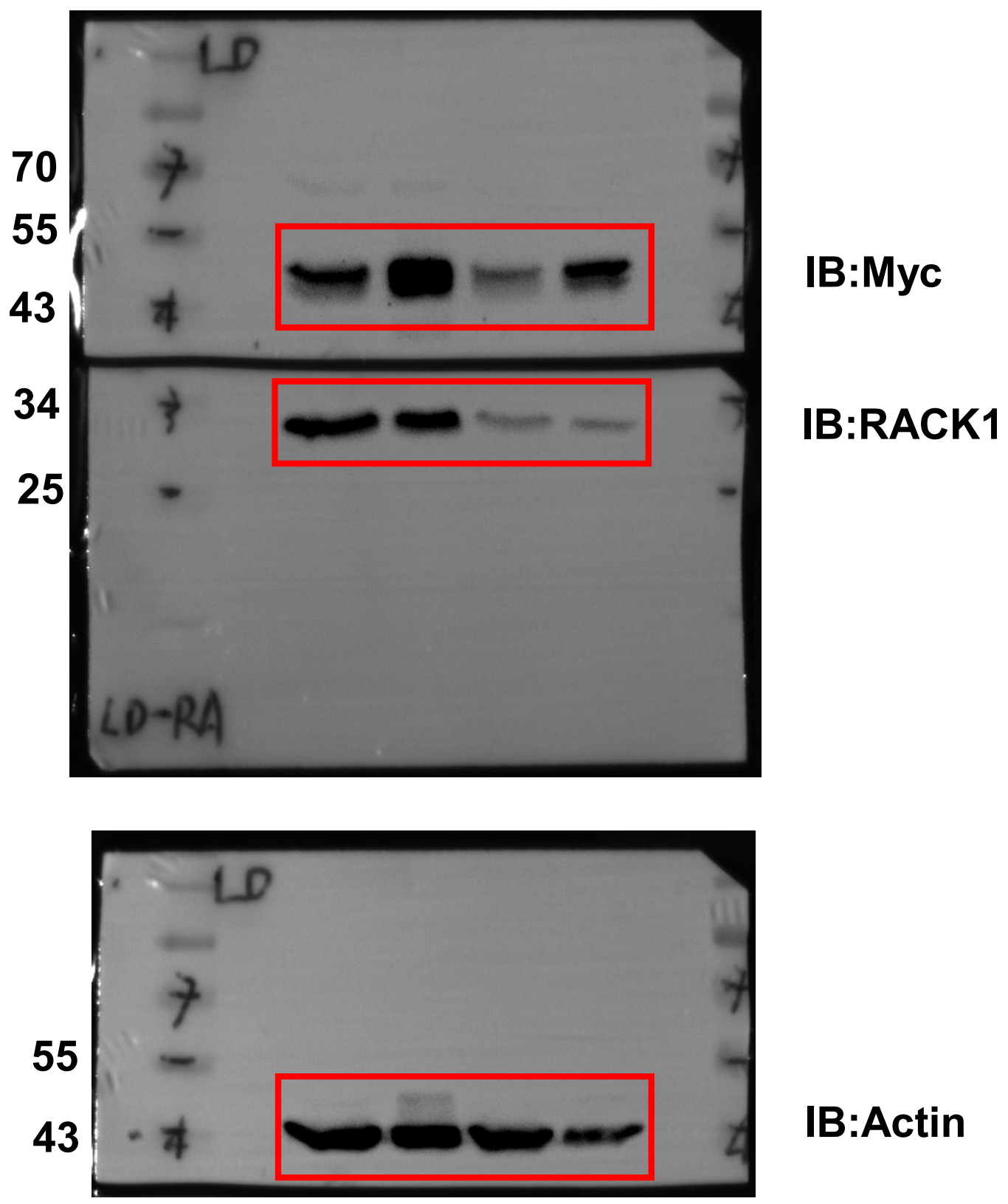

Source Fig. 8F

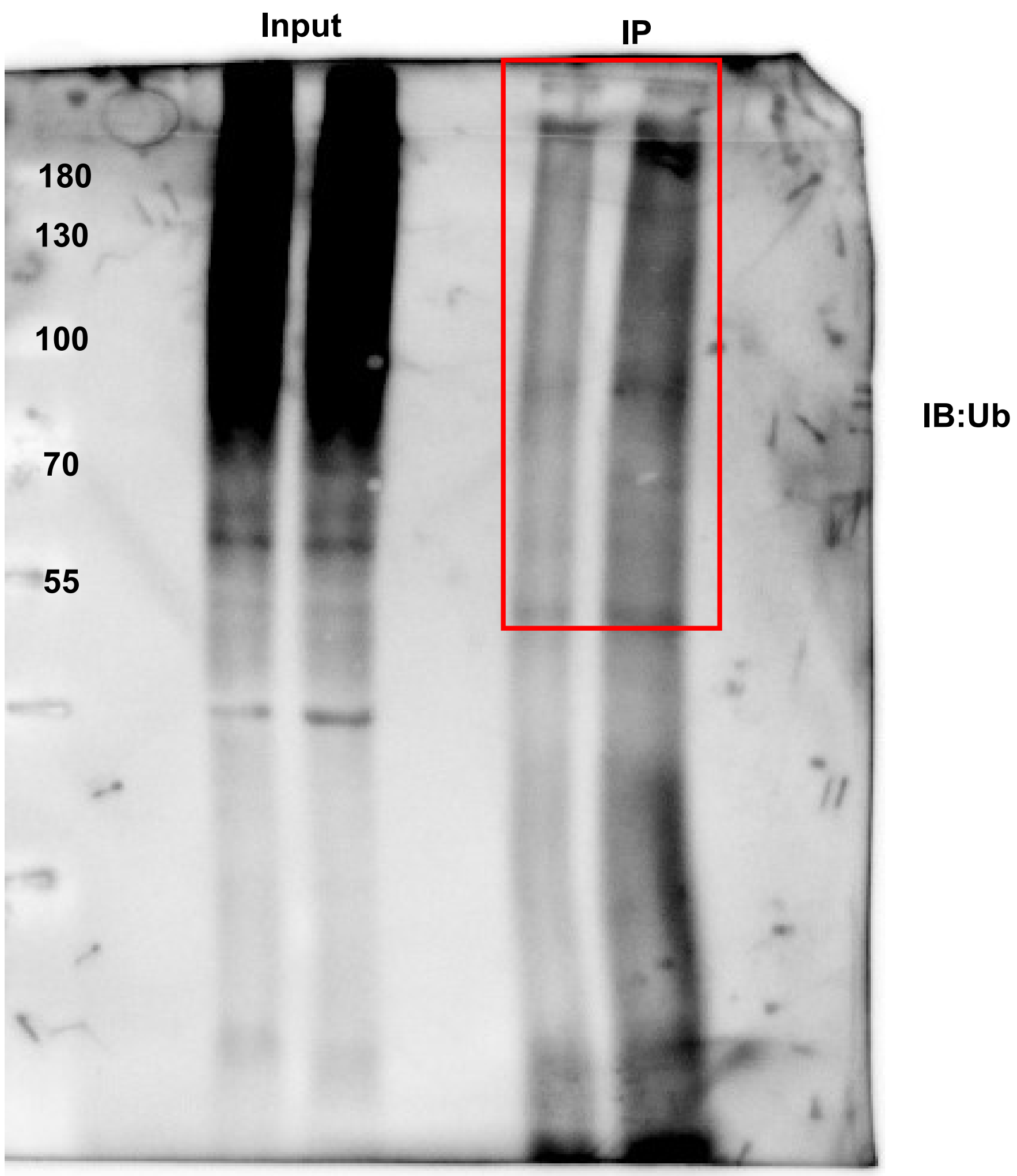

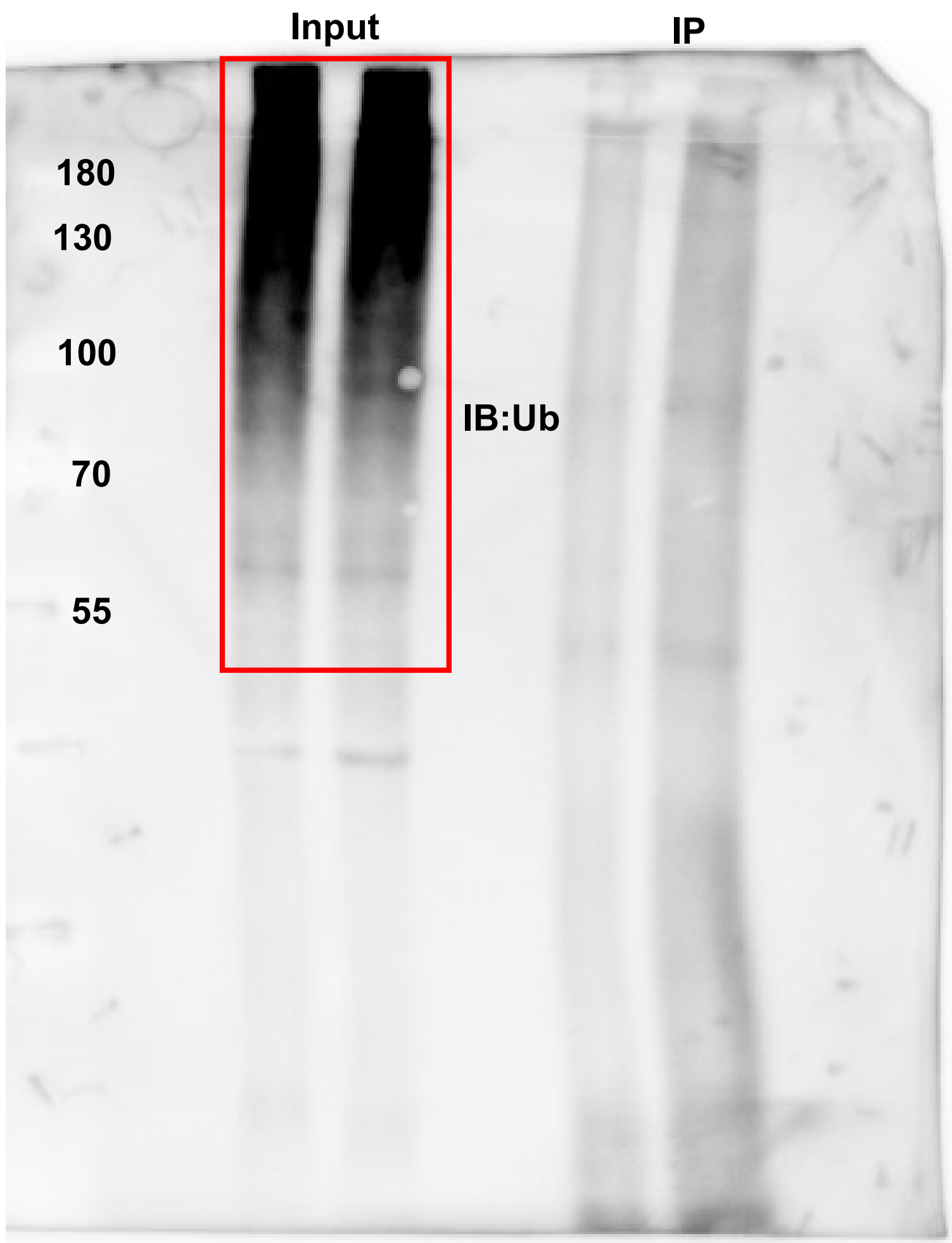

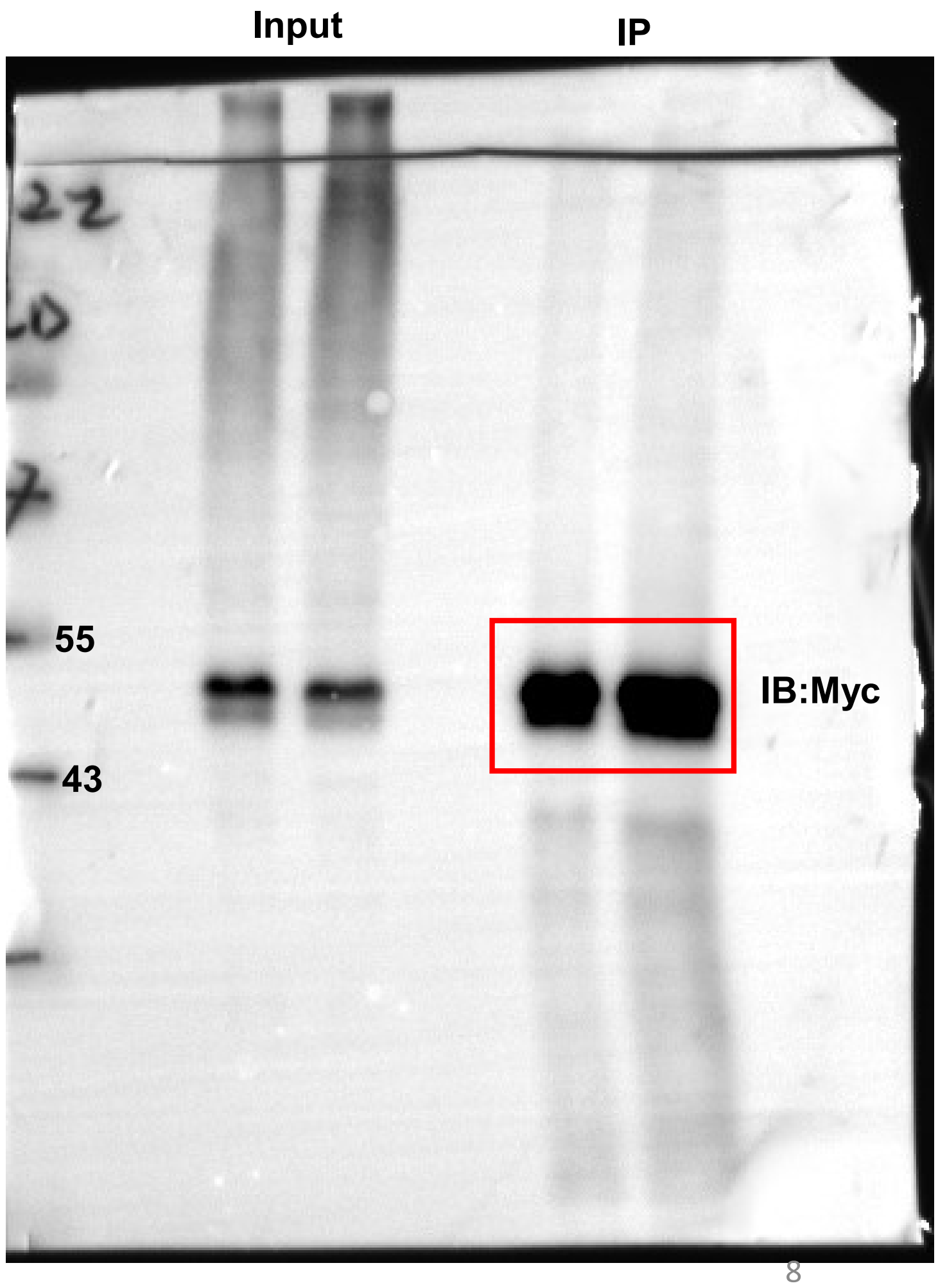

Source Fig. 8G

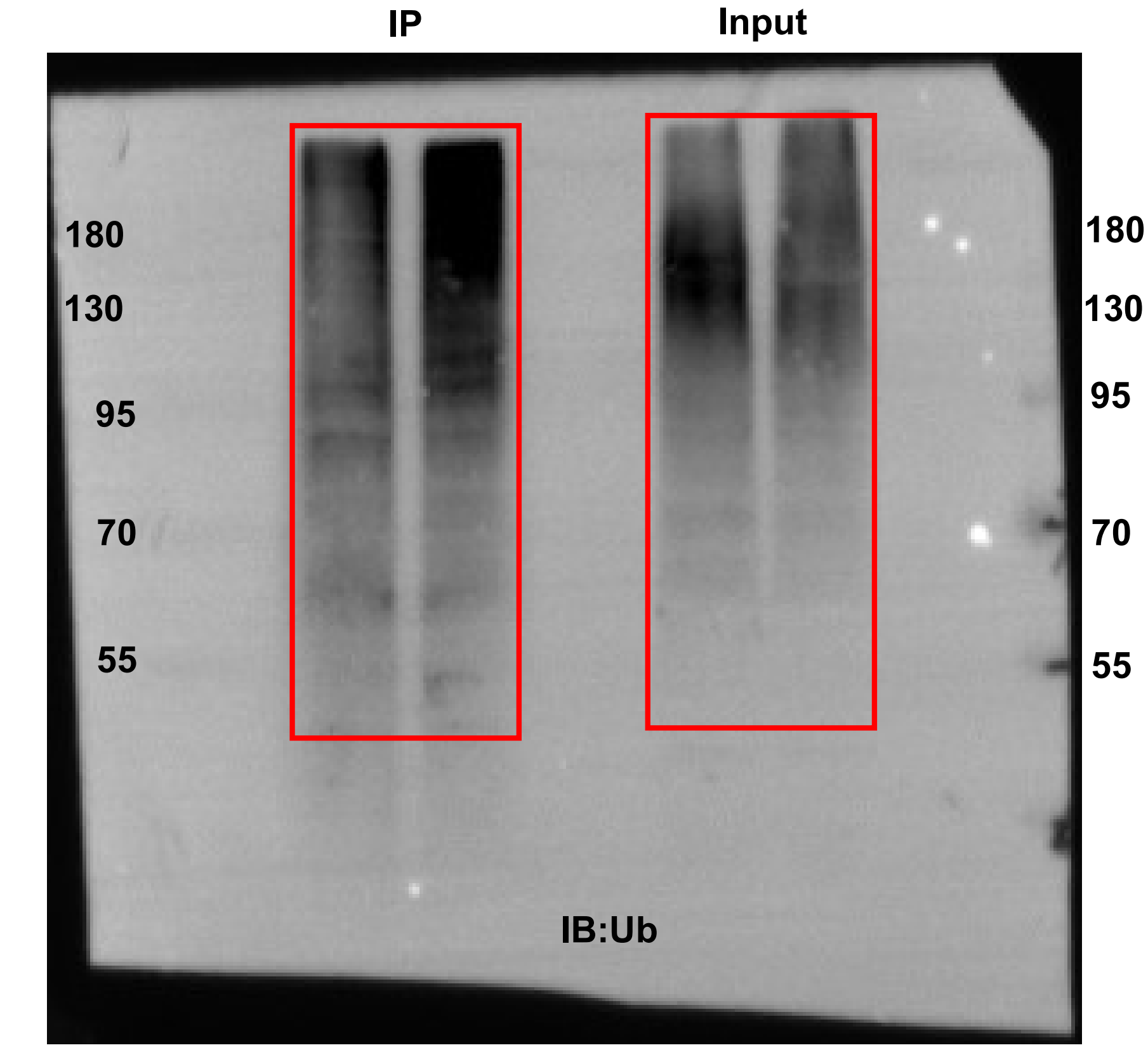

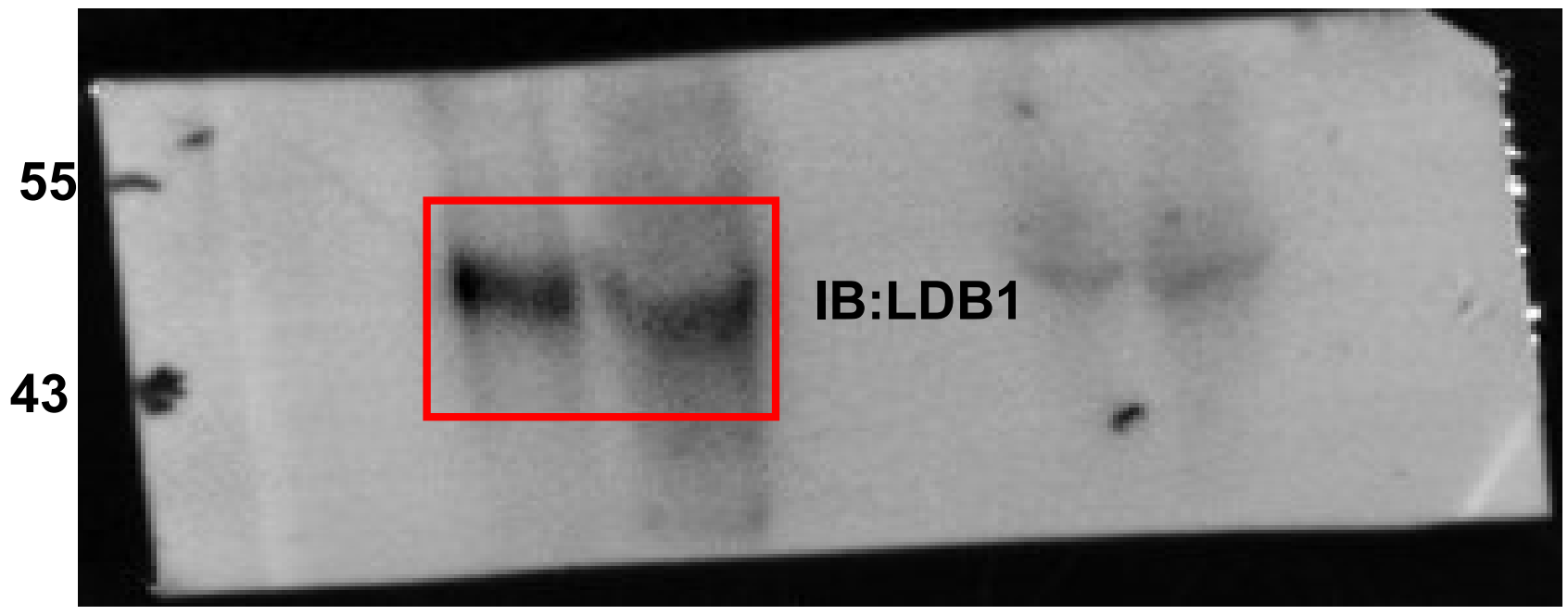

Source Fig. 8H

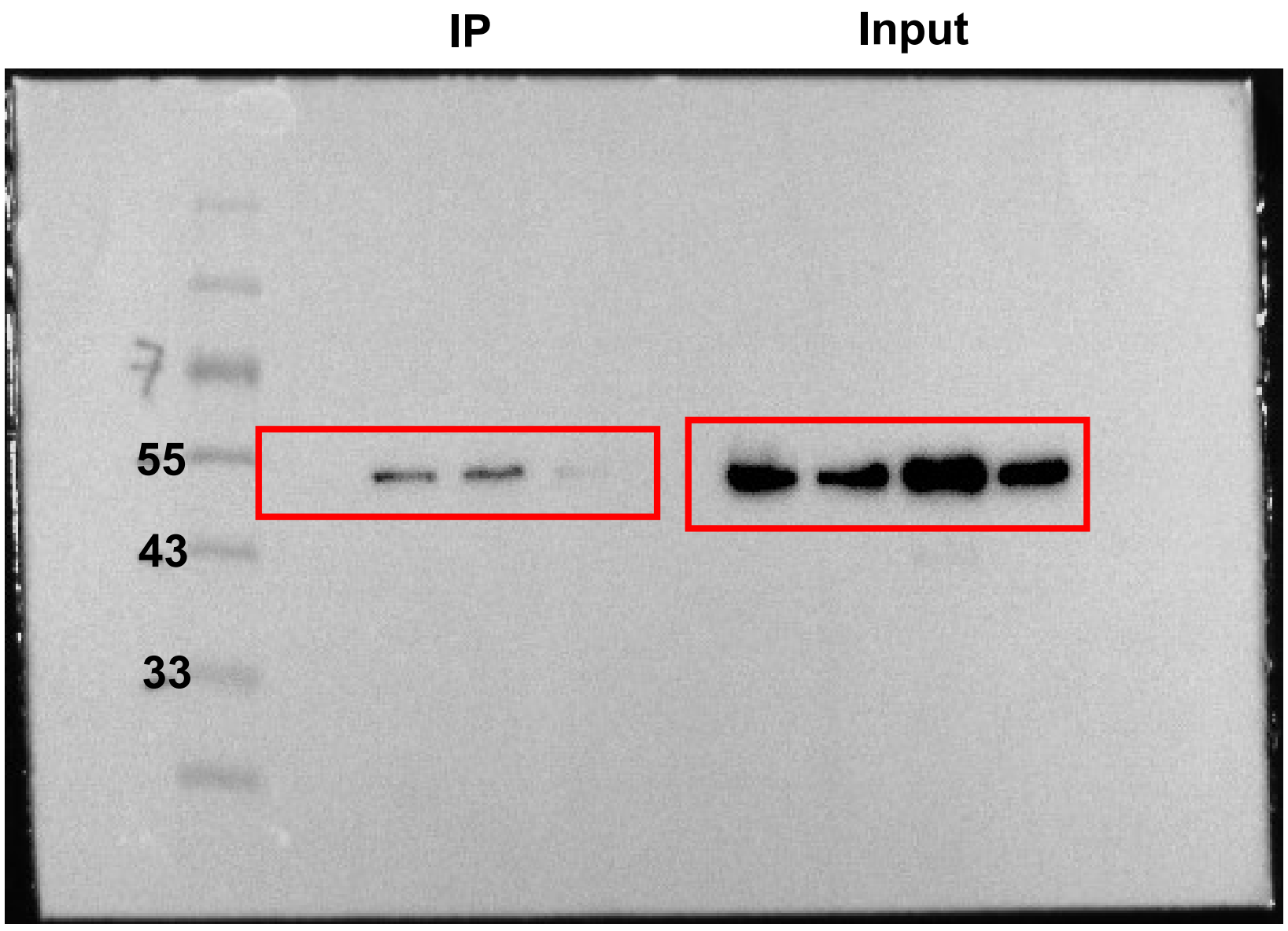

IB:Myc

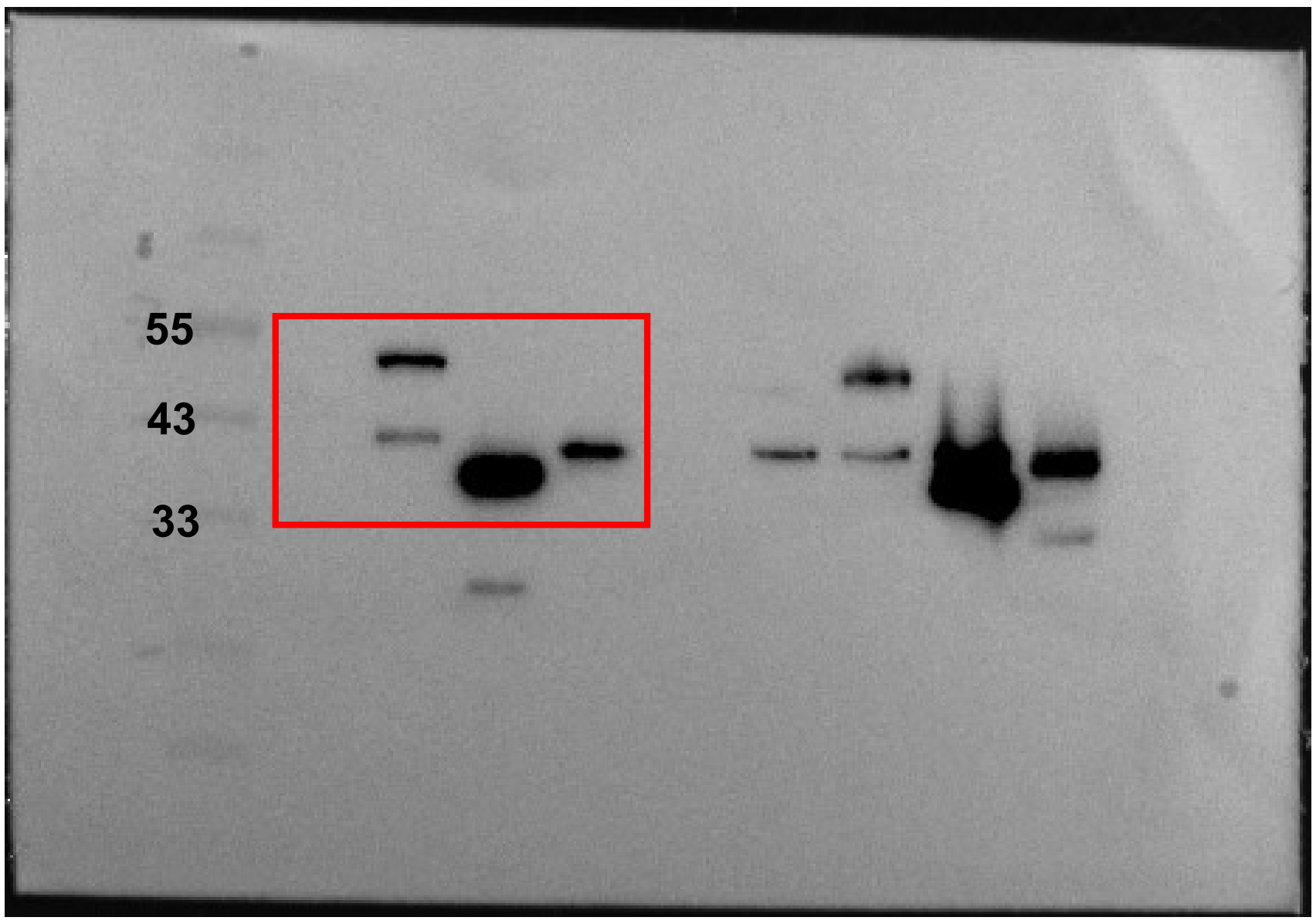

IB:GFP

Source Fig. 8I

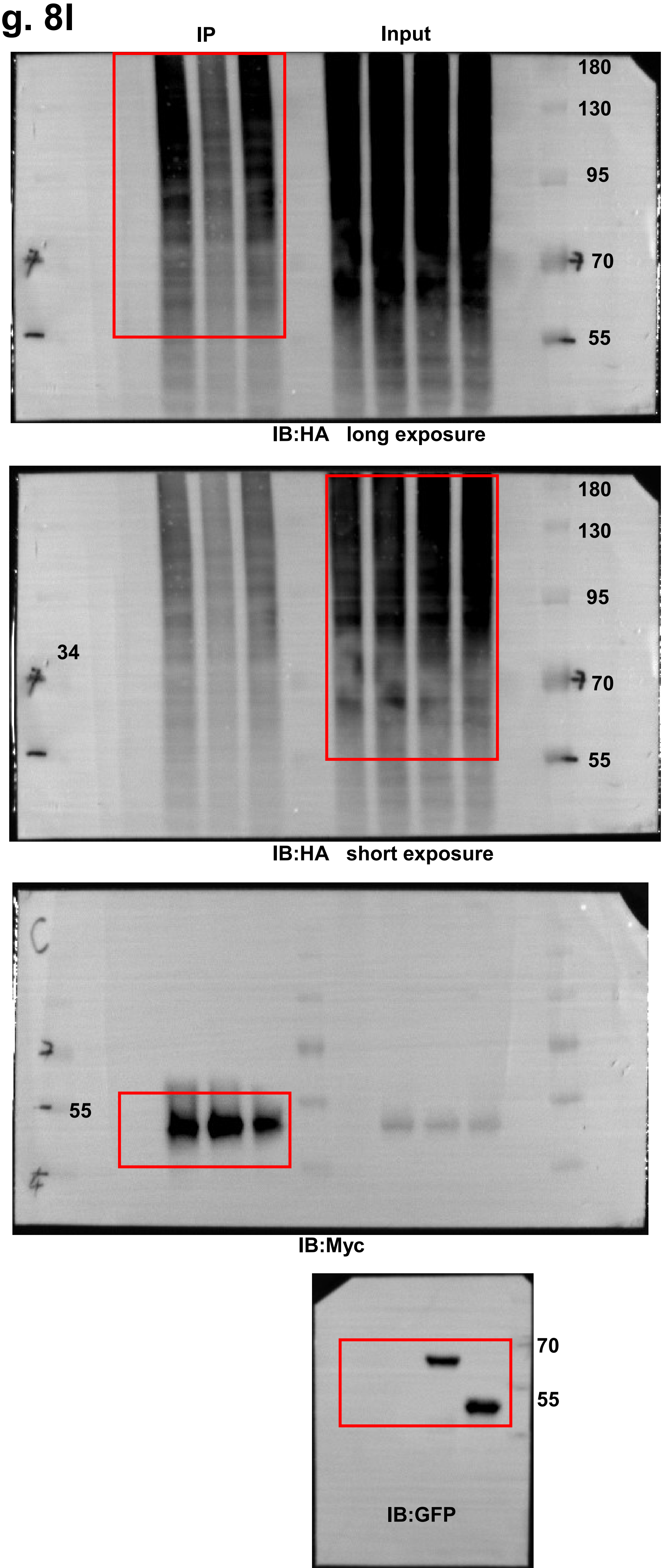

Source Fig. 8J

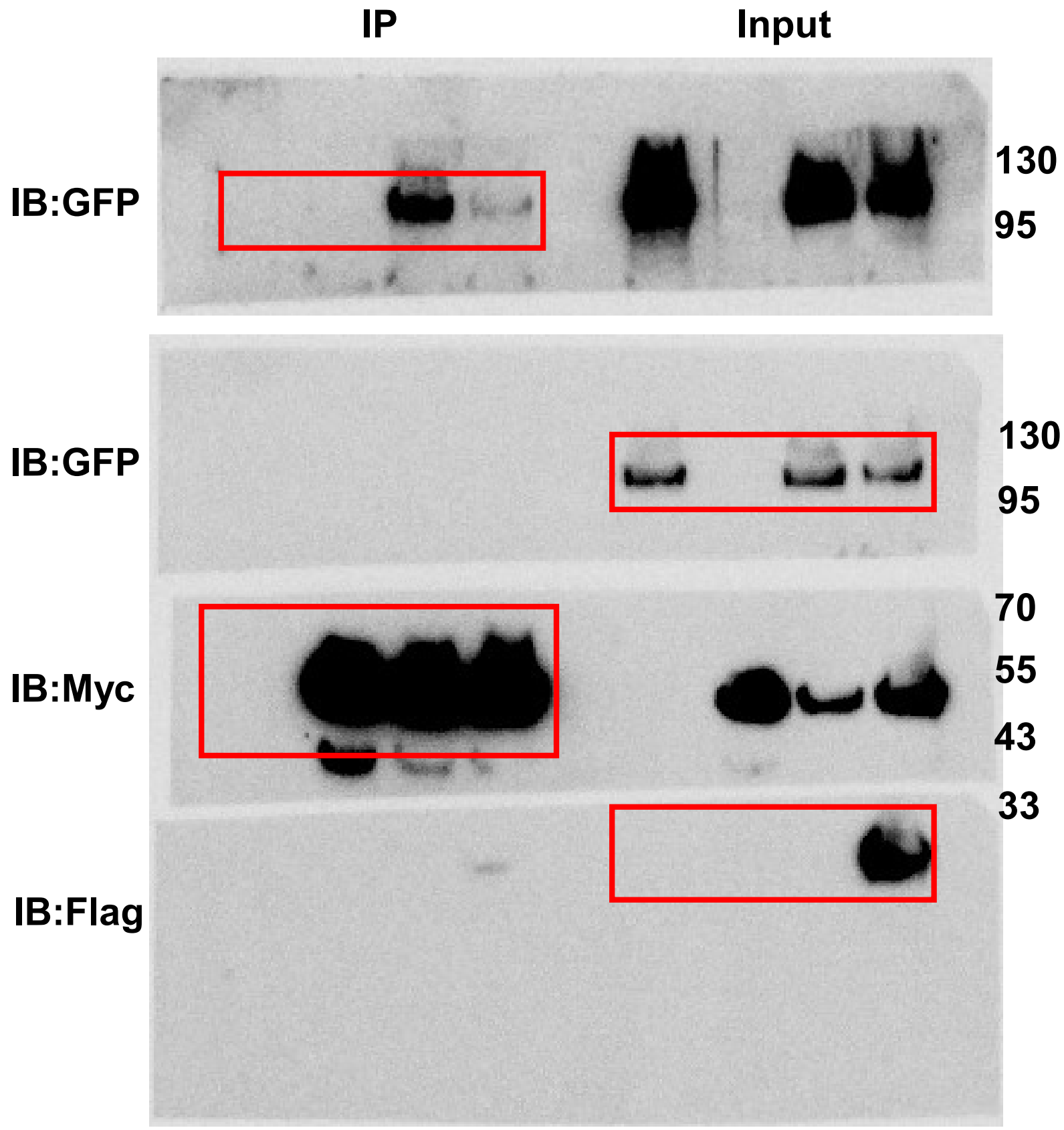

Source Fig. 8K

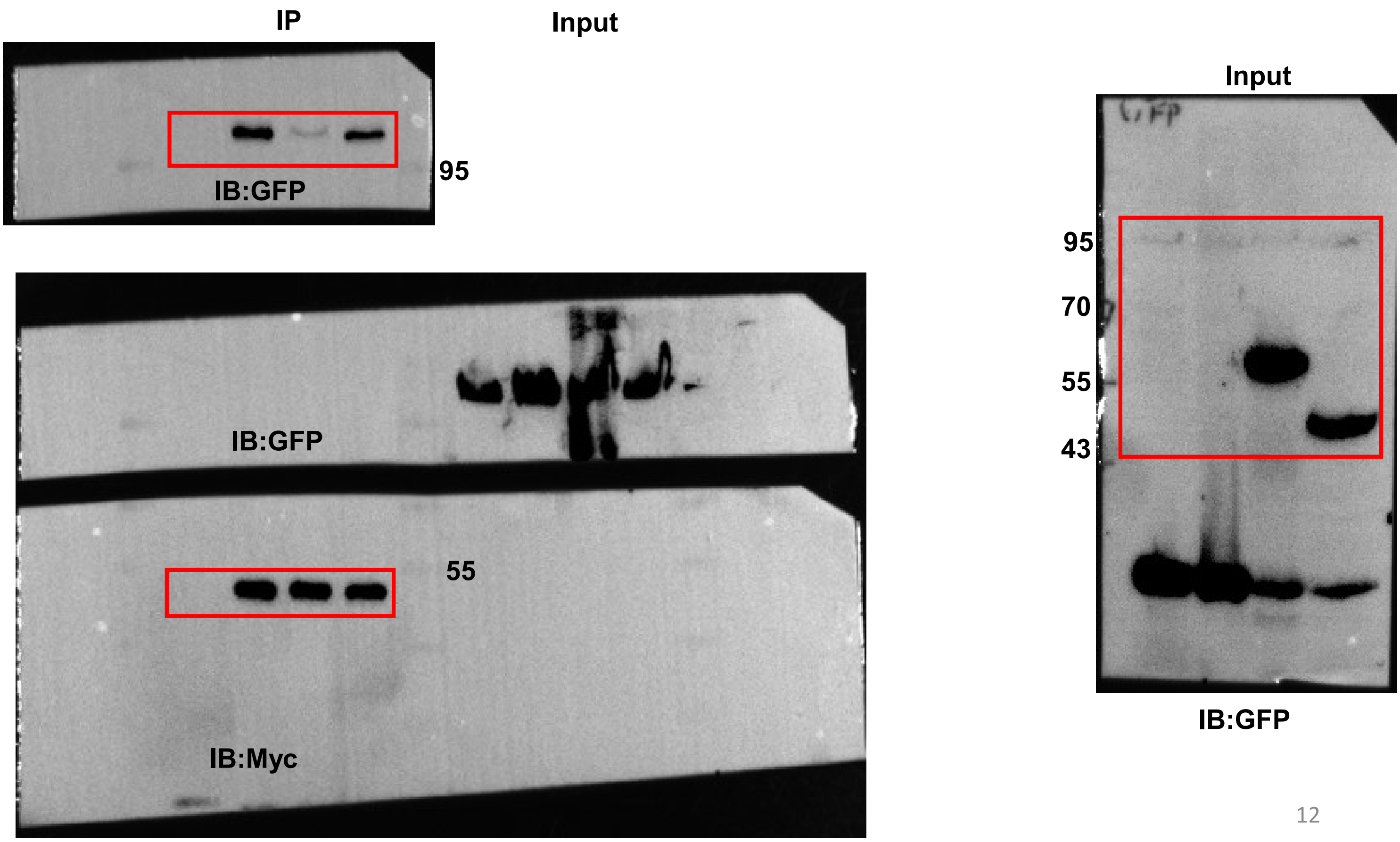

Source Fig. 8L

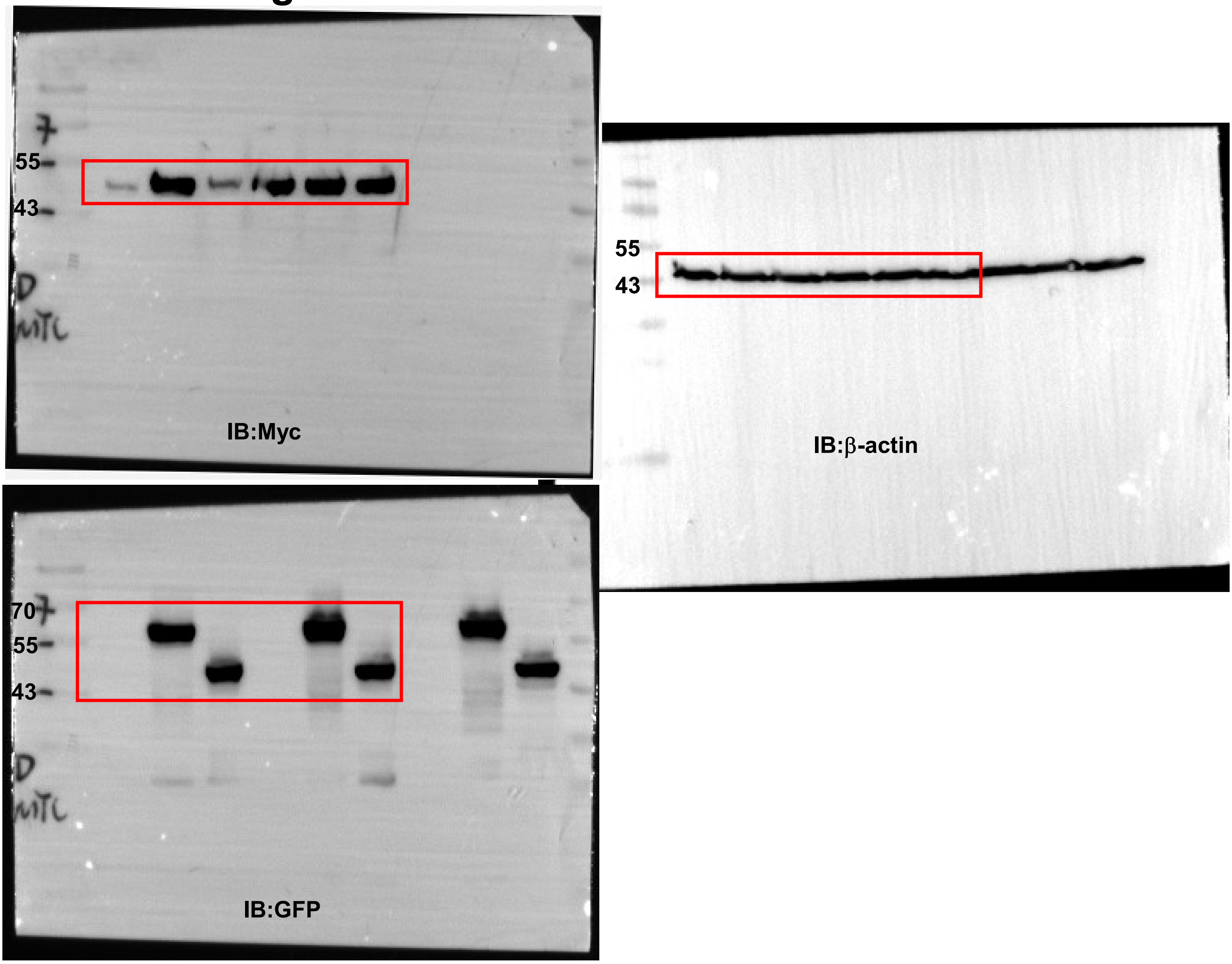

Source Fig. S10A

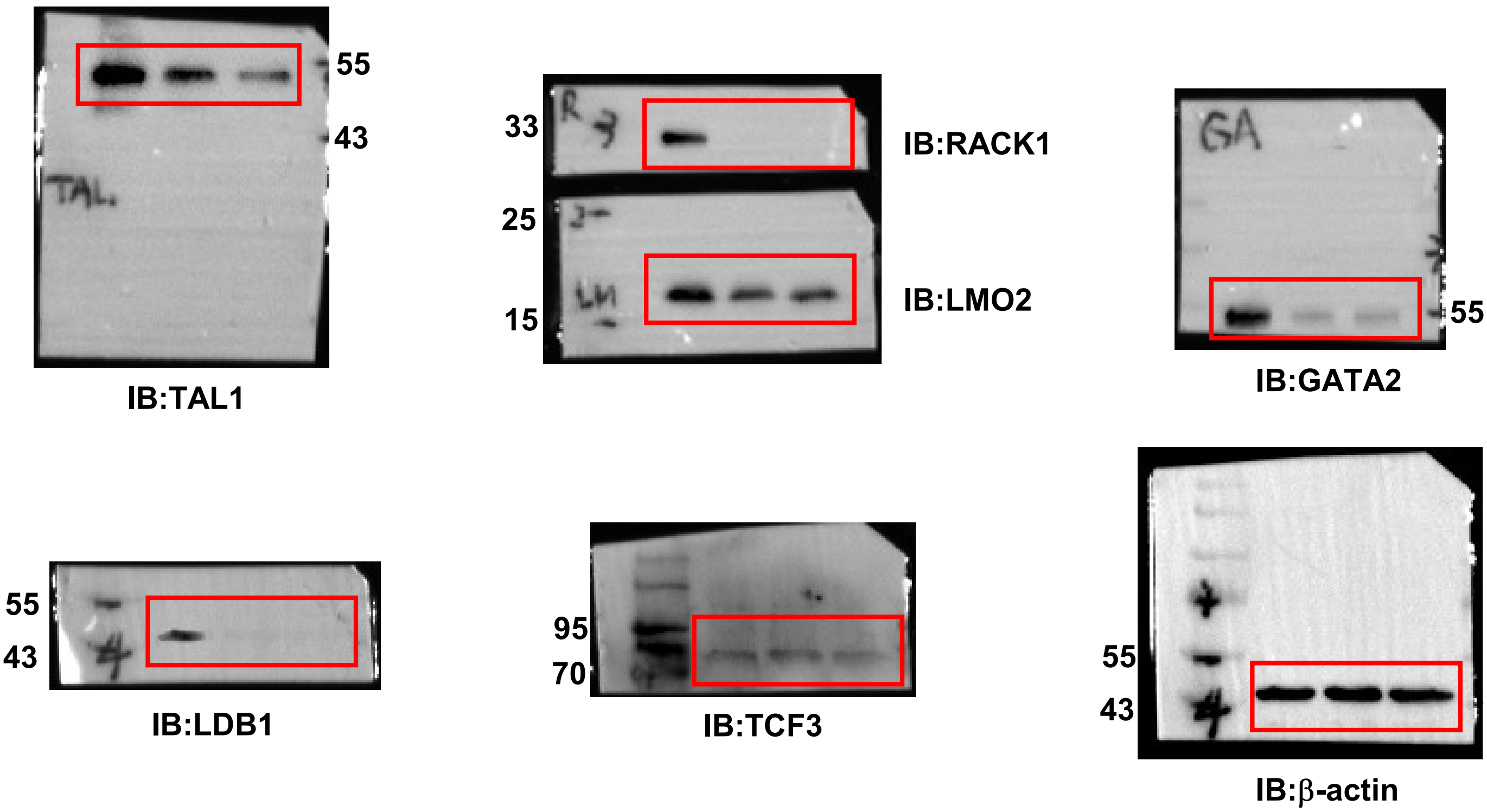

Source Fig. S10D

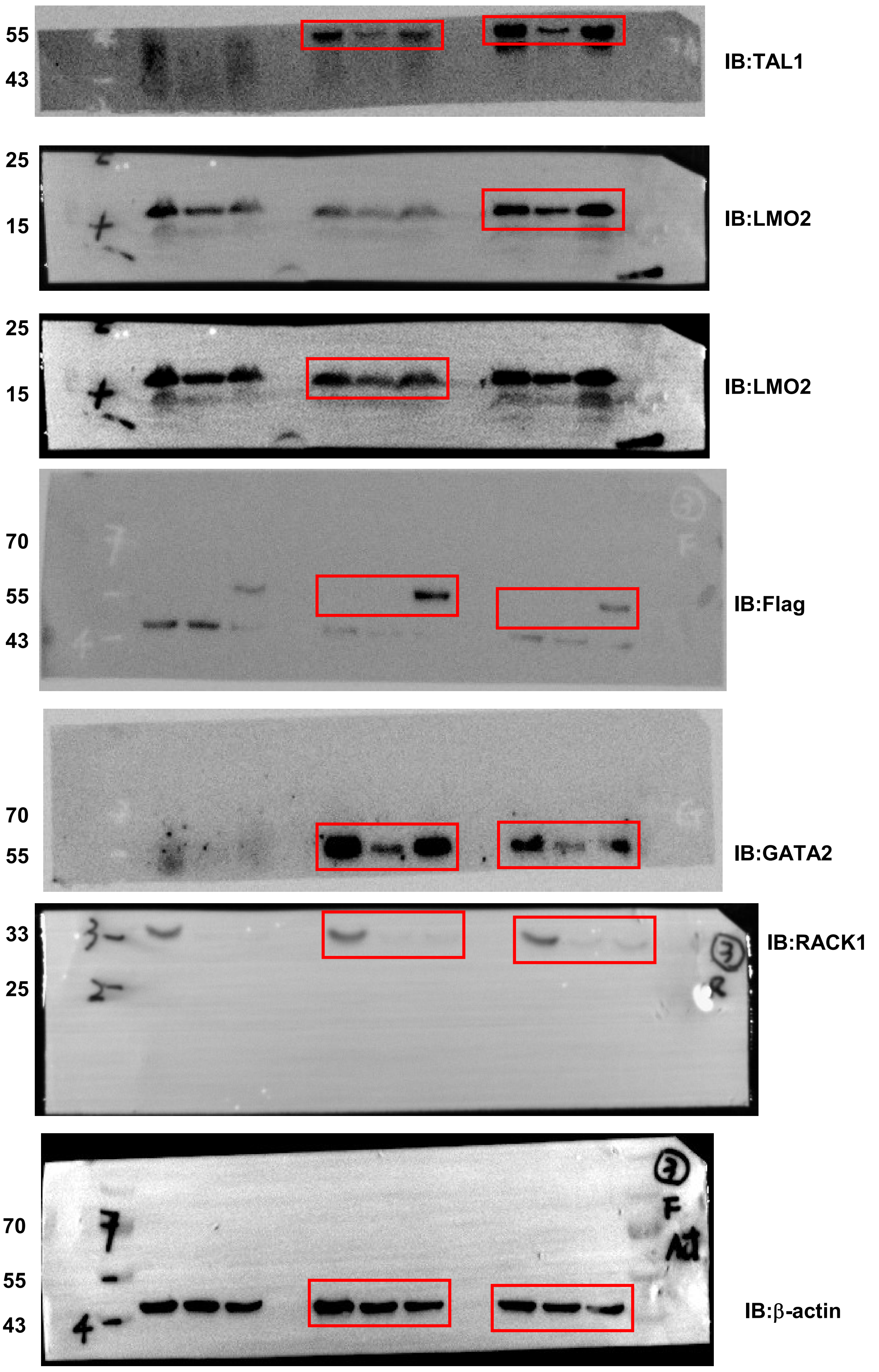

Source Fig. S11A

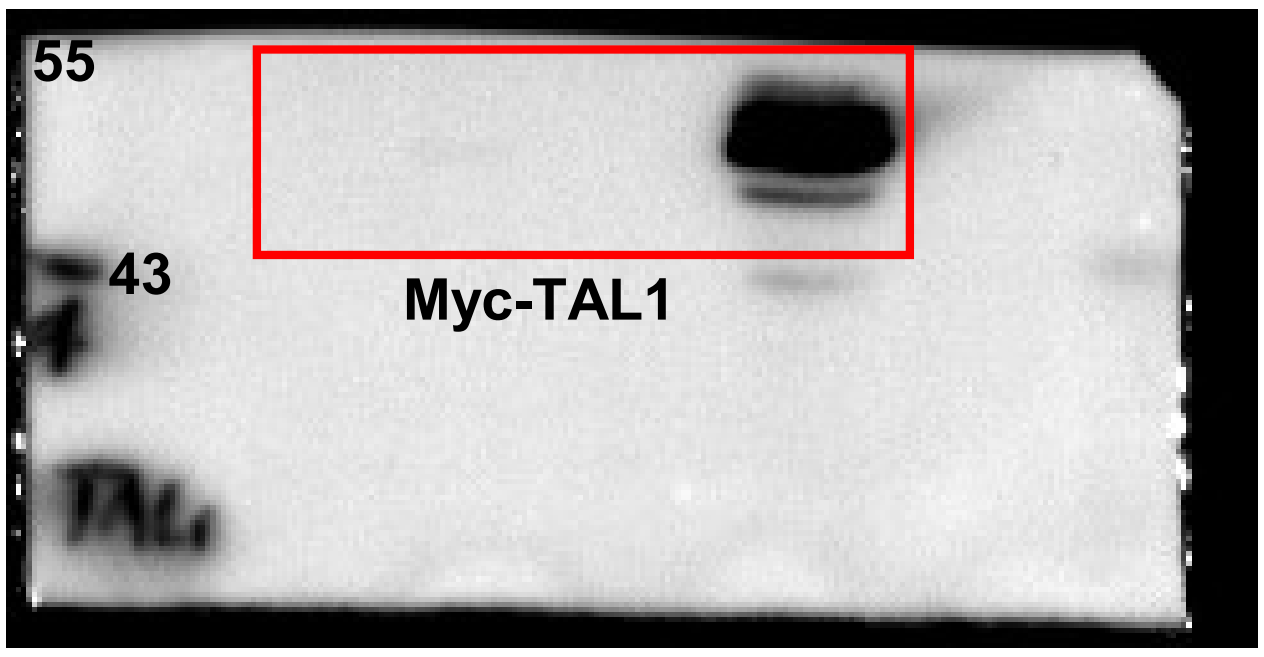

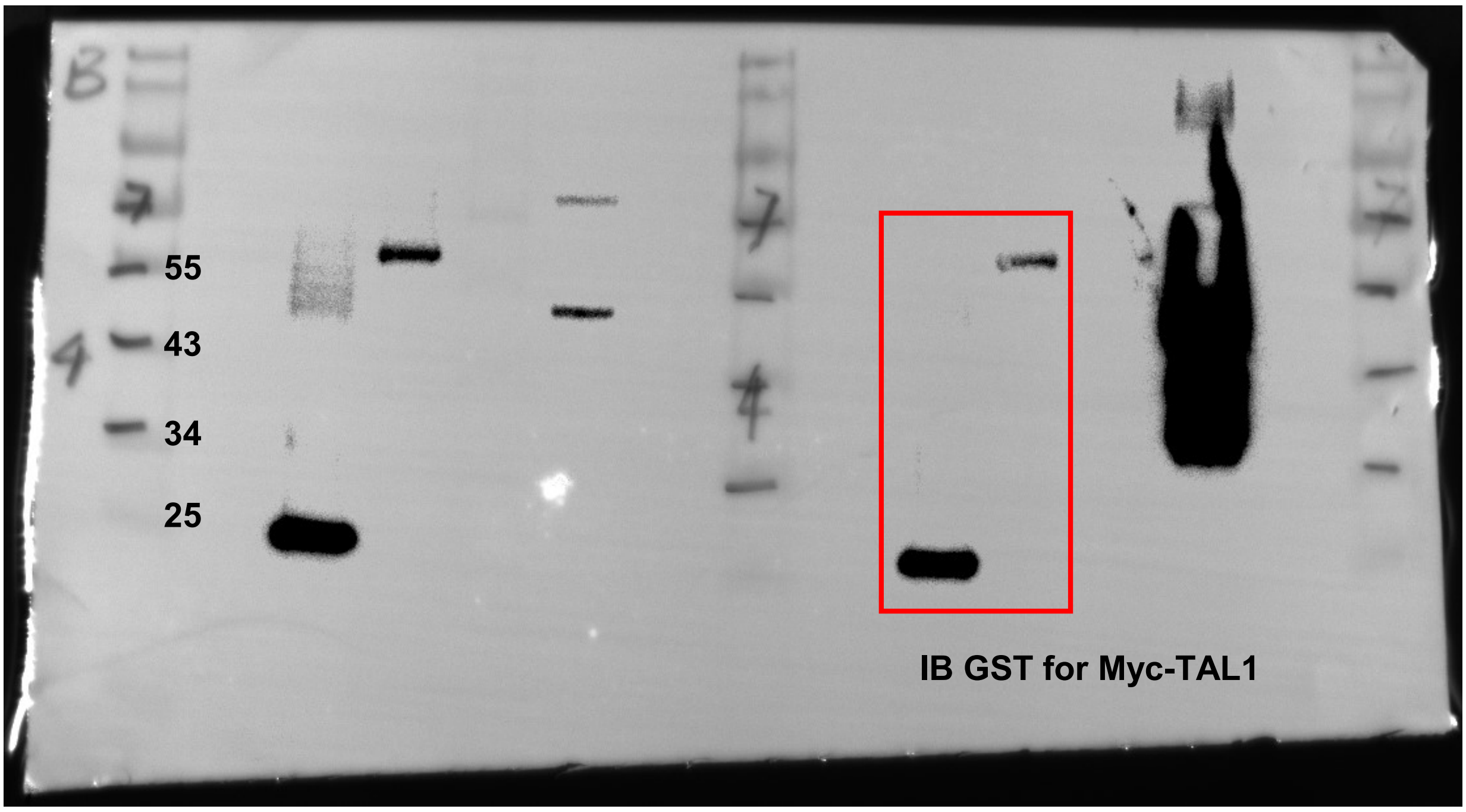

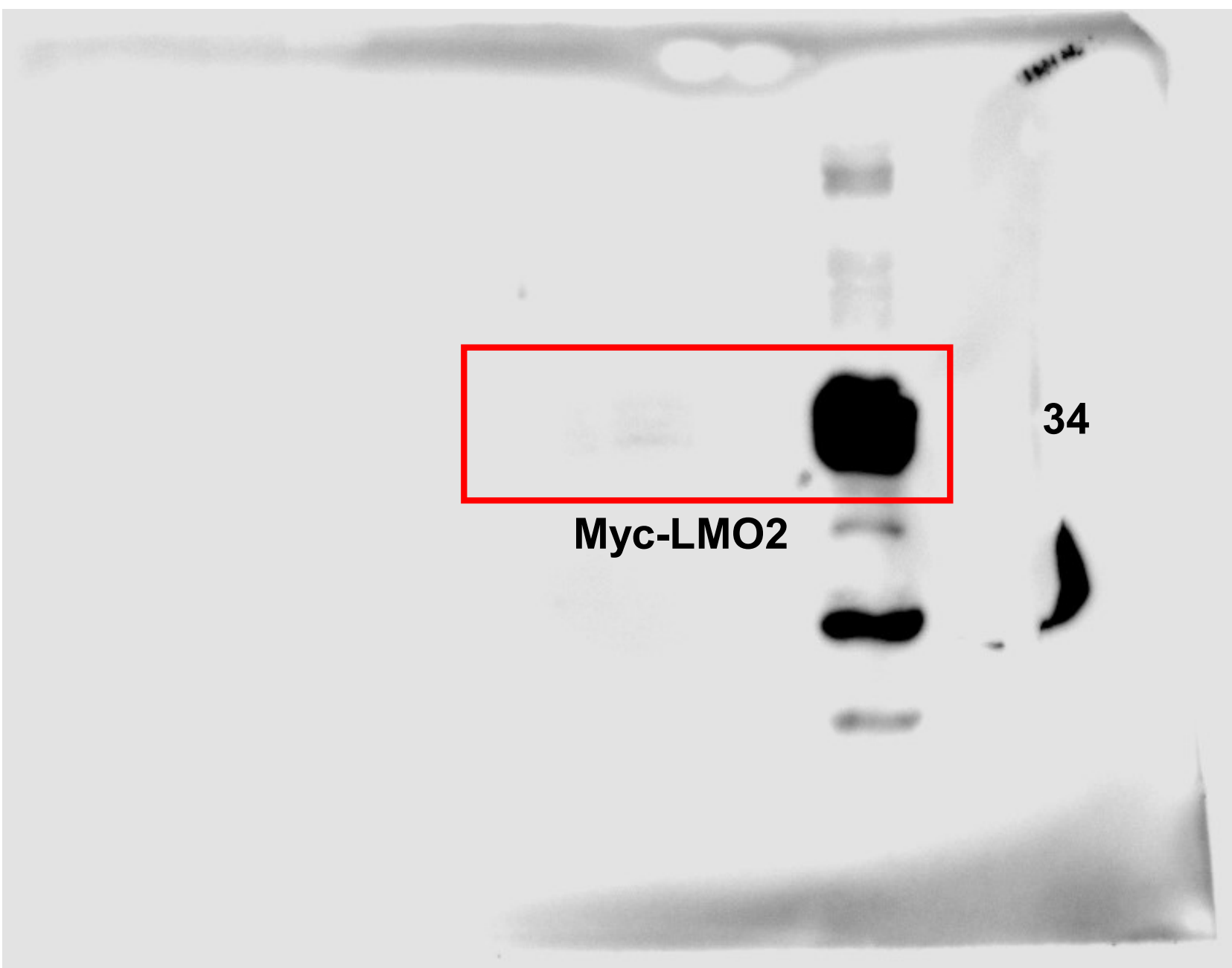

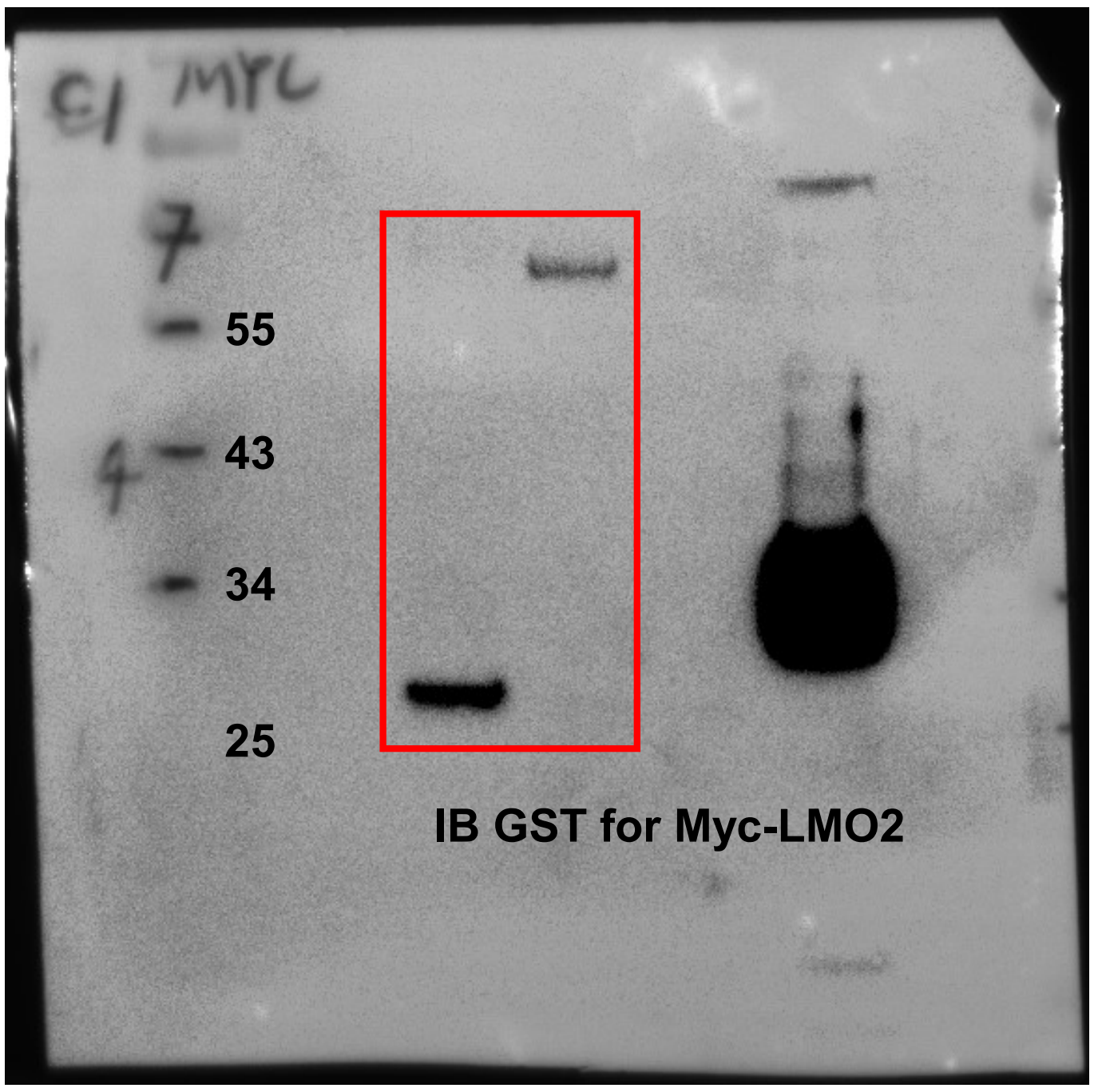

Source Fig. S11A (continued)

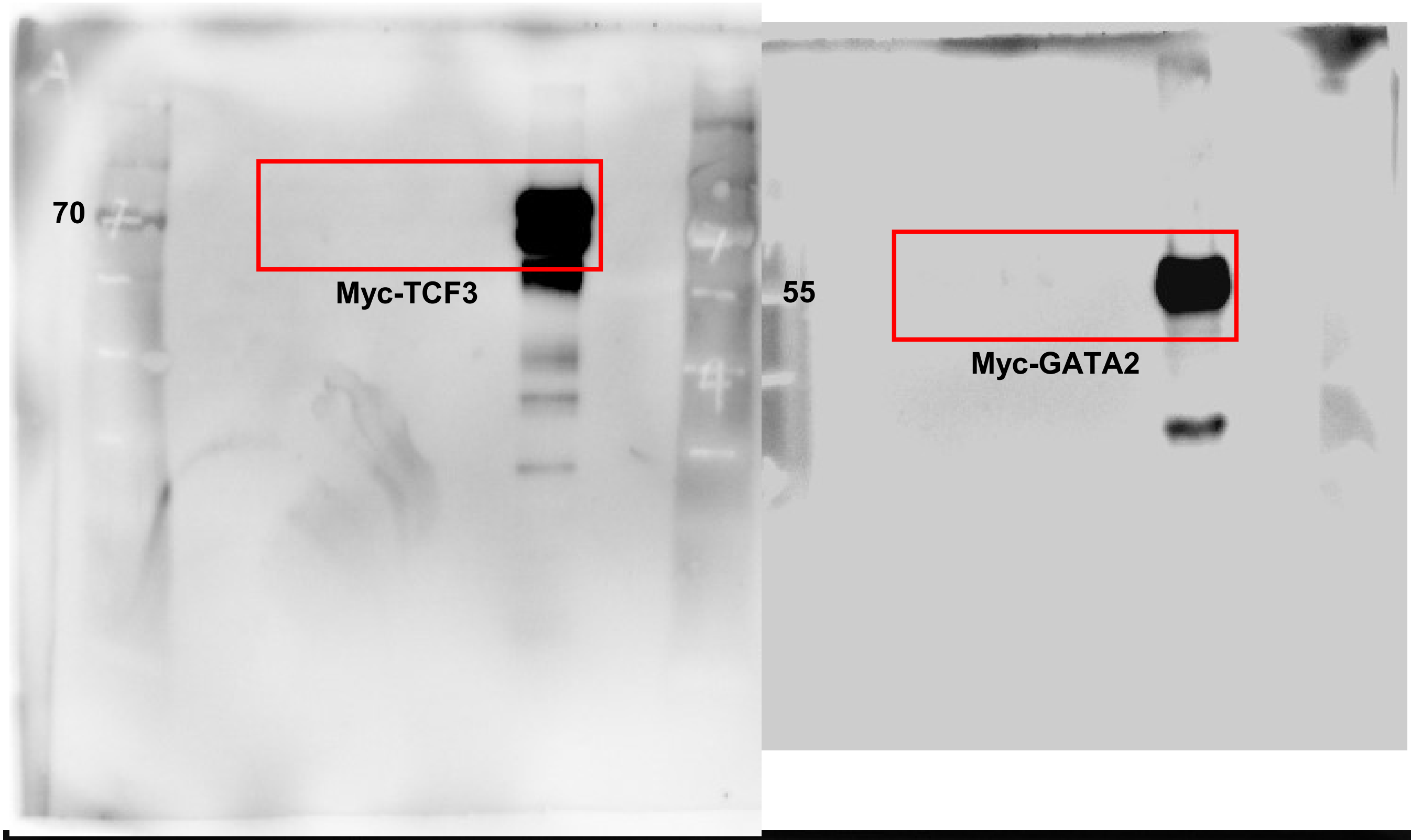

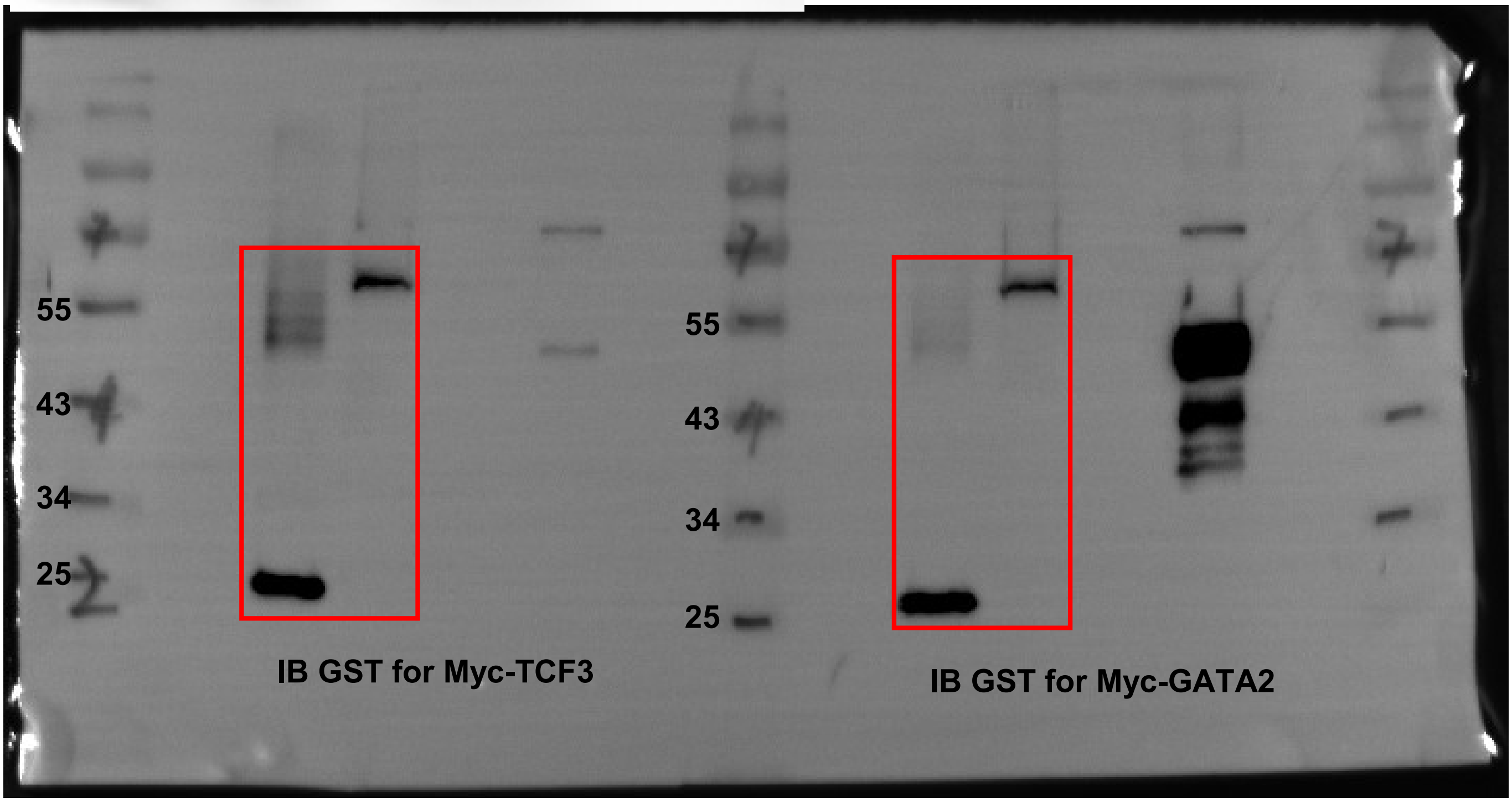

Source Fig. S11B

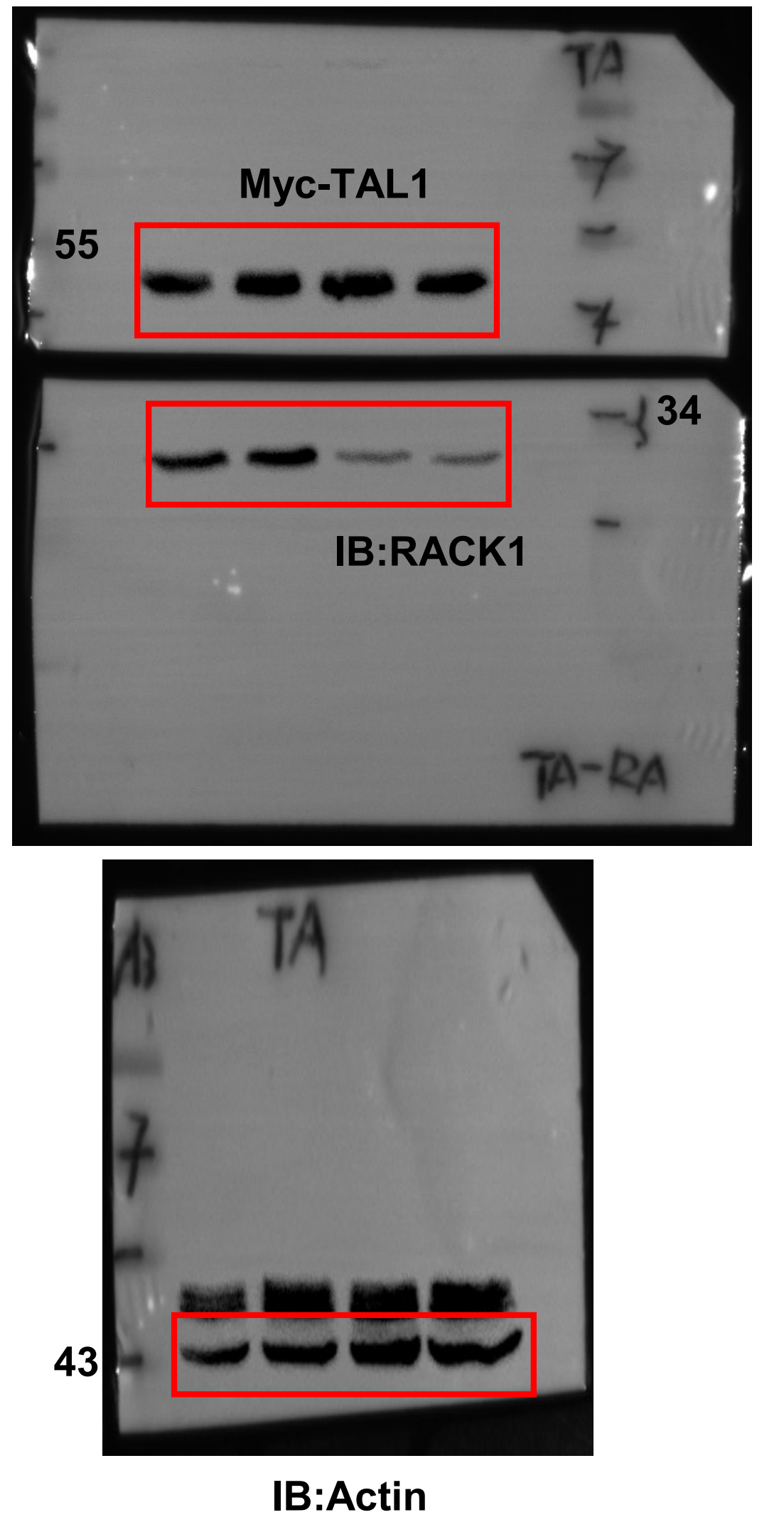

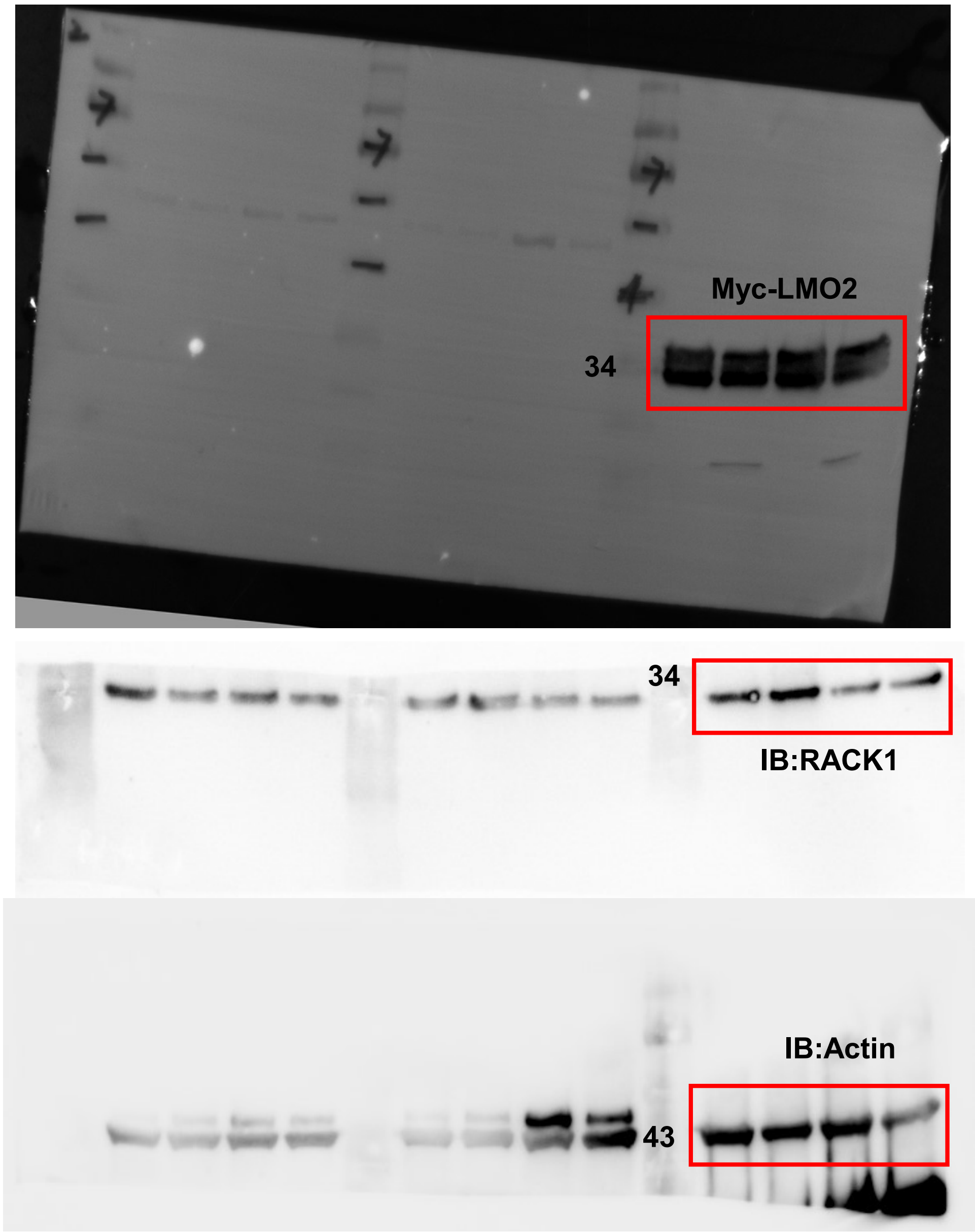

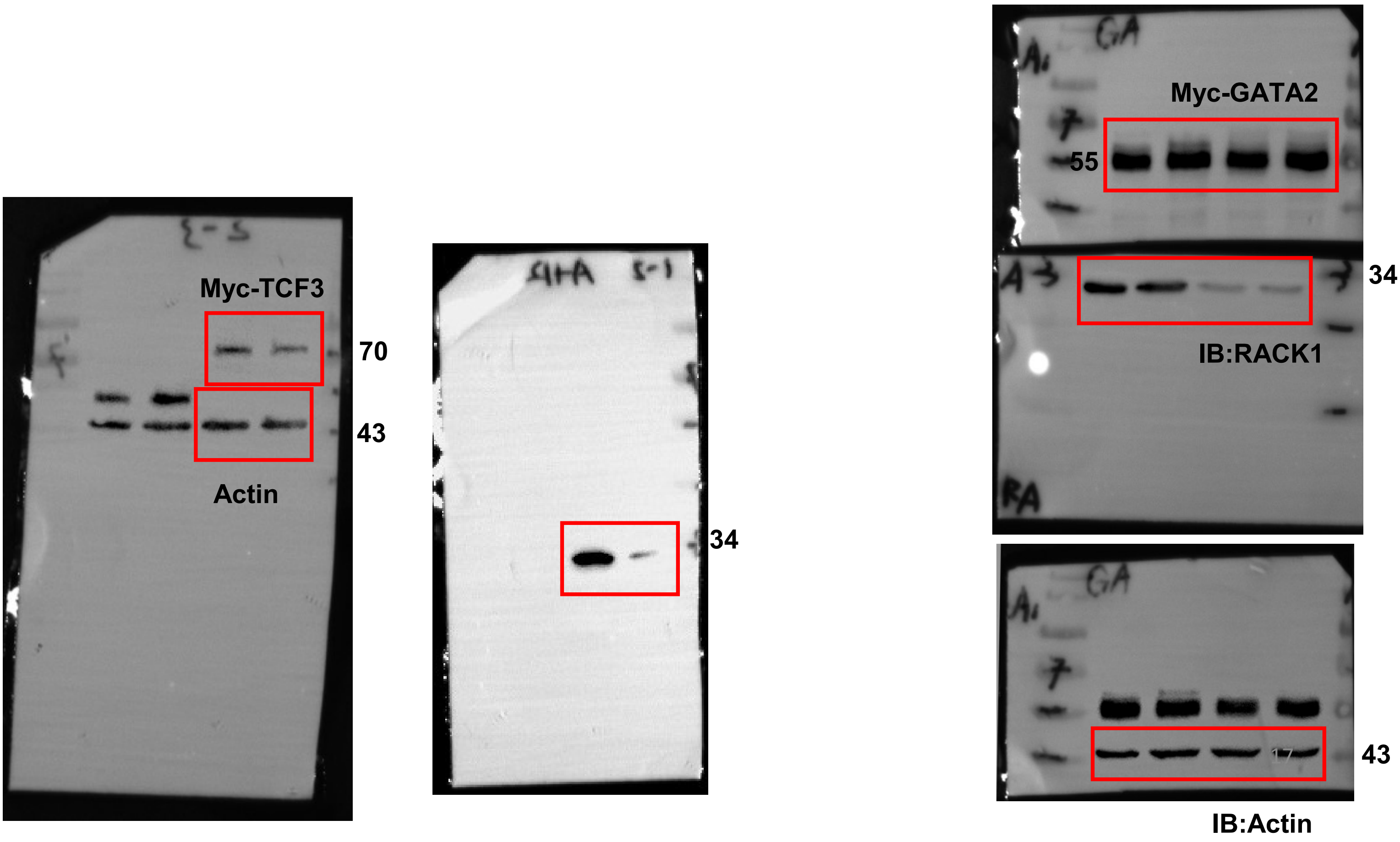

Supplement: Supplementary file 2 [file 41419_2026_9177_MOESM2_ESM.pdf]
